# Genome-wide macroevolutionary signatures of key innovations in butterflies colonizing new host plants

**DOI:** 10.1038/s41467-020-20507-3

**Published:** 2021-01-13

**Authors:** Rémi Allio, Benoit Nabholz, Stefan Wanke, Guillaume Chomicki, Oscar A. Pérez-Escobar, Adam M. Cotton, Anne-Laure Clamens, Gaël J. Kergoat, Felix A. H. Sperling, Fabien L. Condamine

**Affiliations:** 1 CNRS, IRD, EPHE, Institut des Sciences de l’Evolution de Montpellier, Université de Montpellier, Place Eugène Bataillon, 34095 Montpellier, France; 2 Institut für Botanik, Technische Universität Dresden, Zellescher Weg 20b, 01062 Dresden, Germany; 3 Department of Bioscience, Durham University, Stockton Road, Durham, DH1 3LE UK; 4 Royal Botanic Gardens, Kew, TW9 3AB UK; 5 86/2 Moo 5, Tambon Nong Kwai, Hang Dong Chiang Mai, Thailand; 6 CBGP, INRAE, CIRAD, IRD, Montpellier SupAgro, Univ. Montpellier, Montpellier, France; 7 Department of Biological Sciences, University of Alberta, Edmonton, T6G 2E9 AB Canada

**Keywords:** Biodiversity, Biogeography, Evolutionary ecology, Molecular evolution, Phylogenetics

## Abstract

The mega-diversity of herbivorous insects is attributed to their co-evolutionary associations with plants. Despite abundant studies on insect-plant interactions, we do not know whether host-plant shifts have impacted both genomic adaptation and species diversification over geological times. We show that the antagonistic insect-plant interaction between swallowtail butterflies and the highly toxic birthworts began 55 million years ago in Beringia, followed by several major ancient host-plant shifts. This evolutionary framework provides a valuable opportunity for repeated tests of genomic signatures of macroevolutionary changes and estimation of diversification rates across their phylogeny. We find that host-plant shifts in butterflies are associated with both genome-wide adaptive molecular evolution (more genes under positive selection) and repeated bursts of speciation rates, contributing to an increase in global diversification through time. Our study links ecological changes, genome-wide adaptations and macroevolutionary consequences, lending support to the importance of ecological interactions as evolutionary drivers over long time periods.

## Abstract (web summary)

Arms races between herbivores and plants have likely affected their evolutionary histories, which could have led to their high diversity. Allio et al. find that butterflies shifting to new host plants have more adaptive molecular signatures across their genomes and show repeated bursts of speciation rates.

## Introduction

Plants and phytophagous insects account for the majority of the documented species of terrestrial organisms^1,2^. To explain the high diversity of insects, a long held hypothesis states that their diversification is directly related to that of plants^3,4^. More than half a century ago, Ehrlich and Raven^5^ proposed a model in which a continual arms race of attacks by herbivorous insects and new defences by their host plants is linked to species diversification via the creation of new adaptive zones, later termed the ‘escape-and-radiate’ model^6^. According to Ehrlich and Raven^5^, these developments mainly correspond to toxic secondary compounds in plants, and the associated detoxification mechanisms in insects. This model would apply to all plants and plant-eating insects and could explain why these groups represent an important part of global biodiversity^7,8^.

Study of insect–plant interactions has progressed tremendously since then through a focus on host chemistry^9^, phylogenetics^10,11^ and genomics^12–15^. Divergence of key gene families^13–16^ and high speciation rates^17–19^ have been identified after host–plant shifts, with one example linking duplication of key genes to the ability to feed on new plants and increase diversification^13^. The emerging consensus from most phylogenetic studies indicates (1) strong phylogenetic conservatism of host–plant associations (related insect species tend to feed on plants that are also related), suggesting ancient and specialized biotic interactions^20^, and (2) enhanced diversification rates for clades shifting to new host–plant groups compared to those remaining on ancestral plants. Despite high levels of conservatism and specialization, bursts of insect diversification appear to mainly be a consequence of host shifts^21^, and this somewhat paradoxical conclusion can be understood by considering ecological as well as genetic mechanisms behind host shifts^12,15^. There are several ways—both direct and indirect—that interactions can influence speciation^22^, with or without host–plant-based divergent selection on reproductive barriers. One current debate is on the relative importance of radiations following shifts to new adaptive zones and elevated rates of speciation in groups with plastic and diverse host use^23–25^. Increasingly sophisticated use of time-calibrated phylogenies is being made to investigate the actual timing and rate of diversification and to link such events more conclusively to other factors that may have been important, whether biotic or abiotic^18,19^.

Genomic aspects of adaptation by herbivorous insects to their host plants have received significant attention^26^, but few studies have put their genomic data into phylogenetic perspectives. A seminal study by Edger et al.^13^ on the evolutionary arms race between Pierinae butterflies and their Brassicales host plants showed that shifts in diversification within the plants and their butterflies are associated with gradual changes in plant chemical defences and insect molecular counter adaptations. They identified the genomic mechanisms (gene and genome duplications) explaining the evolution of biosynthetic pathways associated with this arms race. More clues for host-encoded digestive and detoxification mechanisms come from a cross-taxonomic comparison of the gut microbiome of caterpillars with other insects and vertebrates^27^. The microbes in caterpillar guts are unusually at low densities, and reflect the abundance and composition of leaf-associated microbes in the caterpillar faeces, with high pH, simple gut structure, and fast transit times potentially preventing microbial colonization.

These recent results have illustrated the need for a multidisciplinary approach to studying the evolution of insect–plant interactions within a macroevolutionary and genomic framework. However, a major knowledge gap lies in our understanding of the evolutionary links and drivers of host–plant shifts, genome-wide signatures of adaptations and processes of species diversification^28^. As noted by Hembry and Weber^29^, this implies that the questions of if, when and how coevolution has an impact on macroevolutionary dynamics remain open challenges. Here we address this gap with an emblematic group that was instrumental in Ehrlich and Raven’s model—the swallowtail butterflies (Lepidoptera: Papilionidae). Swallowtail caterpillars feed on a range of different flowering families^30^, but a third of all species, including the tribes Zerynthiini (Parnassiinae), Luehdorfiini (Parnassiinae) and Troidini (Papilioninae), feeds exclusively on the birthwort family (Aristolochiaceae), which is one of the most toxic plant groups^31^. The Aristolochiaceae notoriously contain toxic aristolochic acids, which are known to be carcinogenic to many organisms, and Papilionidae are among the few that can feed on these plants^32,33^. By eating these toxic plants, the caterpillars sequester aristolochic acids that render both the caterpillars and the adults unpalatable for predators^31^. Interestingly, previous phylogenetic estimations of ancestral states indicated either that Aristolochiaceae was the ancestral host plant of Papilionidae^34^ or that Aristolochiaceae was colonized twice^35^, suggesting that the host–plant shifts have ancient origins and seem to be highly constrained as shown by the high level of host conservatism. Moreover, the arms race between Papilionidae and their host plants has been demonstrated at the molecular level with the evolution of a cytochrome P450 gene that plays a role in the detoxification of secondary plant compounds^36^. Some mutations can bypass the toxic defences of certain plants, providing survival and diversification on certain plants (and not others). Further studies have shown how changes in the use of host plants are associated with changes in the sequence, structure and function of P450. Results provide evidence that new P450 copies can appear for herbivores that colonize new hosts, supporting the hypothesis that interaction between herbivores and their host plants contributed to P450–gene diversification^37^. These studies provide convincing examples of host–plant shifts that may result in increased net diversification rate^18,34^ and specific changes in key genes that confer new abilities to feed on toxic plants^36–38^.

Here, we study the insect–plant interactions at macroevolutionary scale using genomic and diversification approaches within a phylogenetic context. Given the complexity of shifting to a new host plant, we can expect more widespread effects across the entire genome^15,39,40^, but this has remained difficult to demonstrate. Indeed, both comprehensive species-level phylogeny and genomic data are necessary to disentangle the origin of the arms race and to understand the underlying mechanisms of insect–plant interaction as a major driver of diversification. The swallowtail model offers a relevant opportunity to better understand the role played by ecological interactions over the long timescales shaping the astonishing diversity of herbivores^41^.

## Results and discussion

### Co-phylogenetic history of an insect–plant antagonistic interaction

**Fig. 1:**
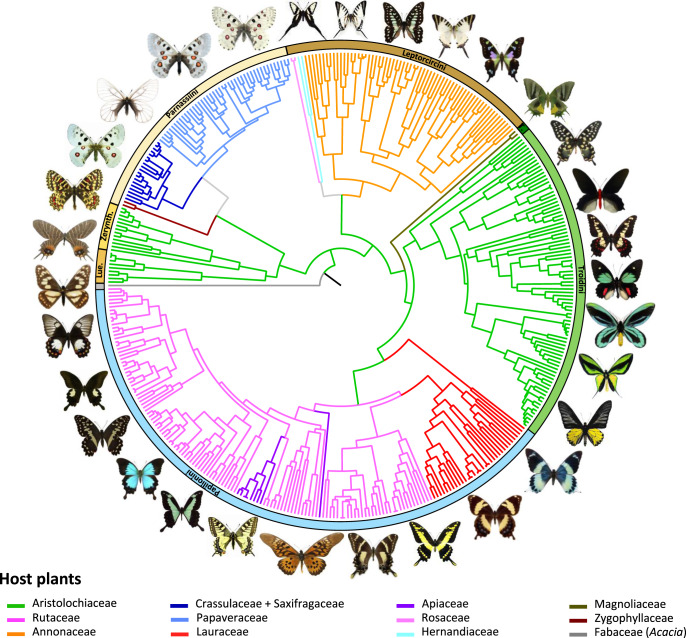
Evolution of host–plant association through time shows strong host–plant conservatism across swallowtail butterflies. Phylogenetic relationships of swallowtail butterflies, with coloured branches mapping the evolution of host–plant association, as inferred by a maximum-likelihood model (Supplementary Figs. 4 and 6). Additional analyses with two other maximum-likelihood and Bayesian models inferred the same host–plant associations across the phylogeny (Supplementary Fig. 5). Lue. Luehdorfiini, Zerynth. Zerynthiini, T. Teinopalpini. Pictures of butterflies made by Fabien Condamine.

First, we created an extensive phylogenetic dataset including seven genetic markers for 71% of swallowtail species diversity (408 of ~570 described species, see ‘Methods’). This dataset leads to the assembly of the most complete and well-resolved dated phylogeny of swallowtail butterflies (79% of nodes with strong bootstrap support defined as ≥95%; Supplementary Figs. 1–3). Both tribe- and genus-level relationships are mostly consistent with previous results using multilocus datasets^18,34,35,42–45^. However, our species tree benefits from a phylogenomic backbone that we recently inferred at the genus level for the Papilionidae using genome-scale data^46^. Second, we compiled host–plant preferences for each swallowtail species in the dataset, and we performed ancestral-state estimations (see ‘Methods’). Phylogenetic estimates of ancestral host–plant preferences indicate that Aristolochiaceae were either the food plant of ancestral Papilionidae^34^ or were colonized twice^35^, suggesting an ancient and highly conserved association with Aristolochiaceae throughout swallowtail butterflies evolution. Using this robust time-calibrated phylogeny (Supplementary Figs. 1–3), we have traced the evolutionary history of food-plant use and infer that the family Aristolochiaceae was the ancestral host for Papilionidae (Fig. 1; relative probabilities = 0.915, 0.789 and 0.787 with three models; Supplementary Figs. 4 and 5). We further show that the genus *Aristolochia* was the ancestral host plant, as almost all Aristolochiaceae-associated swallowtails feed on *Aristolochia* (Supplementary Fig. 6). Across the swallowtail phylogeny, we recover only 14 host–plant shifts at the plant family level (14 nodes out of 407; Supplementary Figs. 4 and 5), suggesting strong evolutionary host–plant conservatism.

**Fig. 2:**
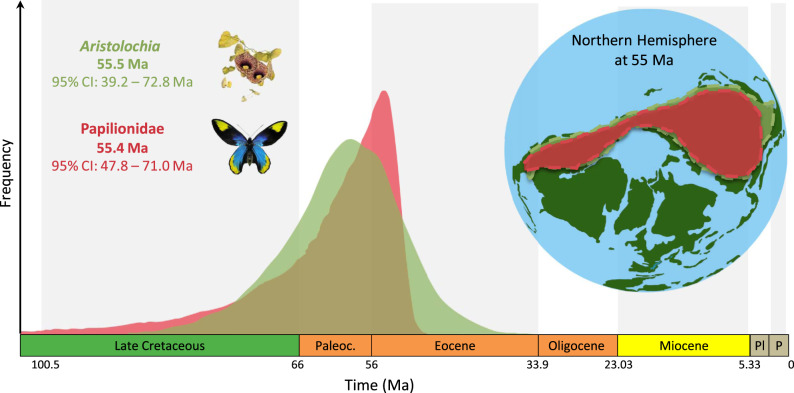
Synchronous temporal and geographic origin for swallowtails and birthworts. Bayesian molecular divergence times with exponential priors estimate an early Eocene origin (~55 Ma) for both swallowtails and *Aristolochia* (alternatively, analyses with a uniform prior estimated an origin around 67 Ma for swallowtails and 64 Ma for *Aristolochia*; Supplementary Figs. 3, 8 and 9). Biogeographical maximum-likelihood models infer an ancestral area of origin comprising West Nearctic, East Palaearctic and Central America for both swallowtails and birthworts (Supplementary Figs. 10 and 11). Paleoc Paleocene, Pl Pliocene, P Pleistocene, Ma million years ago. Pictures of the plant and butterfly made by Fabien Condamine, and the world map made by Rémi Allio.

With the ancestor of swallowtails feeding on birthworts, evidence for synchronous temporal and geographical origins further links the genus *Aristolochia* and the family Papilionidae and supports the escape-and-radiate model. Reconstructions of co-phylogenetic history for other insect–plant antagonistic interactions have shown either synchronous diversification^11^ or herbivore diversification lagging behind that of their host plants^10,47^. We assembled a molecular dataset for ~49% of the species diversity of Aristolochiaceae (247 of ~502 described species; see ‘Methods’) and reconstructed their phylogeny (Supplementary Fig. 7), which is in agreement with previous works^48–52^. Divergence time estimates strongly suggest synchronous radiations of Papilionidae (55.4 million years ago [Ma], 95% credibility intervals (CIs): 47.8–71.0 Ma) and *Aristolochia* (55.5 Ma, 95% CIs: 39.2–72.8 Ma) since the early Eocene (Fig. 2 and Supplementary Figs. 3, 8 and 9). This result is robust to known biases in inferring divergence times, with slightly older ages inferred for both groups when using more conservative priors on clade ages (Supplementary Fig. 9). Such temporal congruence between *Aristolochia* and Papilionidae raises the question of whether both clades had similar geographical origins and dispersal routes. To characterize the macroevolutionary patterns of the *Aristolochia*/Papilionidae arms race in space, we assembled two datasets of current geographic distributions for all species included in the phylogenies of both Aristolochiaceae and Papilionidae. We reconstructed the historical biogeography of both groups, taking into account palaeogeographical events throughout the Cenozoic (see ‘Methods’). Along with the known fossil record of both groups^53–57^, these results suggest that both Papilionidae and *Aristolochia* were ancestrally co-distributed throughout a region, including West Nearctic (WN), East Palaearctic (EP), and Central America (CA) in the early Eocene, when Asia and North America were connected by the Bering land bridge (Fig. 2 and Supplementary Figs. 10 and 11). This combination of close temporal and spatial congruence provides strong evidence that Papilionidae and *Aristolochia* diversified concurrently through time and space until several swallowtail lineages shifted to the new host–plant families in the middle Eocene.

#### Host–plant shifts confer higher rates of diversification

**Fig. 3:**
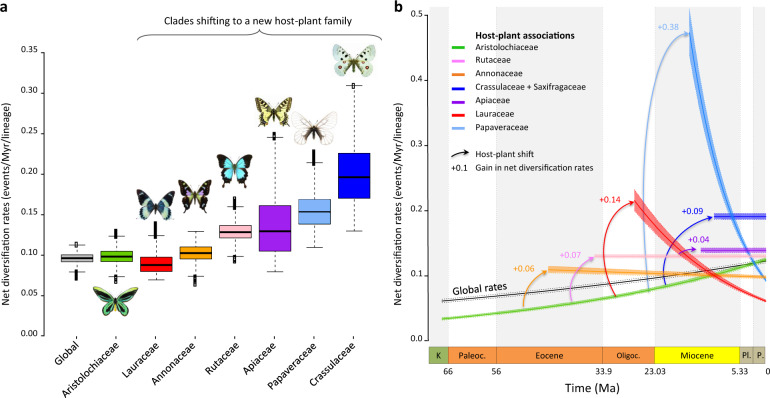
Host–plant shifts lead to repeated bursts in diversification rates and a sustained overall increase in diversification through time. **a** Diversification tends to be higher for clades shifting to new host plants, as estimated by trait-dependent diversification models. Boxplots represent Bayesian estimates of net diversification rates for clades feeding on particular host plants (see also Supplementary Fig. 12). **b** A global increase in diversification is recovered with birth–death models estimating time-dependent diversification (see also Supplementary Figs. 14 and 15). Taking into account rate heterogeneity by estimating host–plant and clade-specific diversification indicates positive gains of net diversification after shifting to new host plants (see also Supplementary Fig. 13). K Cretaceous, Paleoc. Paleocene, Oligoc. Oligocene, Pl Pliocene, P Pleistocene, Ma million years ago. Pictures of butterflies made by Fabien Condamine.

Our ancestral-state estimates and biogeographic analyses are consistent with a sustained arms race between *Aristolochia* and Papilionidae in the past 55 million years. According to the escape-and-radiate model, a host–plant shift should confer higher rates of species diversification for herbivores through the acquisition of novel resources to radiate into^5,6^ and/or the lack of competitors (Aristolochiaceae-feeder swallowtails have almost no competitors^31^). We tested the hypothesis that increases in diversification rates occurred in swallowtail lineages that shifted to new host plants. Given the uncertainty surrounding the inferences of macroevolutionary rates from phylogenies of extant species, we applied a suite of birth–death models to cross-validate the estimated rates of diversification LASER (Likelihood Analysis of Speciation and Extinction Rates), MuSSE (Multiple State Speciation Extinction), RPANDA (R: Phylogenetic ANalyses of DiversificAtion), BAMM (Bayesian analysis of macroevolutionary mixtures), CoMET (CPP on Mass-Extinction Times) and RevBayes; see ‘Methods’). We find evidence for (1) increases of diversification at host–plant shifts with trait-dependent birth–death models (as inferred with: MuSSE, Fig. 3a and Supplementary Fig. 12; RPANDA, Supplementary Fig. 13; and LASER, Supplementary Table 1) and (2) host–plant shifts contributing to a global increase through time with clade- and time-dependent birth–death models (as inferred with: RPANDA, Fig. 3b and Supplementary Fig. 13; BAMM, Supplementary Fig. 14; RevBayes, Supplementary Fig. 15; and CoMET, Supplementary Fig. 16). Although we should be cautious about the estimations of macroevolutionary rates^58–63^, all models concur that diversification rates increase through time either globally or due to recurrent host–plant shifts. Interestingly, these results contrast with the slowdown of diversification that is classically recovered in most phylogenies, often attributed to ecological limits and niche filling processes^63^. This sustained and increasing diversification during the Cenozoic may be explained by ecological opportunities not decreasing, due to a steady increase in host breadth for Papilionidae with new host–plant families colonized through time (Supplementary Fig. 17). Opening up new niches, which can also expand due to diversification increases of the host–plant families through time^64–66^, would allow a continuous increase in diversification rates through time in a dynamic biotic environment, lending support to the primary role of ecological interactions in clade diversification over long timescales—a long-contentious issue^29^. Nonetheless, when taking into account the possibility that rates may have been heterogeneous across the phylogeny, we find that the diversification of three lineages (those feeding on Annonaceae, Lauraceae and Papaveraceae) had early rates of speciation that are higher than the ancestral rates, but slowed down through time.

Interestingly, not all host–plant shifts led to evolutionary success in terms of extant species diversity. Given our rate estimations, we found significantly lower diversification rates than the rates on the ancestral host–plant Aristolochiaceae for three host–plant shifts (to Fabaceae, Magnoliaceae and to Zygophyllaceae; Supplementary Fig. 12). Altogether, these three host switches correspond to a very low proportion (~1%) of the total swallowtail diversity today. Indeed, a single species (*Baronia brevicornis*) feeds on the Fabaceae, the genus *Hypermnestra* (two species) feeds on Zygophyllaceae and the genus *Teinopalpus* (two species) feeds on Magnoliaceae. Hence, these are unsuccessful host–plant shifts from an evolutionary perspective (i.e. evolutionary dead-ends).

### Genome-wide adaptations to host–plant shifts

Key innovations are often considered to underlie ecological opportunities and/or evolutionary success^67^, particularly in the case of chemically mediated interactions between butterflies and their host plants^13^. Studies on Papilionidae have provided strong examples of specific changes in key genes that confer new abilities to feed on toxic plants and allow host–plant shifts^36,37^. Adaptations of swallowtails to their hosts have particularly been assessed through the study of cytochrome P450 monooxygenases (P450s), which have a major role in detoxifying secondary plant compounds. New P450s appear to arise in swallowtails that colonize new hosts to bypass toxic defences, providing survival and diversification on some but not all plants^15,36,37^. This supports the hypothesis that insect–plant interactions contributed to P450-gene family diversification, with P450s being key innovations that explain the evolutionary and ecological success of phytophagous insects^14,15,36,38,68,69^. However, host–plant shifts not only alter single genes but may also influence unlinked genes^40^. Moreover, host–plant shifts can accompany changes of the abiotic environment, which may, in turn, require further biotic adaptation (new predators and/or competitors). But the macroevolutionary and genomic consequences of the evolutionary dynamics of host–plant shifts have not yet been demonstrated.

**Fig. 4:**
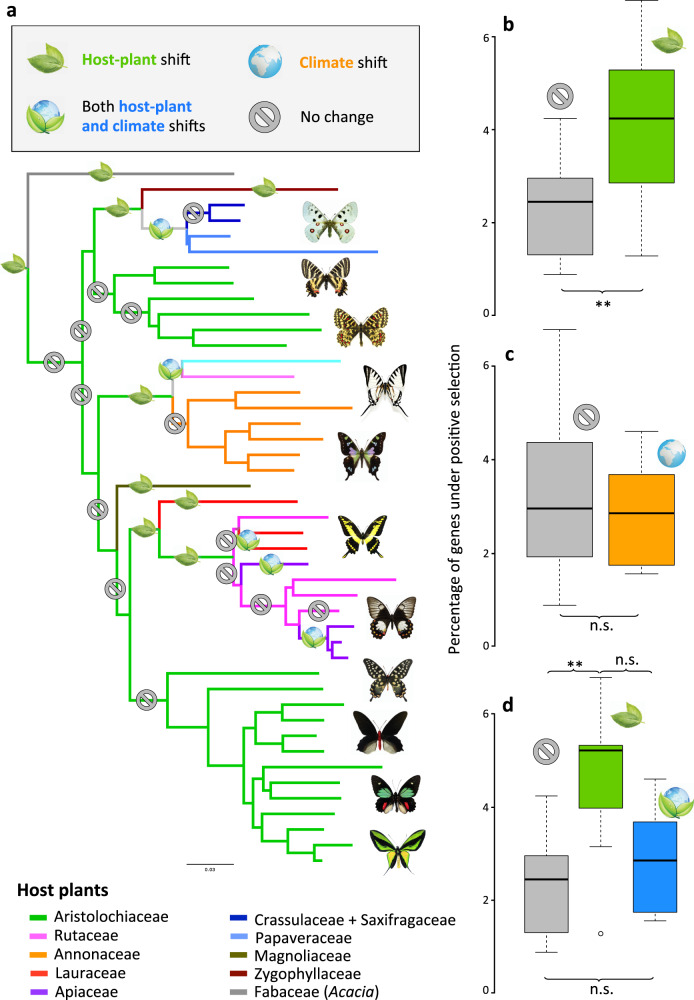
Host–plant shifts promote higher molecular adaptations. **a** Genus-level phylogenomic tree displaying branches with and without host–plant shifts, on which genome-wide analyses of molecular evolution are performed. **b** Number of genes under positive selection (dN/dS > 1) for swallowtail lineages shifting to new host–plant families (*n* = 14, green) or not (*n* = 14, grey). **c** Number of genes under positive selection for swallowtail lineages undergoing climate shifts (*n* = 5, orange) or not (*n* = 23, grey). **d** Number of genes under positive selection for swallowtail lineages shifting to new host plants (*n* = 9, green), shifting both host–plant and climate (*n* = 5, blue) or not (*n* = 14, grey). The proportion of genes was estimated with Dataset 2 (1533 genes, see Supplementary Fig. 19 for the results with Dataset 1 and 520 genes). This demonstrates genome-wide signatures of adaptations in swallowtail lineages shifting to new host–plant families. Genes under positive selection did not contain over- or under-represented functional GO categories (Supplementary Data 2). Wilcoxon rank-sum test: n.s. = not significant (*P* > 0.05), *P ≤ 0.05, **P ≤ 0.01. Pictures and icons made by Fabien Condamine.

Relying on a genomic dataset comprising 45 genomes covering all swallowtail genera^46,70–72^, we constructed two specific datasets (Dataset 1: 520 genes and Dataset 2: 1533 genes; mean gene coverage = 26.7×; see ‘Methods’ and Supplementary Data 1). To test whether there are any genomic signatures of positive selection caused by host–plant shifts within swallowtails, we performed a comparative genomic survey of molecular adaptation between swallowtail lineages that shifted to new host plants compared to non-shifting lineages (see ‘Methods’). We selected 14 phylogenetic branches representing a host–plant shift and 14 phylogenetic branches with no change as negative controls^73,74^ (Fig. 4a). For a fair molecular comparison, each branch selected as a negative control was chosen to be as close as possible to a test branch representing a host–plant shift (i.e. sister groups; Supplementary Fig. 18). Among branches with host–plant shifts, five branches also had a shift in climate preference (represented by distributional changes from tropical to temperate conditions). Using a maximum-likelihood (ML) method, we estimated the ratio of non-synonymous substitutions (dN) other synonymous substitutions (dS) in all branches where a host–plant shift was identified relative to branches with no host–plant shift^75,76^ (see ‘Methods’). The dN/dS analyses on branches with host–plant shifts (combined or not with environmental shifts) showed more genes with a subset of codons evolving under positive selection (dN/dS > 1) in lineages shifting to a new plant family, although the difference was marginally non-significant for the smallest dataset and highly significant for the second dataset containing more genes (Fig. 4b, Supplementary Fig. S19 and Supplementary Table 2, *P* = 0.0501/0.0079 for the two datasets, respectively, Wilcoxon rank-sum test, see ‘Methods’ for the definition of the datasets). However, dN/dS analyses on branches with environmental shifts indicated a balanced number of genes under positive selection (Fig. 4c, Supplementary Fig. S19 and Supplementary Table 2, *P* = 0.336/0.8162 for the two datasets, respectively, Wilcoxon rank-sum test), suggesting a lower impact of environmental shifts than host–plant shifts. We then performed dN/dS analyses for branches with host–plant shifts only (not followed by environmental shifts) and found that swallowtail lineages shifting to a new host–plant family had significantly more genes under positive selection (4.41%/3.98% of genes under positive selection for the two datasets, respectively; Supplementary Table 2) than non-shifting lineages (3.02%/2.43% of genes under positive selection for the two datasets, respectively, Fig. 4d, Supplementary Fig. S19 and Supplementary Table 2, *P* = 0.0071/0.00156 for the two datasets, respectively, Wilcoxon rank-sum test). Surprisingly, the dual changes in environment and host–plant preferences did not spur molecular adaptation across swallowtail lineages compared to control branches (*P* = 1/0.4439 for the two datasets, respectively, Wilcoxon rank-sum test; Fig. 4d, Supplementary Fig. S19 and Supplementary Table 2). Comparing the proportion of genes under positive selection between the branches with dual changes and branches with host–plant shifts only shows a marginally significant difference with Dataset 1 and no difference with Dataset 2 (*P* = 0.0327/0.1471 for the two datasets, respectively, Wilcoxon rank-sum test; Fig. 4d and Supplementary Fig. S19). However, this result might be an artefact due to the use of a few branches to perform the statistical comparison. Although we did not control for the effect of multi-nucleotide mutations^77^, which should affect dN/dS analyses equally for control and host–plant shift branches, we checked individually the gene alignments and performed sensitivity analyses, which showed that our results are not driven either by an excess of misaligned regions or missing data and GC-content variations among species (see ‘Methods’ and Supplementary Figs. 20–26). Finally, given that fixing the topology for CodeML (see ‘Methods’) can spuriously inflate substitution rates on some branches^78^, we computed the proportion of genes under positive selection by selecting the gene trees from the largest dataset (Dataset 2) for which the focal branches were recovered (in agreement with the species tree). These analyses confirmed the previous results suggesting more genes under positive selection during host–plant shifts (*P* = 0.0444, Wilcoxon rank test; Supplementary Table 2).

We further studied the functional categories of positively selected genes by using gene ontology (GO) analyses (PANTHER and EggNOG; see ‘Methods’). Applied to the high-quality genomes of *Papilio xuthus*^71^ and *Heliconius melpomene*^79^, we found that ~70% of the genes could be associated with a gene function and ~30% lacked annotation, which suggests a gap of knowledge in the current insect database of gene function. Among the annotated genes, we found that genes under positive selection along branches with host shifts did not contain over- or under-represented functional GO categories: 252 out of 1213 GO categories represented by genes under positive selection (*P* > 0.05, Fisher’s exact test after false discovery rate correction; Supplementary Data 2). These results support the hypothesis that genome-wide signatures of adaptations are associated with host–plant shifts, and encourage enlarging the hypothesis that changes in only one or a few candidate gene families could be enough to act as key innovations for adaptation to new resources^13,17^. Despite a weak signal, it is striking that host–plant shifts left stronger genome-wide signatures than were associated with changing climate preferences. This result further suggests that the success of phytophagous insects involved widespread adaptations to biotic interactions than for shifts in the abiotic environment.

To conclude, establishing evolutionary links between ecological adaptations, genomic changes and species diversification over geological timescales remains a tremendous challenge^28,80,81^ with, for instance, important limitations due to the lack of knowledge in functional gene annotations in insects. However, the successful development of powerful analytical tools in conjunction with the increasing availability of insect genomes and improvements in genomic analyses^82^ have allowed the detection of more genes than those already known to be involved in detoxification pathways playing a role in long-term relationships between plants and insects. Our genome-wide analyses have also generated a list of candidate genes potentially involved in plant–insect interactions. This opens new research avenues for finding the functionality of genes potentially linked with the adaptation and diversification of phytophagous insects. We hope that our study will help move in that direction, and that it will provide perspectives for future investigations of other model groups.

Over a half-century ago, Ehrlich and Raven^5^ proposed that insect–plant interactions driven by diffuse coevolution over long evolutionary periods can be a major source of terrestrial biodiversity. Applied to a widely appreciated case in the insect–plant interactions theory, our study has been able to investigate genome-wide adaptive processes and corresponding macroevolutionary consequences in a comprehensive framework, suggesting that more genes could be involved in host–plant shifts than previously studied in the diversification of herbivorous insects. This result confirms the general belief in the insect–plant community that host–plant shifts are complex and would thus require a number of adaptations, which likely affect various genes beyond those directly linked to detoxification of the plant compounds^36,39,40^. By expanding the possible genes and gene families and identifying more adaptations than those gene families in detoxification pathways that were detected through antagonist interactions^39^, we show genomically wide-ranging co-evolutionary consequences^40,83^ for close relationships between insects and their larval host plants. Hence, genome-wide macroevolutionary consequences of key adaptations in new insect–plant interactions may be a general feature of the co-evolutionary interactions that have generated Earth’s diversity.

## Methods

### Time-calibrated phylogeny of Papilionidae

We assembled a supermatrix dataset with available data extracted from GenBank as of May 2017 (most of which has been generated by our research group), using five mitochondrial genes (*COI*, *COII*, *ND1*, *ND5* and *rRNA 16S*) and two nuclear markers (*EF-1a* and *Wg*) for 408 Papilionidae species (~71% of the total species diversity) and 20 outgroup species. We aligned the DNA sequences for each gene using MAFFT 7.110^84^ with default settings (E-INS-i algorithm), and the alignments were checked for codon stops and eventually refined by eye with Mesquite 3.1 (available at: www.mesquiteproject.org). The best-fit partitioning schemes and substitution models for phylogenetic analyses were determined with PartitionFinder 2.1.1^85^ using the *greedy* search algorithm and the Bayesian Information Criterion. All gene alignments were concatenated in a supermatrix, which is available in Figshare (see Data availability).

Phylogenetic relationships were estimated with both ML and Bayesian inference. ML analyses were carried out with IQ-TREE 1.6.8^86^. We set the best-fit partitioning scheme (-ssp option) and used ModelFinder to determine the best-fit substitution model for each partition^87^ and then estimated model parameters separately for every partition^88^ such that all partitions shared the same set of branch lengths, but we allowed each partition to have its own evolution rate (-m TESTNEW option). For tree search parameters, we relied on a more thorough and slower nearest-neighbour interchange search to consider all possible nearest-neighbour interchanges instead of only those in the vicinity previously applied (-allnni option). Following the recommendation of IQ-TREE developers, we also set smaller perturbation strength (-pers 0.2) and a larger number of stop iterations (-nstop 500) to avoid local optima. We performed 2000 ultrafast bootstrap replicates to investigate nodal support across the topology, considering values ≥95 as strongly supported nodes^89^.

Estimating phylogenetic relationships for such a dataset is computationally intensive with Bayesian inference. The ML tree inferred with IQ-TREE was used as a starting tree for Bayesian inference as implemented in MrBayes 3.2.6^90^. Rather than using a single substitution model per molecular partition, we sampled across the entire substitution-model space^91^ using reversible-jump Markov Chain Monte Carlo (rj-MCMC). Two independent analyses with one cold chain and seven heated chains, each run for 50 million generations, sampled every 5000 generations. Convergence and performance of Bayesian runs were evaluated using Tracer 1.7.1^92^, the average deviation of split frequencies (ADSFs) between runs, the effective sample size (ESS) and the potential scale reduction factor (PSRF) values for each parameter. The runs had to have values of ADSF approaching zero, PSRF close to 1.0 and ESS >200 to be considered convergent. A 50% majority-rule consensus tree was built after conservatively discarding 25% of sampled trees as burn-in. Node support was evaluated with posterior probability considering values ≥0.95 as strong support^93^. All analyses were performed on the CIPRES Science Gateway computer cluster^94^, using BEAGLE^95^.

Dating inferences were performed using Bayesian relaxed-clock methods accounting for rate variation across lineages^96^. MCMC analyses implemented in BEAST 1.8.4^97^ were employed to approximate the posterior distribution of rates and divergences times and infer their CIs. Estimation of divergence times relied on constraining clade ages through fossil calibrations. Swallowtail fossils are scarce, but five can unambiguously be attributed to the family. The oldest fossil occurrences of Papilionidae are the fossils †*Praepapilio colorado* and †*Praepapilio gracilis*^53^, both from the Green River Formation (Colorado, USA). The Green River Formation encompasses a 5 million years period between ~48.5 and 53.5 Ma, which falls within the Ypresian (47.8–56 Ma) in the early Eocene^98^. These fossils can be phylogenetically placed at the crown of the family as they share synapomorphies with all extant subfamilies^55,99^, and have proven to be reliable calibration points for the crown group^18,34,46^. Two other fossils belong to Parnassiinae, whose systematic position was assessed using phylogenetic analyses based on both morphological and molecular data in a total-evidence approach^18^. The first is †*Thaites ruminiana*^100^, a compression fossil from limestone in the Niveau du gypse d’Aix Formation of France (Bouches-du-Rhône, Aix-en-Provence, France) within the Chattian (23.03–28.1 Ma) of the late Oligocene^54,101^. †*Thaites* is sister to Parnassiini, and occasionally sister to Luehdorfiini + Zerynthiini^18^. Thus, we constrained the crown age of Parnassiinae with a uniform distribution bounded by a minimum age of 23.03 Ma. The second is †*Doritites bosniaskii*^102^, an exoskeleton and compression fossil from Italy (Tuscany) from the Messinian (5.33–7.25 Ma, late Miocene)^54^. †*Doritites* is sister to *Archon* (Luehdorfiini^18^), in agreement with Carpenter^103^. The crown of Luehdorfiini was thus constrained for divergence time estimation using a uniform distribution bounded with 5.33 Ma. Absolute ages of geological formations were taken from the latest update of the geological time scale.

We used a conservative approach to apply calibration priors with the selected fossil constraints by setting uniform priors bounded with a minimum age equal to the youngest age of the geological formation where each fossil was found. All uniform calibration priors were set with an upper bound equal to the estimated age of angiosperms (150 Ma^104^), which is more than three times older than the oldest Papilionidae fossil. This upper age is intentionally set as ancient to allow exploration of potentially old ages for the clade. Since the fossil record of butterflies is incomplete and biased^105^, caution is needed in using these fossil calibrations (effect shown in burying beetles^106^).

After enforcing the fossil calibrations, we set the following settings and priors: a partitioned dataset (after the best-fitting PartitionFinder scheme) was analysed using the uncorrelated log-normal distribution clock model, with the mean set to a uniform prior between 0 and 1, and an exponential prior (lambda = 0.333) for the standard deviation. The branching process prior was set to a birth–death^107^ process, using the following uniform priors: the birth–death mean growth rate ranged between 0 and 10 with a starting value at 0.1, and the birth–death relative death rate ranged between 0 and 1 (starting value = 0.5). We performed four independent BEAST analyses for 100 million generations, sampled every 10,000th, resulting in 10,000 samples in the posterior distribution, of which the first 2500 samples were discarded as burn-in. All analyses were performed on the CIPRES Science Gateway computer cluster^94^, using BEAGLE^95^. Convergence and performance of each MCMC run were evaluated using Tracer 1.7.1^92^ and the ESS for each parameter (ESS > 200). We combined the four runs using LogCombiner 1.8.4^97^. A maximum-clade credibility (MCC) tree was reconstructed, with median ages and 95% CIs. The BEAST files generated for this study are available in Figshare (see Data availability).

### Estimating ancestral host–plant association

We inferred the temporal evolution of host–plant association up to the ancestral host plant(s) at the root of Papilionidae using three approaches: the ML implementation of the Markov k-state (Mk) model^108^, the ML Dispersal-Extinction-Cladogenesis (DEC) model^109^, and the Bayesian approach in BayesTraits^110^. These approaches require a time-calibrated tree and a matrix of character states (current host–plant preference) for each species in the tree. An extensive bibliographic survey was conducted to obtain primary larval host plants at the family level^5,30,111–113^. The host associations of species were categorized using the following 12 character states: (1) Annonaceae, (2) Apiaceae, (3) Aristolochiaceae, (4) Crassulaceae or Saxifragaceae (core Saxifragales), (5) Fabaceae, (6) Hernandiaceae, (7) Lauraceae; (8) Magnoliaceae, (9) Papaveraceae, (10) Rosaceae, (11) Rutaceae, and (12) Zygophyllaceae. The host–plant matrix of Papilionidae is available in Figshare (see Data availability).

Ancestral states for host–plant association were first reconstructed using the Mk model (one rate for all transitions between states) allowing any host shift to be equally probable. The Mk model does not allow multiple states for a species. The few species that use multiple host families were thus scored with the most frequent host association. The Mk model was performed with Mesquite 3.1 (available at: www.mesquiteproject.org). To estimate the support of any one character state over another, the most likely state was selected according to a decision threshold, such that if the log likelihoods between two states differ by two log-likelihood units, the one with lower likelihood is rejected^108^.

The DEC model was also used to reconstruct ancestral host–plant states^109,114^. As with the Mk model, we assumed that host–plant shifts occurred at equivalent probabilities between plant families and through time, which may not be true given that the host–plant families of Papilionidae did not originate at the same time (e.g. Aristolochiaceae originated ~108.07 Ma [95% CIs: 81.01–132.66 Ma]^115^, and Annonaceae originated ~98.94 Ma [95% CIs: 84.78–113.70 Ma]^115^). We used the estimated molecular ages of the different host–plant groups to constrain our inferences of ancestral host plants a posteriori. We preferred such an approach compared to a more constrained one in which the DEC model is informed with a matrix of host–plant appearances based on their estimated ages by implementing matrices of presence/absence of the character states through time (equivalent to the time-stratified palaeogeographic model, see below for inference of biogeographical history).

Finally, the Bayesian approach implemented in BayesTraits 3.0.1^110^ was performed to provide a cross-validation of ML analyses. This approach automatically detects shifts in rates of evolution for multistate data using rj-MCMC. The number of parameters and priors was set by default. We ran the rj-MCMC for ten million generations and sampled states and parameters every 1000 generations (burn-in of 10,000 generations). We specifically estimated ancestral states at 21 nodes as well as at the root of Papilionidae. For this analysis, we used a set of 100 trees randomly taken from the dating analysis to probe the robustness of our ancestral-state estimation across topological uncertainty.

The results of these inferences determined the host–plant family(ies) that was (were) the most likely ancestral host(s) at the origin of Papilionidae, indicating (1) which plant phylogeny to reconstruct for studying the macroevolution of the arms race, and (2) the evolution of ancestral host–plant association along the phylogeny to identify the tree branches where shifts occurred and test for genome-wide changes.

The Mk and BayesTraits models always inferred with high support (relative probability = 0.915 and 0.789, respectively) that Aristolochiaceae is the ancestral host plant at the crown of Papilionidae. With the unconstrained DEC model, we found that the ancestral host–plant preference for Papilionidae was always composed of Aristolochiaceae, but also included another family (either Fabaceae, Hernandiaceae or Zygophyllaceae, which are only fed upon by *Baronia*, *Lamproptera* and *Hypermnestra*, respectively). As the sister lineage to all other Papilionidae, *Baronia* is the only species that feeds on Fabaceae. More precisely, only one species of Fabaceae is consumed: *Vachellia cochliacantha* (formerly *Acacia cochliacantha*; recent changes in *Acacia* taxonomy^116^). However, *Vachellia* diverged from its sister clade in the Eocene, ~50 Ma, and diversified in the Miocene between 13 and 17 Ma^117^, which substantially postdate the origin of Papilionidae. Therefore, this result suggests that the family Aristolochiaceae represents the most likely candidate as the ancestral host plant of Papilionidae. Hernandiaceae are consumed by *Lamproptera* (occasionally by *Papilio homerus*, *Graphium codrus*, *G. doson* and *G. empedovana*^113^). More precisely, the host plants of *Lamproptera* belong to the genus *Illigera*. This plant genus diverged from its sister genus 48 Ma^115^ and started diversifying 27 Ma^118^. The derived phylogenetic position of *Lamproptera* and the age of its use as a host plant make it very unlikely that Hernandiaceae could constitute the ancestral host plant for Papilionidae. Similarly, the family Zygophyllaceae is consumed by *Hypermnestra*, most specifically it feeds on the genus *Zygophyllum* in Central Asia. The genus *Zygophyllum* is not monophyletic, but Asian *Zygophyllum* appeared 19.6 Ma^119^. Applying the same rationale, we are able to discard Zygophyllaceae as a candidate ancestral host plant for Papilionidae. To further refine our ancestral host–plant estimates, we built a presence–absence matrix of plant families based on clade origins estimated in molecular dating studies. Thereby, the age of the different plants can be used to constrain the inference of ancestral host plants. Under such a constrained model, Aristolochiaceae is always recovered as the most likely ancestral host plant for Papilionidae. It is also interesting that almost all Aristolochiaceae feeders have *Aristolochia* as host plants, and tests to determine which genus of Aristolochiaceae was originally consumed by Papilionidae showed that it was *Aristolochia*.

### Time-calibrated phylogeny of the ancestral host: the Aristolochiaceae

Estimation of ancestral host–plant relationships indicated that the family Aristolochiaceae was the ancestral host for Papilionidae. We refer to Aristolochiaceae in its traditional circumscription including the genera *Asarum*, *Saruma*, *Thottea* and *Aristolochia*. The Angiosperm Phylogeny Group^120^ proposes that Aristolochiaceae also includes the holoparasitic genera *Hydnora* and *Prosopanche* (Hydnoraceae), as well as the monotypic family Lactoridaceae from the Juan Fernandez Islands of Chile (*Lactoris fernandeziana*). The conclusion of Angiosperm Phylogeny Group (APG)^120^ is based on an online survey^121^ rather than on primary data and this is why we disagree with their argumentation as well as the resulting conclusion of APG given available resilient primary molecular phylogenomic data. However, arguments based on morphology and anatomy^122–125^, genetics^49,50,126–129^, molecular divergence time^115,129^, and conservation considerations (Tod Stuessy, personal communication with S.W., July 2019) favour splitting them into four families: Aristolochiaceae (*Aristolochia* and *Thottea*), Asaraceae (*Asarum* and *Saruma*), Hydnoraceae (*Hydnora* and *Prosopanche*), and Lactoridaceae (*Lactoris*), collectively called the perianth-bearing Piperales. Therefore, we extracted and assembled a supermatrix dataset with available data from GenBank for the perianth-bearing Piperales and its sister lineage, the perianth-less Piperales including Saururaceae and Piperaceae (as of May 2017, most of which has been generated by our research group). We obtained four chloroplast genes (*matK*, *rbcl*, *trnL* and *trnL-trnF*) and one nuclear marker (*ITS*) for 247 species of perianth-bearing Piperales (~49% of the total species diversity^130^) and six outgroups from perianth-less Piperales. We could not include the two genera *Hydnora* and *Prosopanche* (Hydnoraceae) because available genetic data do not overlap those of perianth-bearing Piperales^126,128,131,132^. We applied the same analytical procedure that we did for Papilionidae. DNA sequences for each gene were aligned using MAFFT 7.110^84^ with default settings (E-INS-i algorithm and Q-INS-I to take into account secondary structure). Resulting alignments were checked for codon stops and eventually refined by eye with Mesquite 3.1 (available at: www.mesquiteproject.org). The best-fit partitioning schemes and substitution models for phylogenetic analyses were determined with PartitionFinder 2.1.1^85^. All gene alignments were concatenated into a supermatrix; the final dataset is available in Figshare (see Data availability).

Phylogenetic relationships were estimated with Bayesian inference as implemented in MrBayes 3.2.6^90^. Rather than using a single substitution model per molecular partition, we sampled across the entire substitution-model space^91^ using rj-MCMC. Two independent analyses with one cold chain and seven heated chains, each was run for 50 million generations, sampled every 5000 generations. Convergence and performance of Bayesian runs were evaluated using Tracer 1.7.1^92^ and the ESS, ADSF and PSRF criteria. Once convergence was achieved, a 50% majority-rule consensus tree was built after discarding 25% of the sampled trees as burn-in.

Bayesian relaxed-clock methods were used that accounted for rate variation across lineages^96^. MCMC analyses implemented in BEAST 1.8.4^97^ were employed to approximate the posterior distribution of rates and divergences times and infer their CIs. Estimation of divergence times relied on constraining clade ages through fossil calibrations. Three unambiguous fossils from perianth-bearing Piperales (Aristolochiaceae *sensu lato*), and one corresponding to the family Saururaceae were used. First, we relied on the fossil record of the monotypic family Lactoridaceae (*L. fernandeziana*)^126,129^, a shrub endemic to the cloud forest of the Juan Fernández Islands archipelago of Chile. The oldest pollen fossil for the group is †*Lactoripollenites africanus*^133,134^ from the Turonian/Campanian (72.1–89.8 Ma) of the Orange Basin in South Africa. This fossil confers a minimum age of 72.1 Ma for the stem node of *L. fernandeziana*. Second, the oldest and only pollen record of the Aristolochiaceae was recently described from Late Cretaceous sediments of Siberia: †*Aristolochiacidites viluiensis*^56^ from the Timerdyakh Formation of the latest Campanian to earliest Maastrichtian (66–72.1 Ma) in the Vilui Basin (Russia). Because inaperturate pollen grains in combination with this unique exine configuration and fitting size can be observed in extant members of Aristolochiaceae, this fossil provides a minimum age of 66 Ma for the family. The third fossil belongs to the genus *Aristolochia* and described as †*Aristolochia austriaca*^57^ from the Pannonian (late Miocene) in the Hollabrunn–Mistelbach Formation (Austria). Based on a thorough morphological leaf comparison, this fossil is assigned to a species group including *Aristolochia baetica* and *Aristolochia rotunda*, which then confers a minimum age of 7.25 Ma for the clade. Finally, we used the fossil †*Saururus tuckerae*^135^ from the Princeton Chert of Princeton in British Columbia (Canada), which is part of the Princeton Group, Allenby Formation dated with stable isotopes to the middle Eocene^136^. This fossil has been phylogenetically placed as sister to extant *Saururus* species^136^, hence providing a minimum age of 44.3 Ma for the stem node of *Saururus*. Absolute ages of geological formations were taken from the latest update of the geological time scale.

We set the following settings and priors: a partitioned dataset (after the best-fitting PartitionFinder scheme) was analysed using the uncorrelated log-normal distribution clock model, with the mean set to a uniform prior between 0 and 1, and an exponential prior (lambda = 0.333) for the standard deviation. The branching process prior was set to a birth–death^107^ process, using the following uniform priors: the birth–death mean growth rate ranged between 0 and 10 with a starting value at 0.1, and the birth–death relative death rate ranged between 0 and 1 (starting value = 0.5). We performed four independent BEAST analyses for 100 million generations, sampled every 10,000th, resulting in 10,000 samples in the posterior distribution of which the first 2500 samples were discarded as burn-in. All analyses were performed on the CIPRES Science Gateway computer cluster^94^, using BEAGLE^95^. Convergence and performance of each MCMC run were evaluated using Tracer 1.7.1^92^ and the ESS for each parameter. We combined the four runs using LogCombiner 1.8.4^97^. The MCC tree was reconstructed with median age and 95% CI. The BEAST files generated for this study are available in Figshare (see Data availability).

### Dual biogeographic history of Papilionidae and Aristolochiaceae

We estimated the ancestral area of origin and geographic range evolution for both clades using the ML approach of DEC model^109^ as implemented in the C++ version^137,138^ that is available at: https://github.com/champost/DECX. To infer the biogeographic history of a clade, DEC requires a time-calibrated tree, the current distribution of each species for a set of geographic areas, and a time-stratified geographic model that is represented by connectivity matrices for specified time intervals spanning the entire evolutionary history of the group.

The geographic distribution for each species in Papilionidae^30,112,113^ and Aristolochiaceae was categorized as present or absent in each of the following areas: (1) WN, (2) East Nearctic, (3) CA, (4) South America, (5) West Palaearctic, (6) EP, (7) Madagascar, (8) Indonesia and Wallacea, (9) India, (10) Africa and (11) Australasia. The resulting matrices of species distribution for the two groups are available in Figshare (see Data availability).

A time-stratified geographic model was built using connectivity matrices that take into account palaeogeographic changes through time, with time slices indicating the possibility or not for a species to access a new area^138^. Based on palaeogeographical reconstructions^139–141^, we created a connectivity matrix for each geological epoch that represented a period bounded by major changes in tectonic and climatic conditions thought to have affected the distribution of organisms. The following geological epochs were selected: (1) 0–5.33 Ma (Pliocene to present), (2) 5.33–23.03 Ma (Miocene), (3) 23.03–33.9 Ma (Oligocene), (4) 33.9–56 Ma (Eocene) and (5) 56 Ma to the origin of the clade (Palaeocene to Late Cretaceous). For each of these five time intervals, we specified constraints on area connectivity by coding 0 if any two areas are not connected or 1 if they are connected in a given time interval. We assumed a conservative dispersal matrix with equal dispersal rates between areas through time^142^.

### Impact of host–plant shifts on swallowtail diversification

We tested the effect of host–plant association on diversification by estimating speciation and extinction rates with five methods to cross-test hypotheses and corroborate results. Analyses were performed on 100 dated trees randomly sampled from the Bayesian dating analyses to take into account the uncertainty in age estimates. We used the following approaches: (1) ML-based trait-dependent diversification^143,144^; (2) ML-based time-dependent diversification^145^; (3) Bayesian analysis of macroevolutionary mixture^146^; (4) Bayesian branch-specific diversification rates^147^; and (5) Bayesian episodic birth–death model^148^. It is worth mentioning that each method differs at several points in their estimation of speciation and extinction rates. For instance, trait-dependent birth–death models estimate constant speciation and extinction rates^144^, whereas time-dependent birth–death models estimate clade-specific speciation and extinction rates and their variation through time^145,147^. Therefore, we expect some differences in the values of estimated diversification rates that are inherent to each approach. Our diversification analyses should be seen as complementary to the inferred diversification trend rather than corroborating the values and magnitude of speciation and extinction rates.

First, we computed the probability of obtaining a clade as large as size *n*, given the crown age of origin, the overall net diversification rate of the family, and an extinction rate as a fraction of speciation rate following the approach in Condamine et al.^34^ relying on the method of moments^149^. We used the R-package *LASER* 2.3^150^ to estimate the net diversification rates of Papilionidae and six clades shifting to new host plants with the *bd.ms* function (providing crown age and total species diversity). Then, we used the *crown.limits* function to estimate the mean expected clade size for each clade shifting to new host plants given clades’ crown age and overall net diversification rates, and we finally computed the probability to observe such clade size using the *crown.p* function. All rate estimates were calculated with three *ε* values (*ε* = 0/0.5/0.9), knowing that the extinction rate in swallowtails is usually low^34^ (supported by the results of this study).

Second, we relied on the state-dependent speciation and extinction (SSE) model, in which speciation and extinction rates are associated with phenotypic evolution of a trait along a phylogeny^143^. In particular, we used the MuSSE^144^ implemented in the R-package *diversitree* 0.9-10^151^, which allows multiple character states to be studied. Larval host–plant data were taken from previous works^5,18,30,34,112,113,152^. The following ten host–plant character states and corresponding ratios of sampled species in the tree of all known species for each character (sampling fractions) were used: 1 = Aristolochiaceae (110/152), 2 = Annonaceae (69/138), 3 = Lauraceae (33/39), 4 = Apiaceae (9/10), 5 = Rutaceae (119/163), 6 = Crassulaceae (19/19), 7 = Papaveraceae (44/44), 8 = Fabaceae (1/1), 9 = Zygophyllaceae (2/2), and 10 = Magnoliaceae (2/2). Data at a lower taxonomic level than plant family were not used because of the large number of multiple associations exhibited by genera that could alter the phylogenetic signal. We assigned a single state to each species by selecting the food plant with the maximum number of collections for each species. We did not employ multiple states per species, which represents a lesser problem because (1) few swallowtail species feed on multiple plant families, (2) current shared-state models can only model two states, and (3) the addition of multi-plant states to the MuSSE analysis would have greatly increased the number of parameters. We performed both ML and Bayesian MCMC analyses (10,000 steps) performed using an exponential (1/(2 × net diversification rate)) prior with starting parameter values obtained from the best-fitting ML model and resulting speciation, extinction and transition rates. After a burn-in of 500 steps, we estimated posterior density distribution for speciation, extinction and transition rates. There have been concerns about the power of SSE models to infer diversification dynamics from a distribution of species traits^153–155^, hence other birth–death models were used to corroborate the results obtained with SSE models.

Third, to provide an independent assessment of the relationship between diversification rates and host specificity, we used the ML approach of Morlon et al.^145^ implemented in the R-package *RPANDA* 1.3^156^. This is a birth–death method in which speciation and/or extinction rates may change continuously through time. This method has the advantage of not assuming a constant extinction rate over time (unlike BAMM^146^), and allows clades to have declining diversity since extinction can exceed speciation, meaning that diversification rates can be negative^145^. For each clade that shifted to a new host family, we designed and fitted six diversification models: (1) a Yule model, where speciation is constant and extinction is null; (2) a constant birth–death model, where speciation and extinction rates are constant; (3) a variable speciation rate model without extinction; (4) a variable speciation rate model with constant extinction; (5) a rate-constant speciation and variable extinction rate model; and (6) a model in which both speciation and extinction rates vary. Models were compared by computing the ML estimate of each model and the resulting Akaike information criterion corrected by sample size. We then plotted rates through time with the best-fit model for each clade, and the rates for the family as a whole for comparison purpose.

Fourth, we performed models that allow diversification rates to vary among clades across the whole phylogeny. BAMM 2.5^146,157^ was used to explore for differential diversification dynamic regimes among clades differing in their host–plant feeding. BAMM can automatically detect rate shifts and sample distinct evolutionary dynamics that explain the diversification dynamics of a clade without a priori hypotheses on how many and where these shifts might occur. Evolutionary dynamics can involve time-variable diversification rates; in BAMM, speciation is allowed to vary exponentially through time while extinction is maintained constant: subclades in a tree may diversify faster (or slower) than others. This Bayesian approach can be useful in detecting shifts of diversification potentially associated with key innovations^157^. BAMM analyses were run with four MCMC for 20 million generations, sampling every 20,000th and with three different values (1, 5 and 10; Supplementary Table 3) of the compound Poisson prior (CPP) to ensure the posterior is independent of the prior^158^. We accounted for non-random incomplete taxon sampling using the implemented analytical correction; we set a sampling fraction per genus based on the known species diversity of each genus. Mixing and convergence among runs (ESS > 200 after 15% burn-in) were assessed with the R-package *BAMMtools* 2.1^159^ to estimate (1) the mean global rates of diversification through time, (2) the estimated number of rate shifts evaluating alternative diversification models comparing priors and posterior probabilities and (3) the clade-specific rates through time when a distinct macroevolutionary regime is identified.

Fifth, BAMM has been criticized for incorrectly modelling rate shifts on extinct lineages, that is, unobserved (extinct or non-sampled) lineages inherit the ancestral diversification process and cannot experience subsequent diversification-rate shifts^158,160^. To solve this, we used a Bayesian approach implemented in RevBayes 1.0.10^161^ that models rate shifts consistently on extinct lineages by using the SSE framework^147,158^. Although there is no information of rate shifts for unobserved/extinct lineages in a phylogeny including extant species only, these types of events must be accounted for in computing the likelihood. The number of rate categories is fixed in the analysis but RevBayes allows any number to be specified, thus allowing direct comparison of different macroevolutionary regimes.

Finally, we evaluated the impact of abrupt changes in diversification using the Bayesian episodic birth–death model of CoMET^148^ implemented in the R-package *TESS* 2.1^162^. These models allow detection of discrete changes in speciation and extinction rates concurrently affecting all lineages in a tree, and estimate changes in diversification rates at discrete points in time, but can also infer mass extinction events (sampling events in which the extant diversity is reduced by a fraction^163^). Speciation and extinction rates can change at those points, but remain constant within time intervals. In addition, TESS uses independent CPPs to simultaneously detect mass extinction events and discrete changes in speciation and extinction rates, while TreePar estimates the magnitude and timing of speciation and extinction changes independently to the occurrence of mass extinctions (i.e. the three parameters cannot be estimated simultaneously due to parameter identifiability issues^163^). We performed two independent analyses allowing and disallowing mass extinction events. Bayes factor comparisons were used to assess the model fit between models with varying number and time of changes in speciation/extinction rates and mass extinctions.

### Detecting genome-wide adaptations during host–plant shifts

We analysed genomic sequence data in swallowtail butterflies that have independently shifted to new ecological (biological) traits. Similar approaches have been conducted on mammals^164,165^ and birds^166^, but have been rarely implemented on arthropod groups over such a long geological time scale. Here, we estimated swallowtail molecular evolution with whole-genome data and compared selection regimes on protein-coding genes along independent branches with or without host–plant shift and/or environmental shift.

For these analyses, we studied 45 whole genomes^46^ covering all 32 genera of the family Papilionidae: 41 of which were previously generated by our research group added to four genomes already available^70–72^. In summary, raw reads (Sequence Read Archive: SRR8954507-SRR8954549) were cleaned using Trimmomatic 0.33^167^, and assembled into contigs and scaffolds with SOAPdenovo-63mer 2.04^168^ to obtain whole-genome assemblies (30× average read depth^46^). All coding DNA sequences (CDS) were retrieved from the high-quality annotated genome of *P. xuthus*^71^. To annotate the sequences of all our genomes, a BLAST search using all available CDS of *P. xuthus* was performed at the amino-acid level (using tblastn). For each species, the recovered genes were aligned one by one with *P. xuthus* using TranslatorX^169^. This method performs alignment at the amino-acid level and preserves the open reading frame. All sites showing intraspecific variation were set to *N*, to conservatively avoid false informative sites. Any contamination was removed using CroCo 0.1^170^ and orthologous proteins were identified with OrthoFinder 2.2.0^171^. Finally, CDS alignments were strongly cleaned from misaligned sequences (gene by gene) using HMMCleaner 1.8^172^. A last cleaning step was performed using trimAl 1.2rev59^173^, which is designed to trim alignments for large-scale phylogenomic analyses. The resulting dataset comprised 6621 genes in at least four sampled species (median of 32% of missing data), which was used to reconstruct a robust phylogenomic tree of Papilionidae^46^ (Supplementary Fig. 18).

We used this genomic dataset of 45 species representing all genera in which the resulting genus-level swallowtail phylogenomic tree^46^ accurately represents the evolutionary associations with host plants as estimated using the ancestral-state analyses applied to the species-level phylogeny^34^ (Fig. 1 and Supplementary Figs. 4 and 5). We thus transferred the inference of ancestral host–plant shifts on the phylogenomic tree and selected the branches representing a host–plant shift and/or a shift of climate preference (in general from tropical to temperate conditions; Supplementary Fig. 10). We also selected branches with no change as negative controls^73^. As a result, 14 branches are selected to measure the impact of a host–plant shift and 14 branches are selected as controls (Supplementary Fig. 18). Within these 14 branches with an ecological change, nine branches represent host–plant shifts only, and five branches correspond to shifts in both host plant and environment (from tropical to temperate conditions). To test the impact of these different changes on the genomes, two datasets were created, *Dataset 1* and *2*. Given the low quality of the genomes of *Allancastria cerisyi* and *Parnassius imperator*, these two genomes were discarded for the downstream analyses. We first selected the genes from the 6621-gene dataset for each focal branch using three criteria: (1) the dataset is composed only of orthologous protein-coding genes (OrthoFinder 2.2^171^), (2) the species needed to accurately define the branch were available (i.e. crown node of the clade) and (3) for each branch, one species per tribe was available, and therefore include a different number of genes per branch. Thus, for Dataset 1, only the genes containing sequences for the species needed to generate all focal branches were selected. This stringent selection leads to Dataset 1, comprising only 520 genes but the same genes for all branches (no missing genes). For Dataset 2, the genes were selected for each branch independently (i.e. for a given branch, a gene was selected if the sequence needed to generate that branch was present). This second selection leads to 1439 genes per branch on average among a total of 1533 genes, which were selected at least once for one branch. The genomic dataset is available in Figshare (see Data availability).

We studied the ratio (*ω*) of dN/dS to find genes under positive selection^76,174^. The dN/dS ratio is traditionally used to estimate selective pressure from protein-coding sequences. If host–plant shifts have no effect on the selection of a given gene, we expect a dN/dS = 1 and the selective regime is considered neutral. However, if host–plant shifts result in positive selection on coding genes, the ratio increases such that dN/dS > 1. Finally, it is possible that host–plant shifts lead to purifying selection, thus reducing the number of dN and resulting in dN/dS < 1. Here we focused on the adaptation of Papilionidae to host–plant shifts, that is, outgroups are not studied. We tested if branches representing inferred host–plant shifts along the phylogeny of swallowtails have more genes with dN/dS > 1 than lineages that did not have an inferred shift. The *branch-site* models allow *ω* to vary both among sites in the protein and across branches on the tree and aim to detect positive selection affecting a few sites along particular lineages. The approach described by Zhang et al.^175^ was chosen to determine genome-wide selection regimes as performed with two ML models: (1) a null model assuming two site classes, one with dN/dS < 1 and one with dN/dS = 1 (model = 2, NSsites = 2, fix_omega = 1, omega = 1) and (2) an alternative model adding a third site class with dN/dS > 1 (model = 2, NSsites = 2, fix_omega = 0, omega = 1.5). The fit for including positive selection is tested using a likelihood ratio test comparing the null model with the alternative model with one degree of freedom^76,176^. If the alternative model is better suited to host-shift branches, it is more likely the gene was under positive selection during the host–plant shifts. For each gene and for each branch, both the null and alternative models using CodeML were implemented in PAML 4^177^ with a fixed topology (as inferred with the phylogenomic dataset^46^) and the nucleotide alignment of each gene. To test the robustness of the estimations, we used a false discovery rate test to control false positives^178^. Finally, for each branch, we reported the number of genes under positive selection (i.e. for which the alternative model including the site class with dN/dS > 1 have a better likelihood) on the total gene number. The proportion of genes under positive selection was compared with associated control branches for branches representing host–plant shifts, environmental shifts or both plant and environmental shifts using the non-parametric, Wilcoxon rank-sum test^179^.

### Sensitivity analyses

We performed several control analyses to ensure that the signal of more genes under positive selection in host–plant shifts branches is not artefactual.

First, it has been shown that the choice of the tree is an important factor for the branch-site analysis of positive selection^180^. Indeed, constraining the topology for a given gene may lead to overestimating the number of substitution events for the constrained branches^78^ and so could lead to overestimating the dN/dS ratio. Estimating dN/dS over thousands of gene trees would make the branch comparison not equal between control and test branches. Indeed, in a given gene tree it is likely and expected that the species topology is not always recovered, which results in a different number of branches compared to the species tree. For instance, the host–plant shift to Annonaceae might disappear in certain proportions of genes. We thus decided to estimate dN/dS on a fixed species tree topology for all genes to be sure to be able to measure this ratio for each gene that must be present in the topology for the focal branches. However, given that this issue can lead to a bias in our analysis, we decided to compute the number of gene trees that did not recover the branches of interest. We then checked whether the branches leading to a host–plant shift were more often unrecovered than the control branches without shift. Overall the control branches were less often recovered than host–plant shift branches (*P* = 0.030, Wilcoxon rank-sum test; data presented in Supplementary Table 4), which suggests that if gene tree/species tree discordance leads to an overestimation of positive selection, then this overestimation is higher for control branches than for host–plant shift branches. Finally, we filtered out the gene trees for which the focal branches were recovered in agreement with the species tree and used these genes to re-estimate the proportion of genes under positive selection among this new set of genes. We found that the *P* value remains significant (*P* = 0.0444, Wilcoxon rank-sum test; more genes during host–plant shifts than along control branches, Supplementary Table 2). Then, we specifically focused on missing data and GC-content variation among genes known to bias dN/dS estimations. Missing data are prone to introducing misaligned regions that could create false positives in branch-site likelihood method for detecting positive selection^181–183^. Variations in GC content are known to impact the estimation of dN/dS mainly through the process of GC-biased gene conversion (gBGC^184–186^).

The number of missing data (‘*N*’ and ‘-’) sites and GC content at the third codon position (GC3) were computed using a home-made C++ program created with BIO++ library^187^. Mean GC content and missing data were calculated per gene and for each branch. For a given branch, mean GC3 and missing data were computed for the species of a clade for which the branch is the root. All statistics and graphical representations were performed using the R-packages *tidyverse*^188^ and *cowplot*^189^. We found that genes under positive selection (PS_genes_, *n*_Dataset 1_ = 142, *n*_Dataset 2_ = 407) have significantly more missing data and GC3 than genes not under positive selection (NS_genes_, *n*_Dataset 1_ = 378, *n*_Dataset 2_ = 1126; *P* = 0.001/0.02 for the two datasets, respectively, Mann–Whitney test; Supplementary Fig. 20). This result confirms that branch-site likelihood methods for detecting positive selection are sensitive to missing data, probably because of misaligned sites^181,182^, and that GC content that may be influenced by gBGC^184,185^.

Missing data were, however, heterogeneously distributed among species, ranging from <1% in *P. xuthus* to 45% in *Hypermnestra helios* (Supplementary Fig. 21). The difference in missing data between branches with (*n* = 14, mean missing _Dataset 1_ = 13.4%, mean missing_Dataset 2_ = 14.1%) or without host–plant shifts (*n* = 14, mean missing_Dataset 1_ = 12.8%, mean missing_Dataset 2_ = 12.7%) is not significant (*P* = 0.83/1.00 for the two datasets, respectively, Mann–Whitney test; Supplementary Fig. 22). In addition, there is no correlation between the number of genes under positive selection and the amount of missing data (*P* = 0.33/0.20 for the two datasets, respectively, Spearman’s correlation test; Supplementary Fig. 23). For GC3, we also found variation between species ranging from 37% in *Parnassius smintheus* to 44% in *Papilio antimachus* (Supplementary Fig. 24). Similarly to missing data, we found no significant difference between plant-shift and no plant-shift branches (*P* = 0.63/0.63 for the two datasets, Mann–Whitney test; Supplementary Fig. 25) and there is no correlation between the number of genes under positive selection and GC3 (*P* = 0.20/0.1362 for the two datasets, respectively, Spearman’s correlation test; Supplementary Fig. 26).

Despite the known fact that false positives can increase with the amount of missing data, our control analyses indicate that variations in missing data and GC content do not drive the signal that more genes are under positive selection in branches that have undergone a host–plant shift. Additionally to these controls, we checked by eyes all the gene alignments at the amino-acid level for genes under positive selection in branches with and without host–plant shifts using SeaView 4^190^. Misaligned regions, which could lead to biased dN/dS ratios^191^, were not significantly more detected for genes under positive selection in branches with host–plant shifts. In some cases, we found ourselves in complicated situations to discriminate between false- and true-positive selected genes.

Overall, given our alignment checks and sensitivity analyses, we do not see any reason for biased dN/dS ratios in genes along branches with or without host–plant shifts. False-positive and false-negative genes can be present in the two categories of branches, but, in any cases, the general pattern observed is likely to remain conserved.

### Gene ontology

To annotate proteins of our alignment, we used the two different approaches implemented in PANTHER 14^192^ (available at: http://pantherdb.org/) and EggNOG 5.0^193,194^ (available at: http://eggnog5.embl.de/#/app/home). We used the HMM Scoring tool to assign PANTHER family (library version 14.1^192^) to the protein of *P. xuthus* (assembly Pxut_1.0); similar results were obtained using another high-quality annotated genome (from *H. melpomene*) as reference (assembly ASM31383v2). We performed the statistical overrepresentation test implemented on the PANTHER online website, relying on the GO categories in the PANTHER GO-Slim annotation dataset including Molecular function, Biological process and Cellular component. First, we tested if positively selected genes have over- or under-represented functional GO categories as compared to the whole set of genes (option “PANTHER Generic Mapping”). Second, we tested if positively selected genes involving a host–plant shift along the 14 branches have over- or under-represented functional categories. These statistical comparisons were performed with the Fisher’s exact test using the false discovery rate correction to control for false positives. Independently, we used the eggNOG-mapper v2^193^ (https://github.com/eggnogdb/eggnog-mapper) and the associated Lepidoptera database (LepNOG, including the genomes of *Bombyx mori*, *Danaus plexippus* and *Heliconius melpomene*^194^) to annotate the proteins of our dataset. EggNOG uses precomputed orthologous groups and phylogenies from the database to transfer functional information from fine-grained orthologs only. We used the diamond method as recommended^193^. Finally, we reported the GO families inferred for the proteins of the Dataset 2.

### Reporting summary

Further information on research design is available in the Nature Research Reporting Summary linked to this article.

## Supplementary information

Supplementary Information

Peer Review File

Reporting Summary

Description of Additional Supplementary Files

Supplementary Data 1

Supplementary Data 2

## Data Availability

Supermatrix datasets (for phylogenetic analyses), phylogenetic trees, host–plant preferences, species geographic distributions, and gene alignments (for dN/dS analyses) that are necessary for repeating the analyses described here have been made available through the Figshare digital data repository (10.6084/m9.figshare.12278402). Source data are provided with this paper.

## Source data

Source Data

## Code availability

Bioinformatic scripts used to perform the analyses described here are available through the Figshare digital data repository (10.6084/m9.figshare.12278402).
