# Supplementary material for: Genome-wide macroevolutionary signatures of key innovations in butterflies colonizing new host plants
Source: Nat Commun. 2021 Jan 13;12:354. doi: 10.1038/s41467-020-20507-3 (PMC7806994; doi:10.1038/s41467-020-20507-3)
Supplement: Supplementary file 1 — Supplementary Information [file 41467_2020_20507_MOESM1_ESM.pdf]

Supplementary figures and tables

for

Genome-wide macroevolutionary signatures of key innovations in butterflies colonizing new host plants

Rémi Allio[1]*, Benoit Nabholz[1], Stefan Wanke[2], Guillaume Chomicki[3], Oscar A. Pérez-Escobar[4], Adam M. Cotton[5], Anne-Laure Clamens[6], Gaël J. Kergoat[6], Felix A.H. Sperling[7] & Fabien L. Condamine[1,7]*

Affiliations

[1]Institut des Sciences de l'Evolution de Montpellier (Université de Montpellier | CNRS | IRD | EPHE), Place Eugène Bataillon, 34095 Montpellier, France. [2]Institut für Botanik, Technische Universität Dresden, Zellescher Weg 20b, 01062, Dresden, Germany. [3]Department of Bioscience, Durham University, Stockton Rd, Durham DH1 3LE, UK. [4]Royal Botanic Gardens, Kew, TW9 3AB, Surrey, UK. [5]86/2 Moo 5, Tambon Nong Kwai, Hang Dong, Chiang Mai, Thailand. [6]CBGP, INRAE, CIRAD, IRD, Montpellier SupAgro, Univ. Montpellier, Montpellier, France. [7]University of Alberta, Department of Biological Sciences, Edmonton T6G 2E9, AB, Canada.

Correspondence

Rémi Allio: rem.allio@yahoo.fr

Fabien L. Condamine: fabien.condamine@gmail.com

Supplementary figure 1. Phylogenetic relationships of 408 swallowtail butterfly species (Papilionidae). Left phylogeny is inferred with the maximum-likelihood approach implemented with IQ-TREE, and right phylogeny is inferred with the Bayesian approach implemented with MrBayes. Both phylogenies show similar relationships except for the placement of the genus *Teinopalpus*, found as sister to Papilionini + Troidini with IQ-TREE and sister to *Meandrusa* (Papilionini) with MrBayes. Node support is indicated by ultrafast bootstrap and posterior probabilities on the maximum-likelihood and Bayesian phylogenies, respectively, with values of 95% and 0.95 considered as indicative of strong node support. Pictures of butterflies made by Fabien Condamine.

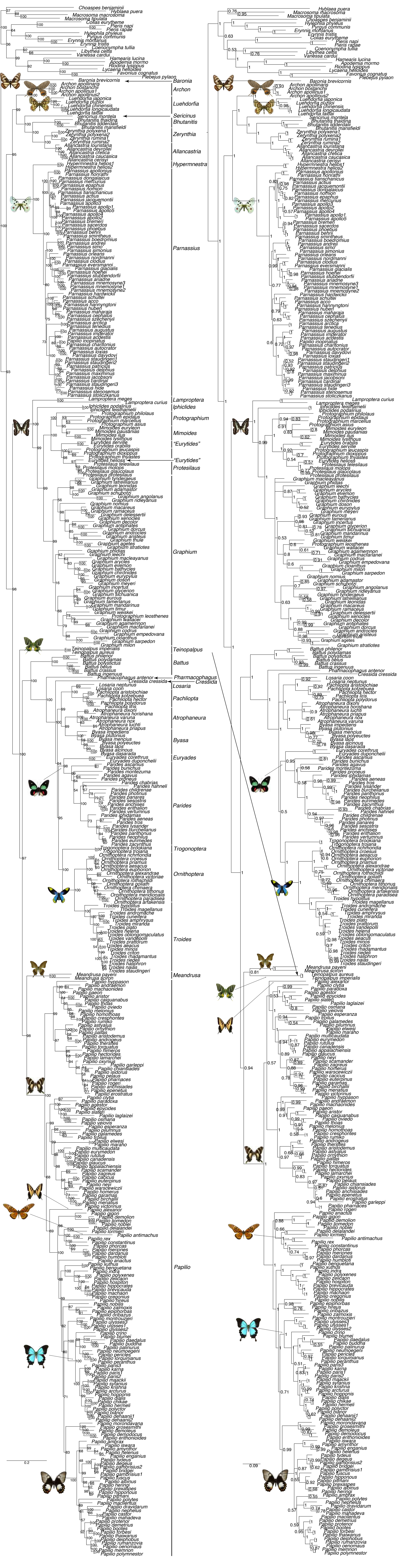

Baronia
Archon
Luehdorfia
Sericinus
Bhutanitis
Zerynthia
Allancastria
Hypermnestra
Parnassius
Lamproptera
Iphiclides
Protographium
Mimoides
"Eurytides"
"Eurytides"
Protesilaus
Graphium
Teinopalpus
Battus
Pharmacophagus
Cressida
Losaria
Pachliopta
Atrophaneura
Byasa
Euryades
Parides
Trogonoptera
Ornithoptera
Troides
Meandrusa
Papilio
0.2
0.09

Supplementary figure 2. Node support (ultrafast bootstrap) of the maximum-likelihood phylogeny. The histogram shows the distribution of node support for all Papilionidae, and indicates a high overall tree resolution with ~80% of nodes having ultrafast bootstrap values ≥ 95%.

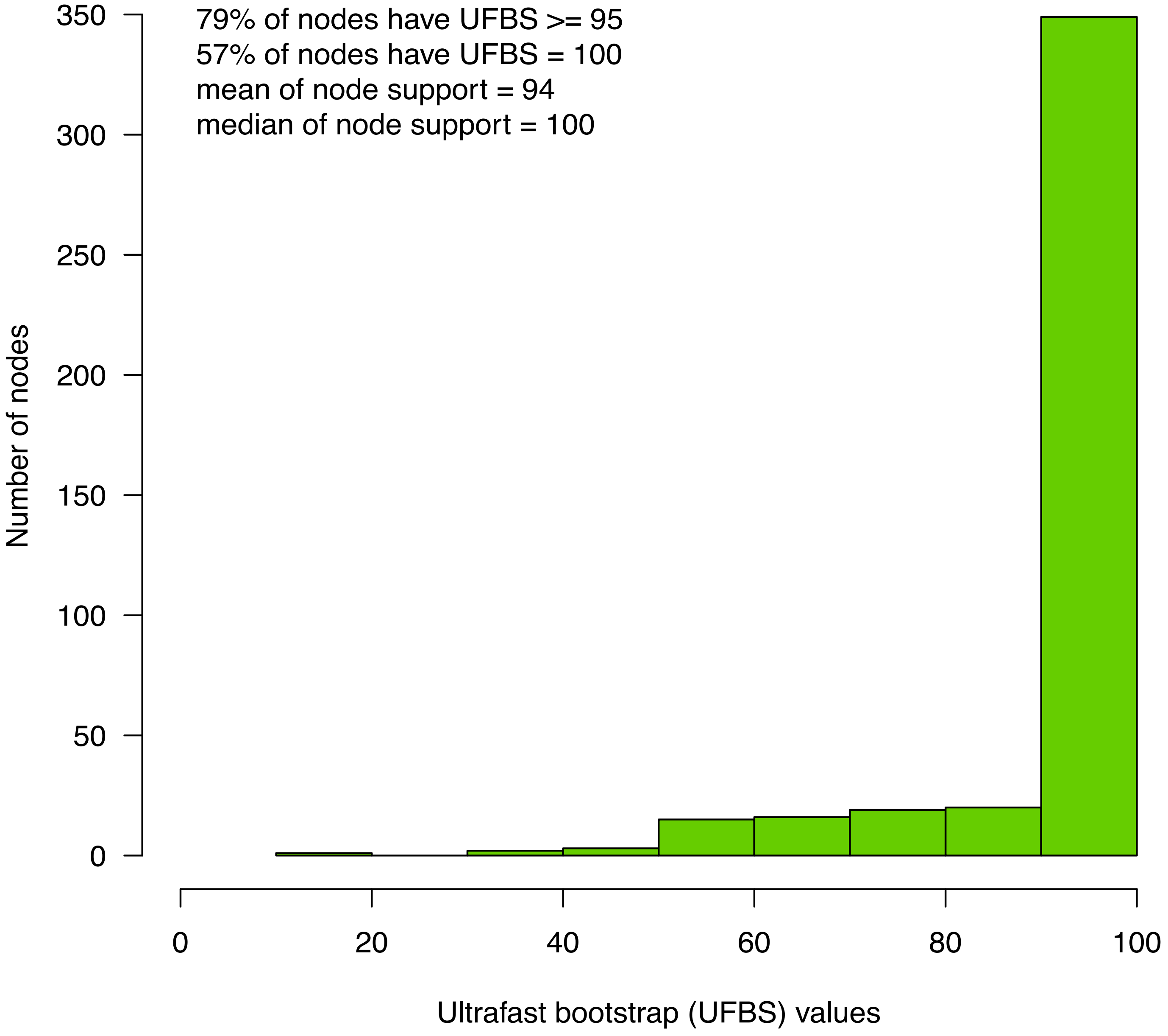

79% of nodes have UFBS >= 95
57% of nodes have UFBS = 100
mean of node support = 94
median of node support = 100
Number of nodes
0
50
100
150
200
250
300
350
0
20
40
60
80
100
Ultrafast bootstrap (UFBS) values

Supplementary figure 3. Bayesian estimates of divergence times for swallowtail butterflies. The first inference was performed with exponential priors on fossil calibrations, while the second inference was carried out with uniform priors. The analysis based on exponential priors estimated a crown age for the family at 55.4 Ma (95% CI: 47.8-71.0 Ma), while the analysis based on uniform priors estimated the origin at 67.2 Ma (95% CI: 47.8-112 Ma).

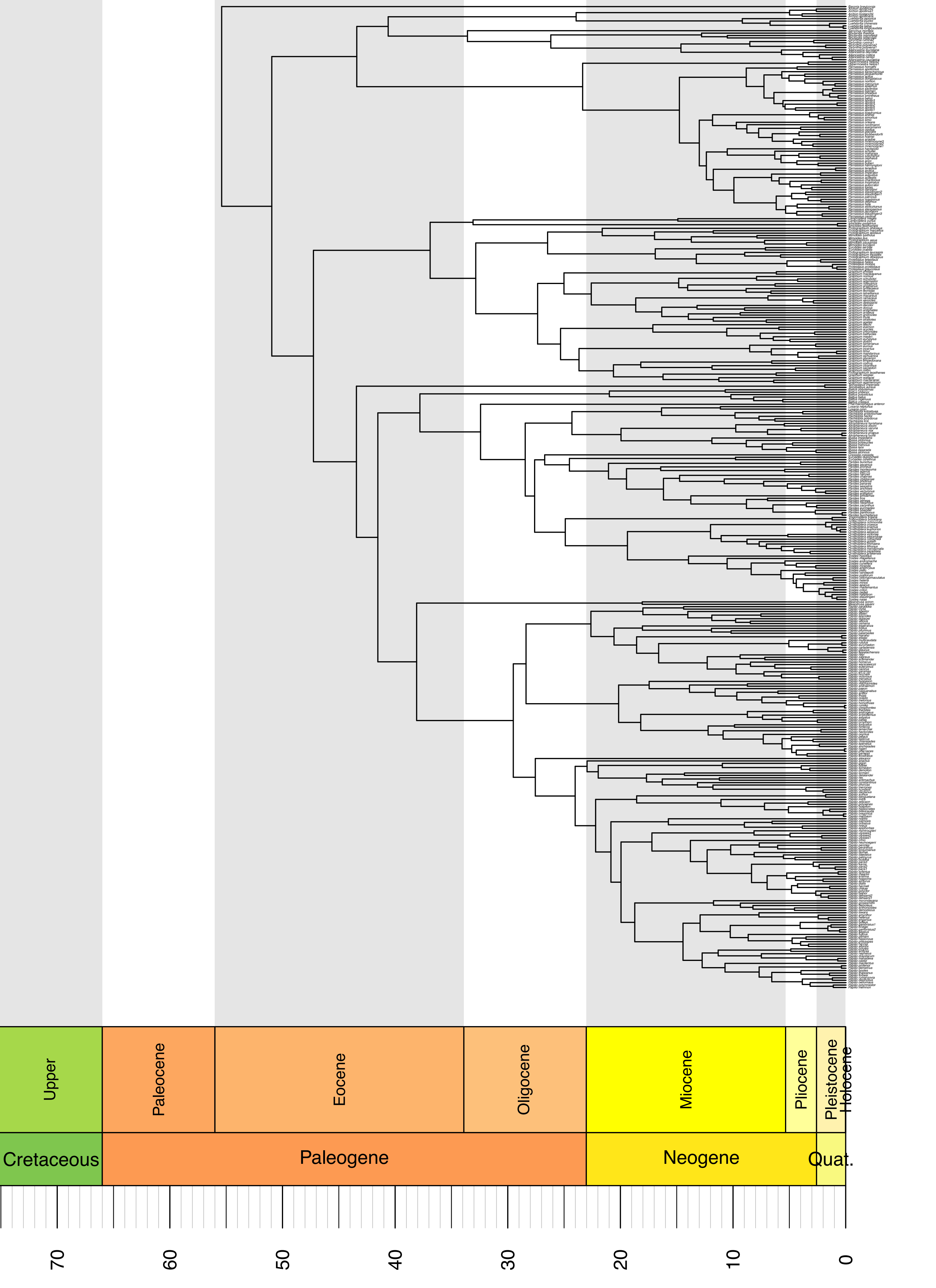

Upper
Paleocene
Eocene
Oligocene
Miocene
Pliocene
Pleistocene
Holocene
Cretaceous
Paleogene
Neogene
Quat.
70
60
50
40
30
20
10
0

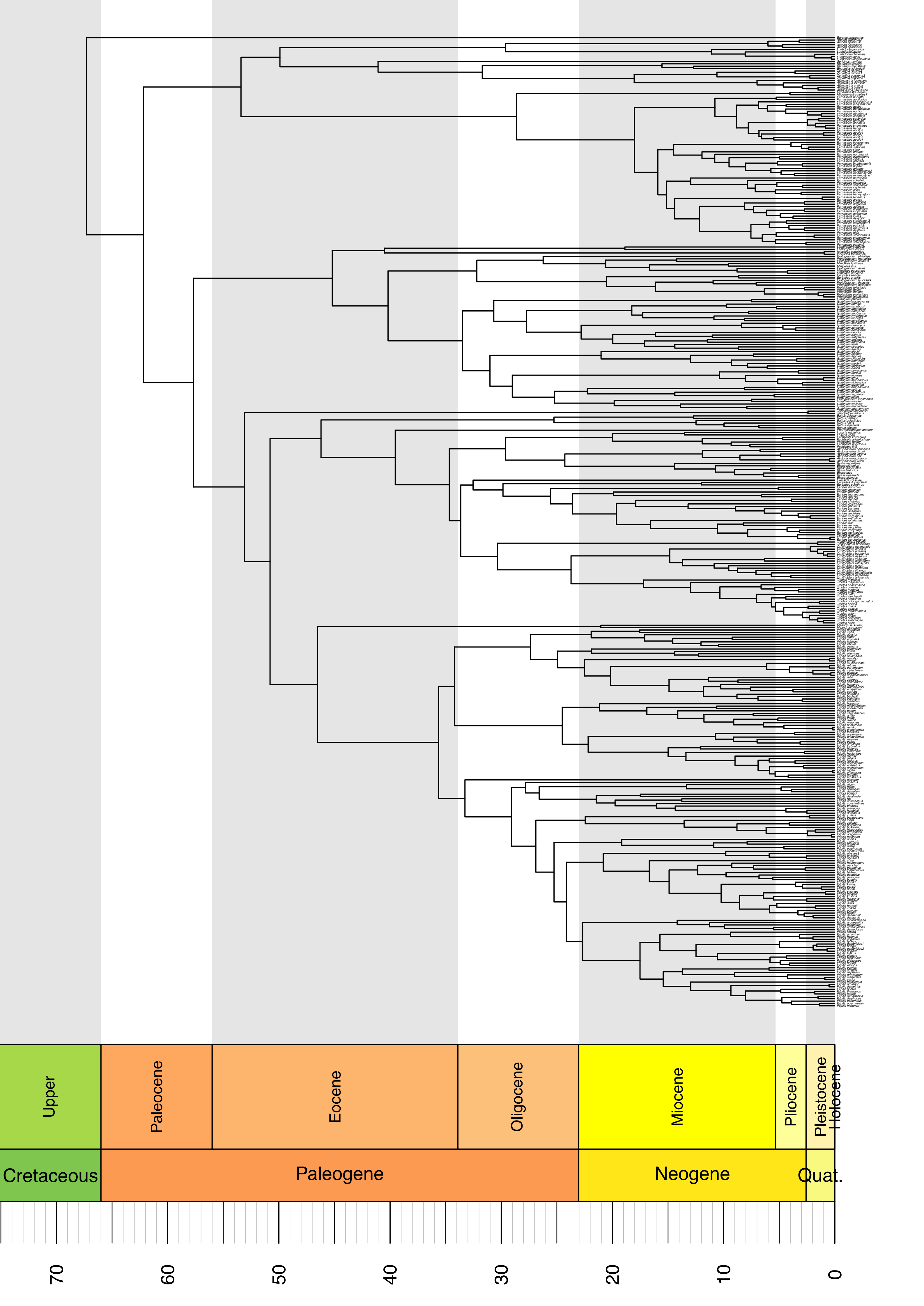
Upper
Paleocene
Eocene
Oligocene
Miocene
Pliocene
Pleistocene
Holocene
Cretaceous
Paleogene
Neogene
Quat.
70
60
50
40
30
20
10
0

Supplementary figure 4. Estimation of ancestral host-plant preferences for the two molecular dated trees with the Dispersal-Extinction-Cladogenesis (DEC) model. The results show that the family Aristolochiaceae is recovered as the ancestral feeding habit of the Papilionidae. K = Cretaceous, Pl = Pliocene, P = Pleistocene. Pictures of butterflies made by Fabien Condamine.

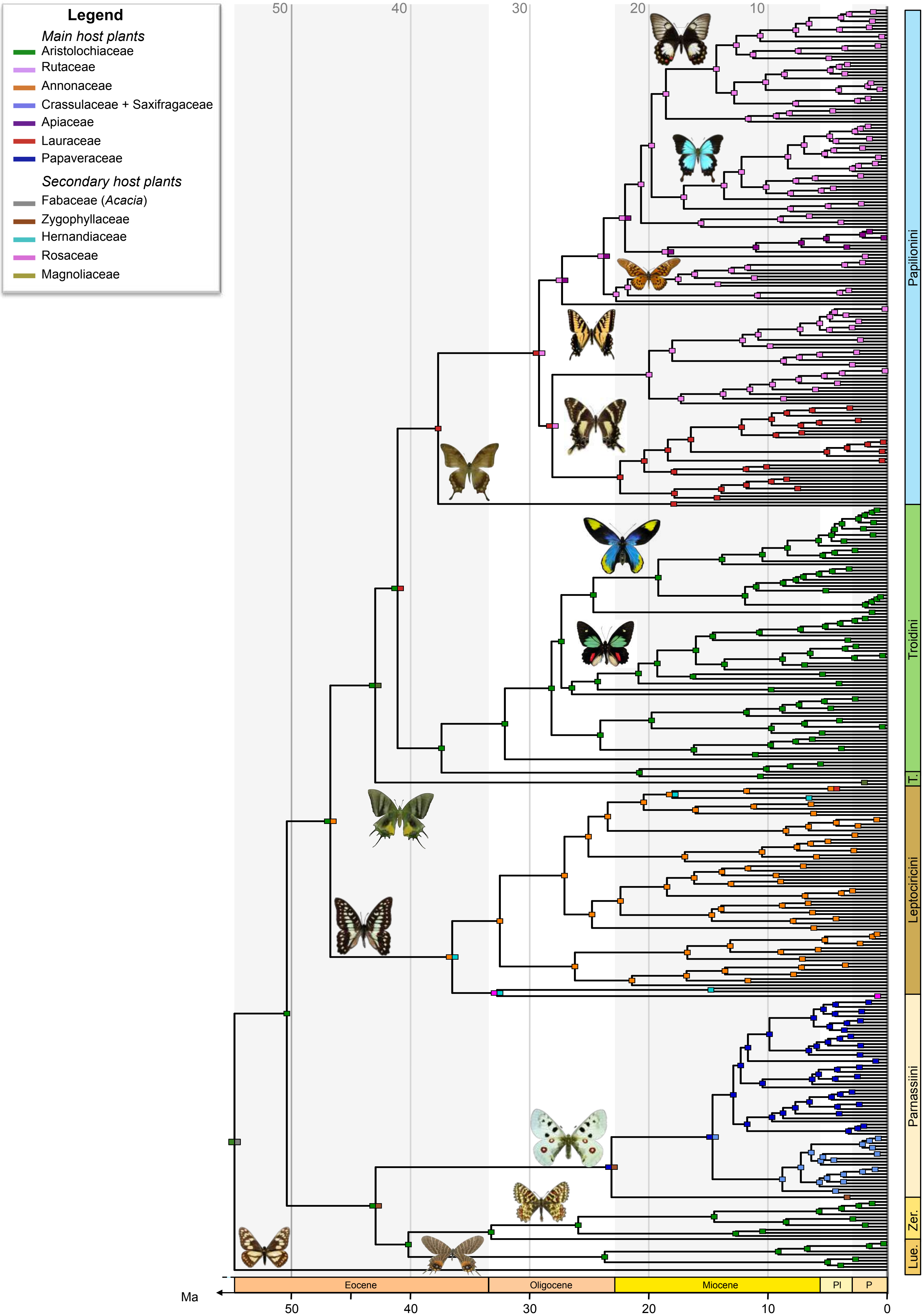

Legend
Main host plants
Aristolochiaceae
Rutaceae
Annonaceae
Crassulaceae + Saxifragaceae
Apiaceae
Lauraceae
Papaveraceae
Secondary host plants
Fabaceae (Acacia)
Zygophyllaceae
Hernandiaceae
Rosaceae
Magnoliaceae
50
40
30
20
10
Papilionini
Troidini
T.
Leptocircini
Parnassiini
Zer.
Lue.
Eocene
Oligocene
Miocene
Pl
P
Ma
50
40
30
20
10
0

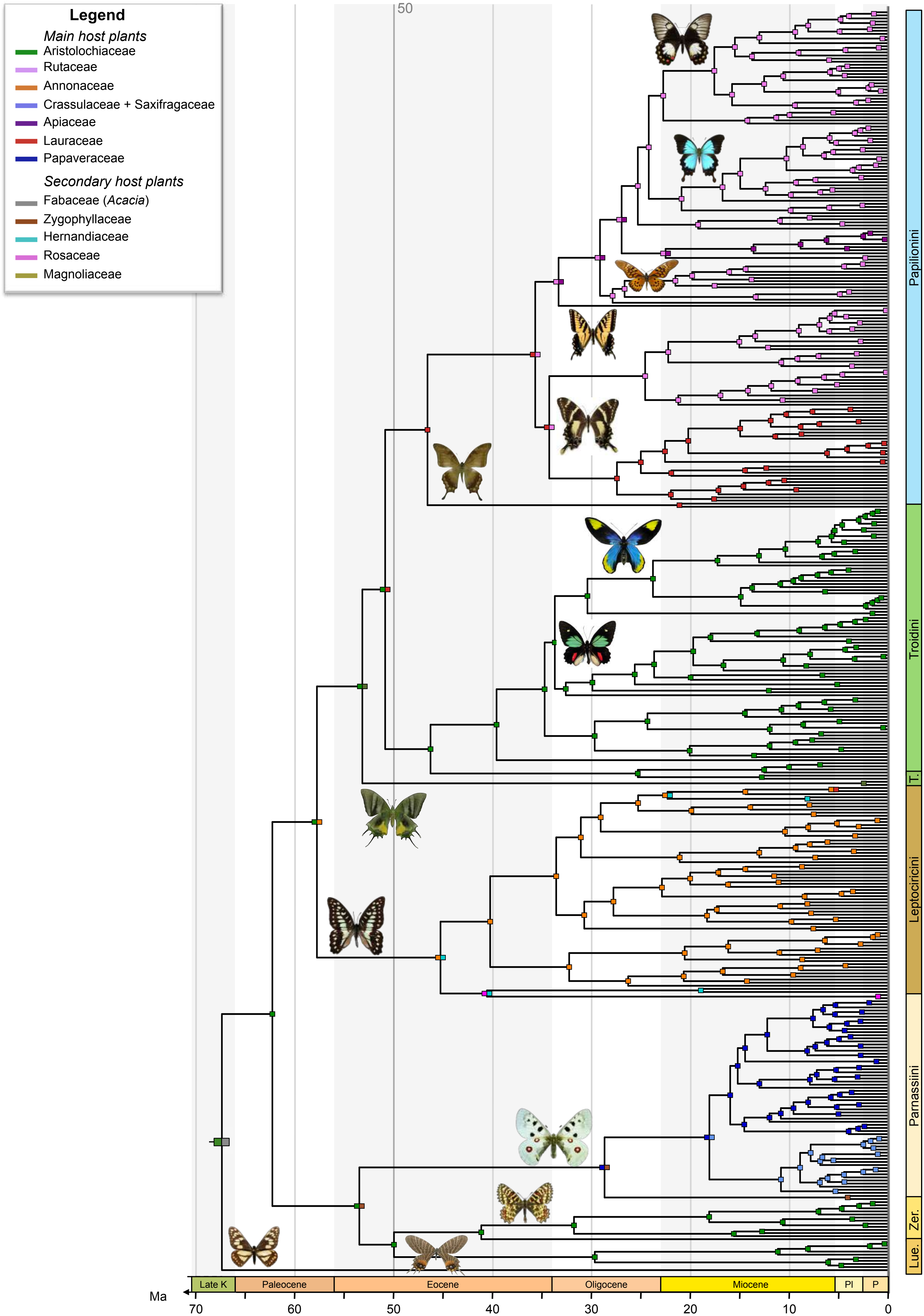
Legend
Main host plants
Aristolochiaceae
Rutaceae
Annonaceae
Crassulaceae + Saxifragaceae
Apiaceae
Lauraceae
Papaveraceae
Secondary host plants
Fabaceae (Acacia)
Zygophyllaceae
Hernandiaceae
Rosaceae
Magnoliaceae
50
Papilionini
Troidini
T.
Leptocircini
Parnassiini
Zer.
Lue.
Late K
Paleocene
Eocene
Oligocene
Miocene
Pl
P
Ma
70
60
50
40
30
20
10
0

Supplementary figure 5. Estimation of ancestral host-plant preferences with the maximum-likelihood model of Markov 1-parameter (Mk) and the Bayesian approach of BayesTraits. The results are represented by pie charts indicating the relative probability for each state inferred at a given node. The results consistently show that (1) the family Aristolochiaceae is recovered as the ancestral feeding habit of the Papilionidae, and (2) the host-plant shifts are recovered at the same nodes, except at the root of Papilionini and at the root of *Iphiclides* + *Lamproptera* (due to the fact the the Mk model can include only 10 states).

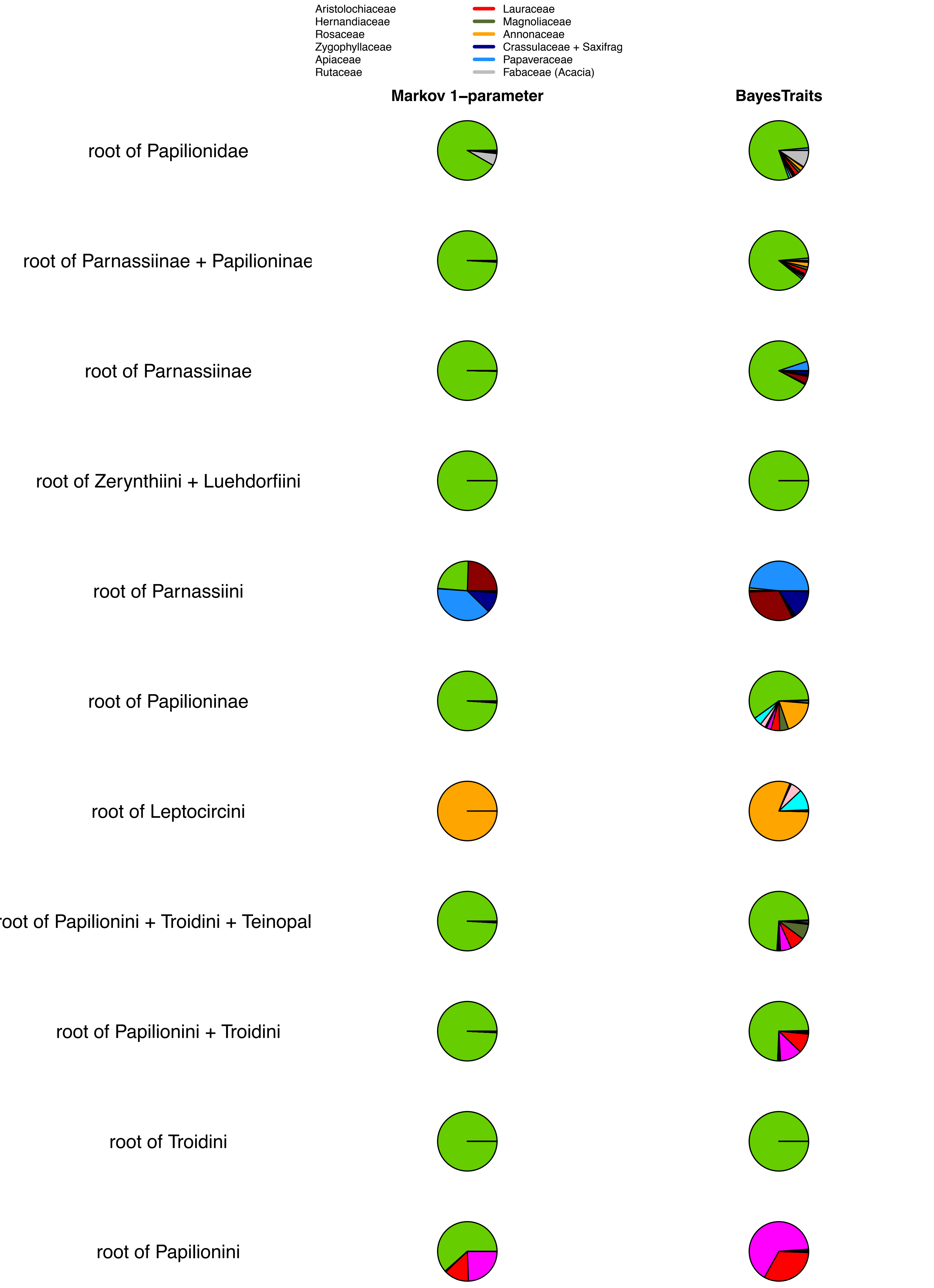
Aristolochiaceae
Hernandiaceae
Rosaceae
Zygophyllaceae
Apiaceae
Rutaceae
Lauraceae
Magnoliaceae
Annonaceae
Crassulaceae + Saxifrag
Papaveraceae
Fabaceae (Acacia)
Markov 1–parameter
BayesTraits
root of Papilionidae
root of Parnassiinae + Papilioninae
root of Parnassiinae
root of Zerynthiini + Luehdorfiini
root of Parnassiini
root of Papilioninae
root of Leptocircini
oot of Papilionini + Troidini + Teinopal
root of Papilionini + Troidini
root of Troidini
root of Papilionini

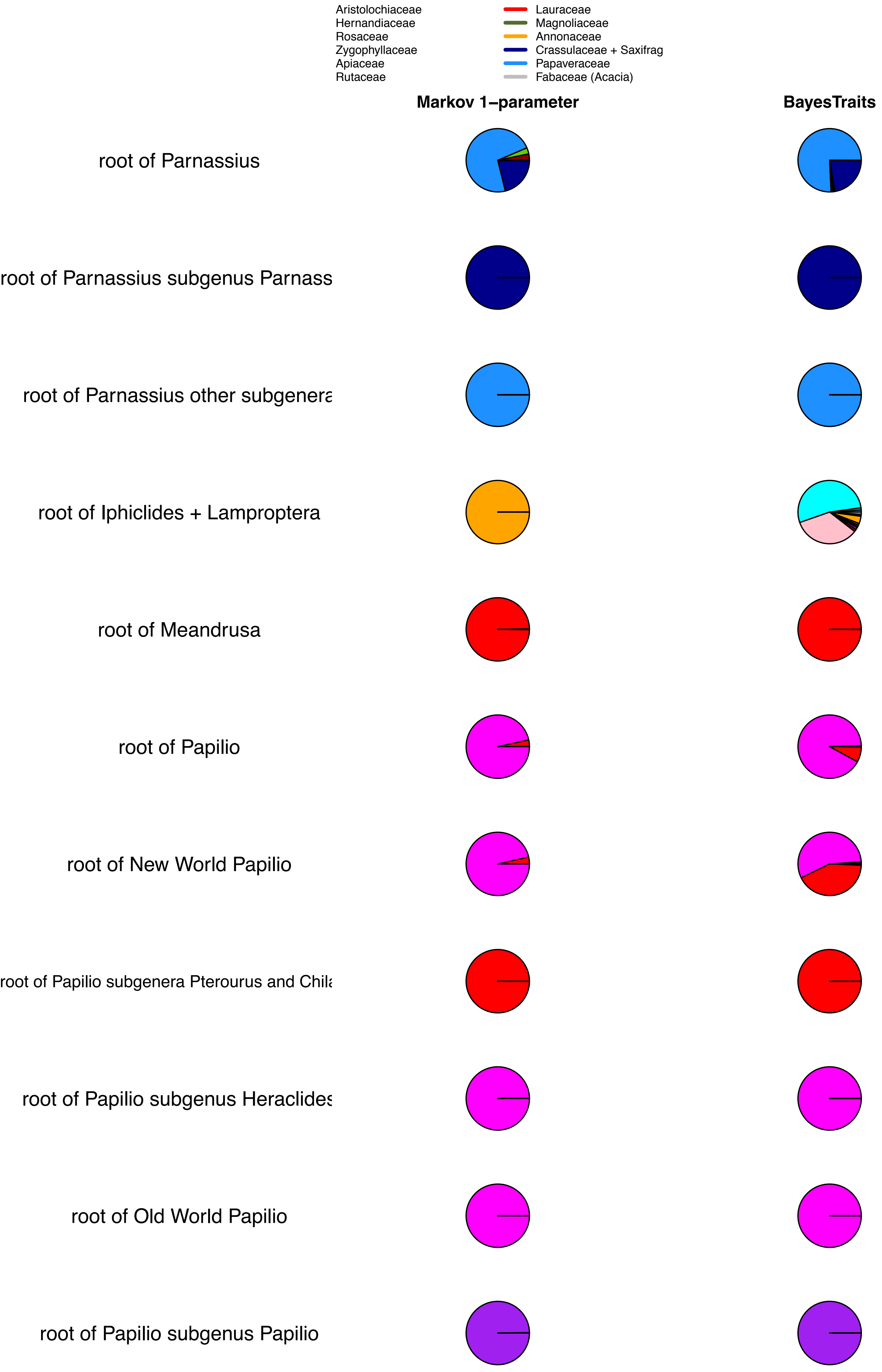

Aristolochiaceae
Hernandiaceae
Rosaceae
Zygophyllaceae
Apiaceae
Rutaceae
Lauraceae
Magnoliaceae
Annonaceae
Crassulaceae + Saxifrag
Papaveraceae
Fabaceae (Acacia)
Markov 1–parameter
BayesTraits
root of Parnassius
root of Parnassius subgenus Parnass
root of Parnassius other subgenera
root of Iphiclides + Lamproptera
root of Meandrusa
root of Papilio
root of New World Papilio
root of Papilio subgenera Pterourus and Chila
root of Papilio subgenus Heraclides
root of Old World Papilio
root of Papilio subgenus Papilio

Supplementary figure 6. Estimation of ancestral host-plant preferences for the Aristolochiaceae feeders with the Dispersal-Extinction-Cladogenesis (DEC) model. The results show that the genus *Aristolochia* is the primary Aristolochiaceae host plant while being also recovered as the ancestral feeding habit of the Papilionidae. Pictures of butterflies made by Fabien Condamine.

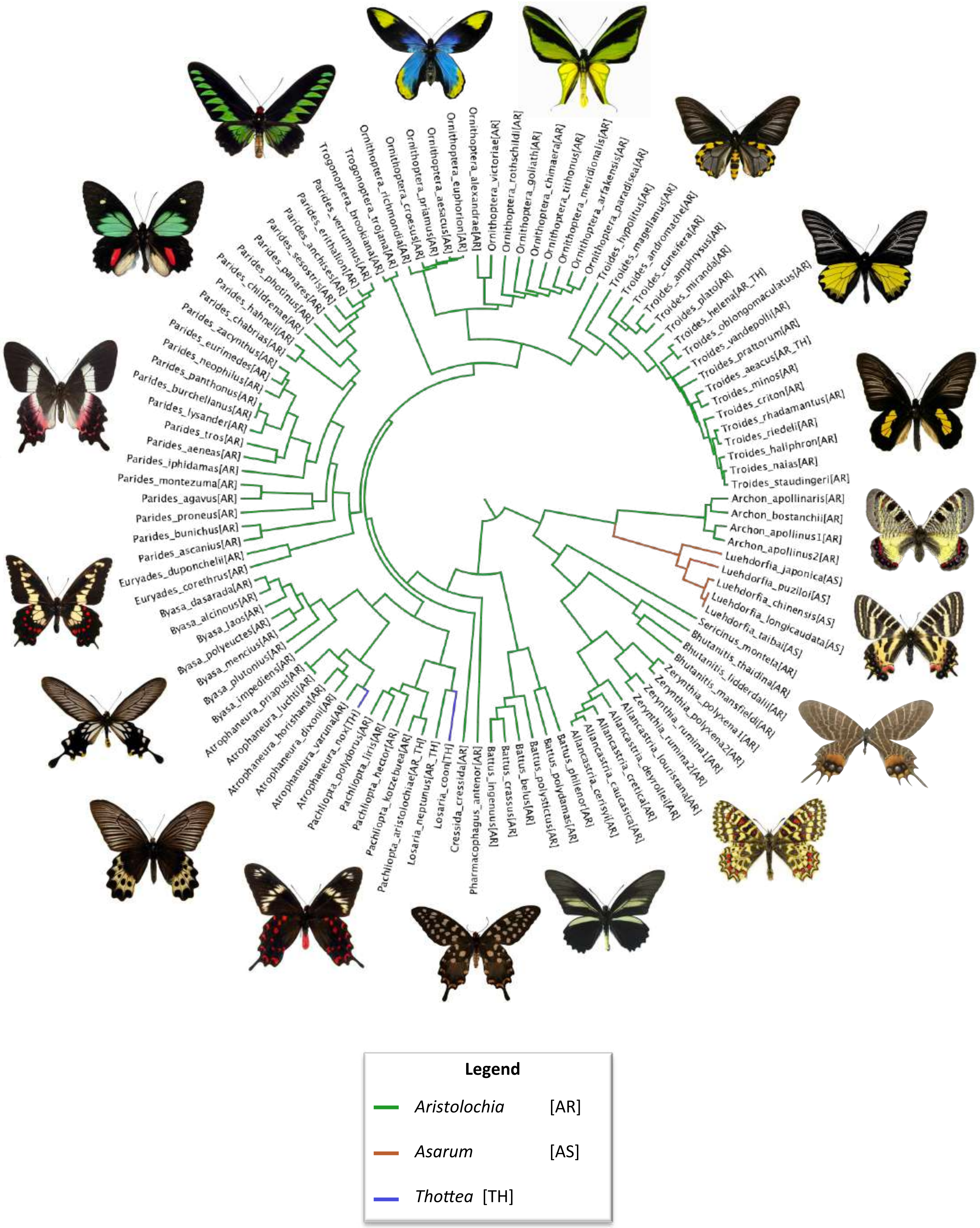
Ornithoptera_alexandrae[AR]
Ornithoptera_victoriae[AR]
Ornithoptera_rothschildi[AR]
Ornithoptera_goliath[AR]
Ornithoptera_chimaera[AR]
Ornithoptera_tithonus[AR]
Ornithoptera_meridionalis[AR]
Ornithoptera_arfakensis[AR]
Ornithoptera_paradisea[AR]
Troides_hypolitus[AR]
Troides_magellanus[AR]
Troides_andromache[AR]
Troides_cuneifera[AR]
Troides_amphrysus[AR]
Troides_miranda[AR]
Troides_plato[AR]
Troides_helena[AR_TH]
Troides_oblongomaculatus[AR]
Troides_vandepolli[AR]
Troides_prattorum[AR]
Troides_aeacus[AR_TH]
Troides_minos[AR]
Troides_criton[AR]
Troides_rhadamantus[AR]
Troides_riedeli[AR]
Troides_haliphron[AR]
Troides_naias[AR]
Troides_staudingeri[AR]
Archon_apollinaris[AR]
Archon_bostanchii[AR]
Archon_apollinus1[AR]
Archon_apollinus2[AR]
Luehdorfia_japonica[AS]
Luehdorfia_puziloi[AS]
Luehdorfia_chinensis[AS]
Luehdorfia_longicaudata[AS]
Luehdorfia_taibai[AS]
Sericinus_montela[AR]
Bhutanitis_thaidina[AR]
Bhutanitis_lidderdalii[AR]
Bhutanitis_mansfieldi[AR]
Zerynthia_polyxena1[AR]
Zerynthia_polyxena2[AR]
Zerynthia_rumina1[AR]
Zerynthia_rumina2[AR]
Allancastria_louristana[AR]
Allancastria_deyrollei[AR]
Allancastria_cretica[AR]
Allancastria_caucasica[AR]
Allancastria_cerisyi[AR]
Battus_philenor[AR]
Battus_polydamas[AR]
Battus_polystictus[AR]
Battus_belus[AR]
Battus_crassus[AR]
Battus_ingenuus[AR]
Pharmacophagus_antenor[AR]
Cressida_cressida[AR]
Losaria_coon[TH]
Losaria_neptunus[AR_TH]
Pachliopta_aristolochiae[AR_TH]
Pachliopta_kotzebuea[AR]
Pachliopta_hector[AR]
Pachliopta_liris[AR]
Pachliopta_polydorus[AR]
Atrophaneura_nox[TH]
Atrophaneura_varuna[AR]
Atrophaneura_dixoni[AR]
Atrophaneura_horishana[AR]
Atrophaneura_luchti[AR]
Atrophaneura_priapus[AR]
Byasa_impediens[AR]
Byasa_plutonius[AR]
Byasa_mencius[AR]
Byasa_polyeuctes[AR]
Byasa_laos[AR]
Byasa_alcinous[AR]
Byasa_dasarada[AR]
Euryades_corethrus[AR]
Euryades_duponchelii[AR]
Parides_ascanius[AR]
Parides_bunichus[AR]
Parides_proneus[AR]
Parides_agavus[AR]
Parides_montezuma[AR]
Parides_iphidamas[AR]
Parides_aeneas[AR]
Parides_tros[AR]
Parides_lysander[AR]
Parides_burchellanus[AR]
Parides_panthonus[AR]
Parides_neophilus[AR]
Parides_eurimedes[AR]
Parides_zacynthus[AR]
Parides_chabrias[AR]
Parides_hahneli[AR]
Parides_childrenae[AR]
Parides_photinus[AR]
Parides_panares[AR]
Parides_sesostris[AR]
Parides_anchises[AR]
Parides_erithalion[AR]
Parides_vertumnus[AR]
Trogonoptera_brookiana[AR]
Trogonoptera_trojana[AR]
Ornithoptera_richmondia[AR]
Ornithoptera_croesus[AR]
Ornithoptera_priamus[AR]
Ornithoptera_aesacus[AR]
Ornithoptera_euphorion[AR]
Legend
Aristolochia [AR]
Asarum [AS]
Thottea [TH]

Supplementary figure 7. Phylogenetic relationships within the Aristolochiaceae (perianth-bearing Piperales) for 247 species. The phylogeny is inferred with the Bayesian approach of MrBayes. Node support is indicated by posterior probabilities, with values ≥ 0.95 considered as strong node support. Pictures of plants made by Fabien Condamine.

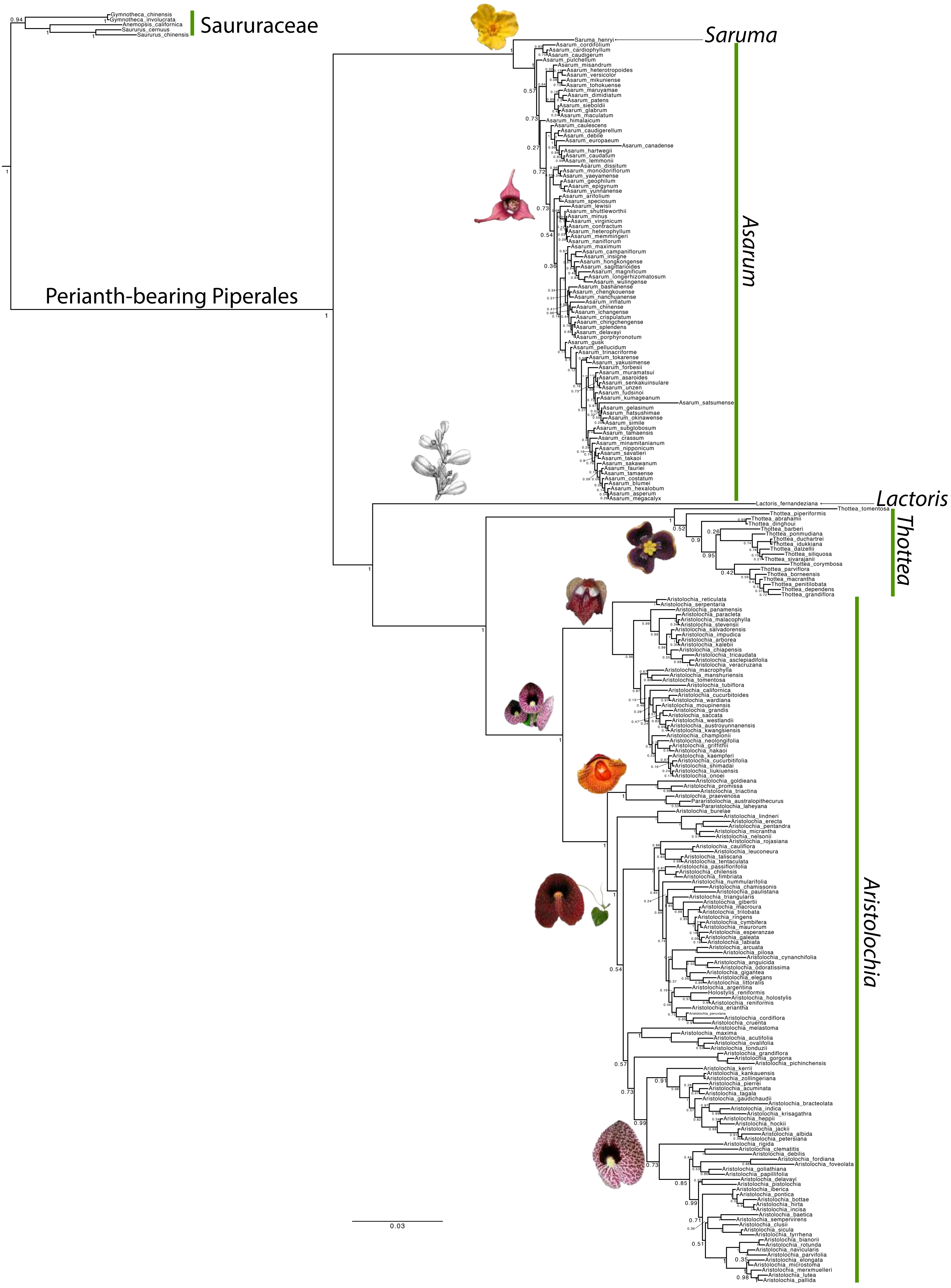

Sauruaceae
Gymnotheca_chinensis
Gymnotheca_involucrata
Anemopsis_californica
Saururus_cernuus
Saururus_chinensis
Perianth-bearing Piperales
Saruma
Saruma_henryi
Asarum
Asarum_cordifolium
Asarum_cardiophyllum
Asarum_caudigerum
Asarum_pulchellum
Asarum_misandrum
Asarum_heterotropoides
Asarum_versicolor
Asarum_mikuniense
Asarum_tohokuense
Asarum_maruyamae
Asarum_dimidiatum
Asarum_patens
Asarum_sieboldii
Asarum_glabrum
Asarum_maculatum
Asarum_himalaicum
Asarum_caulescens
Asarum_caudigerellum
Asarum_debile
Asarum_europaeum
Asarum_canadense
Asarum_hartwegii
Asarum_caudatum
Asarum_lemmonii
Asarum_dissitum
Asarum_monodoriflorum
Asarum_yaeyamense
Asarum_geophilum
Asarum_epigynum
Asarum_yunnanense
Asarum_arifolium
Asarum_speciosum
Asarum_lewisii
Asarum_shuttleworthii
Asarum_minus
Asarum_virginicum
Asarum_contractum
Asarum_heterophyllum
Asarum_memmingeri
Asarum_naniflorum
Asarum_maximum
Asarum_campaniflorum
Asarum_insigne
Asarum_hongkongense
Asarum_sagittarioides
Asarum_magnificum
Asarum_longerhizomatosum
Asarum_wulingense
Asarum_bashanense
Asarum_chengkouense
Asarum_nanchuanense
Asarum_inflatum
Asarum_chinense
Asarum_ichangense
Asarum_crispulatum
Asarum_chingchengense
Asarum_splendens
Asarum_delavayi
Asarum_porphyronotum
Asarum_gusk
Asarum_pellucidum
Asarum_trinacriforme
Asarum_tokarense
Asarum_yakusimense
Asarum_forbesii
Asarum_muramatsui
Asarum_asaroides
Asarum_senkakuinsulare
Asarum_unzen
Asarum_fudsinoi
Asarum_kumageanum
Asarum_satsumense
Asarum_gelasinum
Asarum_hatsushimae
Asarum_okinawense
Asarum_simile
Asarum_subglobosum
Asarum_tamaensis
Asarum_crassum
Asarum_minamitanianum
Asarum_nipponicum
Asarum_savatieri
Asarum_takaoi
Asarum_sakawanum
Asarum_fauriei
Asarum_tamaense
Asarum_costatum
Asarum_blumei
Asarum_hexalobum
Asarum_asperum
Asarum_megacalyx
Lactoris
Lactoris_fernandeziana
Thottea
Thottea_tomentosa
Thottea_piperiformis
Thottea_abrahamii
Thottea_dinghoui
Thottea_barberi
Thottea_ponmudiana
Thottea_duchartrei
Thottea_idukkiana
Thottea_dalzellii
Thottea_siliquosa
Thottea_sivarajanii
Thottea_corymbosa
Thottea_parviflora
Thottea_borneensis
Thottea_macrantha
Thottea_penitilobata
Thottea_dependens
Thottea_grandiflora
Aristolochia
Aristolochia_reticulata
Aristolochia_serpentaria
Aristolochia_panamensis
Aristolochia_paracleta
Aristolochia_malacophylla
Aristolochia_stevensii
Aristolochia_salvadorensis
Aristolochia_impudica
Aristolochia_arborea
Aristolochia_kalebii
Aristolochia_chiapensis
Aristolochia_tricaudata
Aristolochia_asclepiadifolia
Aristolochia_veracruzana
Aristolochia_macrophylla
Aristolochia_manshuriensis
Aristolochia_tomentosa
Aristolochia_tubiflora
Aristolochia_californica
Aristolochia_cucurbitoides
Aristolochia_wardiana
Aristolochia_moupinensis
Aristolochia_grandis
Aristolochia_saccata
Aristolochia_westlandii
Aristolochia_austroyunnanensis
Aristolochia_kwangsiensis
Aristolochia_championii
Aristolochia_neolongifolia
Aristolochia_griffithii
Aristolochia_nakaoi
Aristolochia_kaempferi
Aristolochia_cucurbitifolia
Aristolochia_shimadai
Aristolochia_liukiuensis
Aristolochia_onoei
Aristolochia_goldieana
Aristolochia_promissa
Aristolochia_triactina
Aristolochia_praevenosa
Pararistolochia_australopithecurus
Pararistolochia_laheyana
Aristolochia_burelae
Aristolochia_lindneri
Aristolochia_erecta
Aristolochia_pentandra
Aristolochia_micrantha
Aristolochia_nelsonii
Aristolochia_rojasiana
Aristolochia_cauliflora
Aristolochia_leuconeura
Aristolochia_taliscana
Aristolochia_tentaculata
Aristolochia_passiflorifolia
Aristolochia_chilensis
Aristolochia_fimbriata
Aristolochia_nummularifolia
Aristolochia_chamissonis
Aristolochia_paulistana
Aristolochia_triangularis
Aristolochia_gibertii
Aristolochia_macroura
Aristolochia_trilobata
Aristolochia_ringens
Aristolochia_cymbifera
Aristolochia_maurorum
Aristolochia_esperanzae
Aristolochia_galeata
Aristolochia_labiata
Aristolochia_arcuata
Aristolochia_pilosa
Aristolochia_cynanchifolia
Aristolochia_anguicida
Aristolochia_odoratissima
Aristolochia_gigantea
Aristolochia_elegans
Aristolochia_littoralis
Aristolochia_argentina
Holostylis_reniformis
Aristolochia_holostylis
Aristolochia_reniformis
Aristolochia_eriantha
Aristolochia_peruviana
Aristolochia_cordiflora
Aristolochia_cruenta
Aristolochia_melastoma
Aristolochia_maxima
Aristolochia_acutifolia
Aristolochia_ovalifolia
Aristolochia_tonduzii
Aristolochia_grandiflora
Aristolochia_gorgona
Aristolochia_pichinchensis
Aristolochia_kerrii
Aristolochia_kankauensis
Aristolochia_zollingeriana
Aristolochia_pierrei
Aristolochia_acuminata
Aristolochia_tagala
Aristolochia_gaudichaudii
Aristolochia_bracteolata
Aristolochia_indica
Aristolochia_krisagathra
Aristolochia_heppii
Aristolochia_hockii
Aristolochia_jackii
Aristolochia_albida
Aristolochia_petersiana
Aristolochia_rigida
Aristolochia_clematitis
Aristolochia_debilis
Aristolochia_fordiana
Aristolochia_foveolata
Aristolochia_goliathiana
Aristolochia_papillifolia
Aristolochia_delavayi
Aristolochia_pistolochia
Aristolochia_iberica
Aristolochia_pontica
Aristolochia_bottae
Aristolochia_hirta
Aristolochia_incisa
Aristolochia_baetica
Aristolochia_sempervirens
Aristolochia_clusii
Aristolochia_sicula
Aristolochia_tyrrhena
Aristolochia_bianorii
Aristolochia_rotunda
Aristolochia_navicularis
Aristolochia_parvifolia
Aristolochia_elongata
Aristolochia_microstoma
Aristolochia_merxmuelleri
Aristolochia_lutea
Aristolochia_pallida
0.03

Supplementary figure 8. Bayesian estimates of divergence times for Aristolochiaceae. The first inference was performed with exponential priors on fossil calibrations and 150 Ma as maximum age. The second inference was performed with exponential priors on fossil calibrations and 221 Ma as maximum age. The third inference was performed with uniform priors on fossil calibrations and 150 Ma as maximum age. The fourth inference was performed with uniform priors on fossil calibrations and 221 Ma as maximum age. The origin of the genus *Aristolochia* is estimated at 55.5 Ma (95% CI: 39.2-72.8 Ma) in the first analysis, at 58.8 Ma (95% CI: 42.5-76.2 Ma) in the second analysis, at 60.7 Ma (95% CI: 43.9-80.5 Ma) in the third analysis, and at 64.8 Ma (95% CI: 47.3-83.1 Ma) in the fourth analysis.

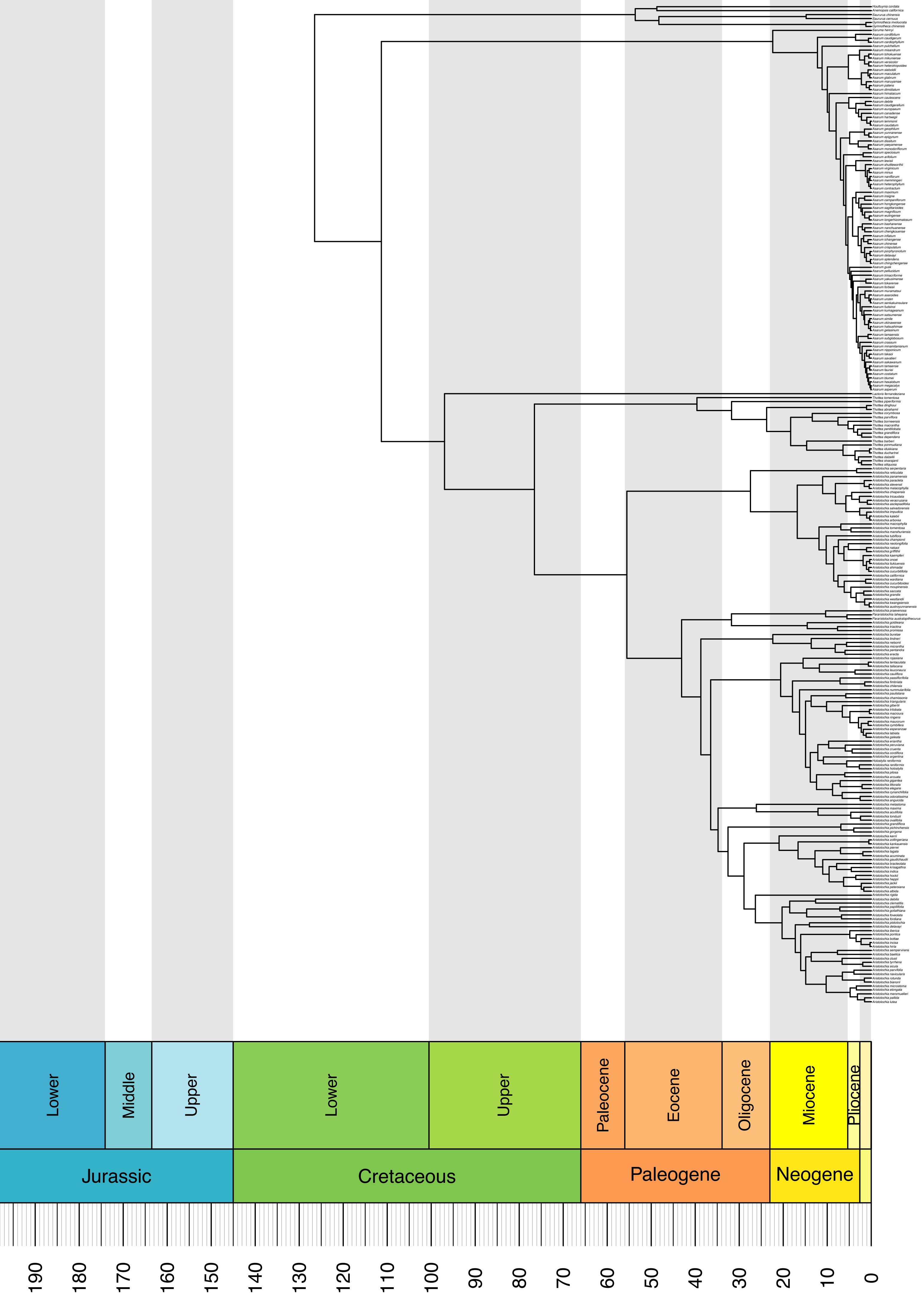

Lower
Middle
Upper
Lower
Upper
Paleocene
Eocene
Oligocene
Miocene
Pliocene
Jurassic
Cretaceous
Paleogene
Neogene
190
180
170
160
150
140
130
120
110
100
90
80
70
60
50
40
30
20
10
0

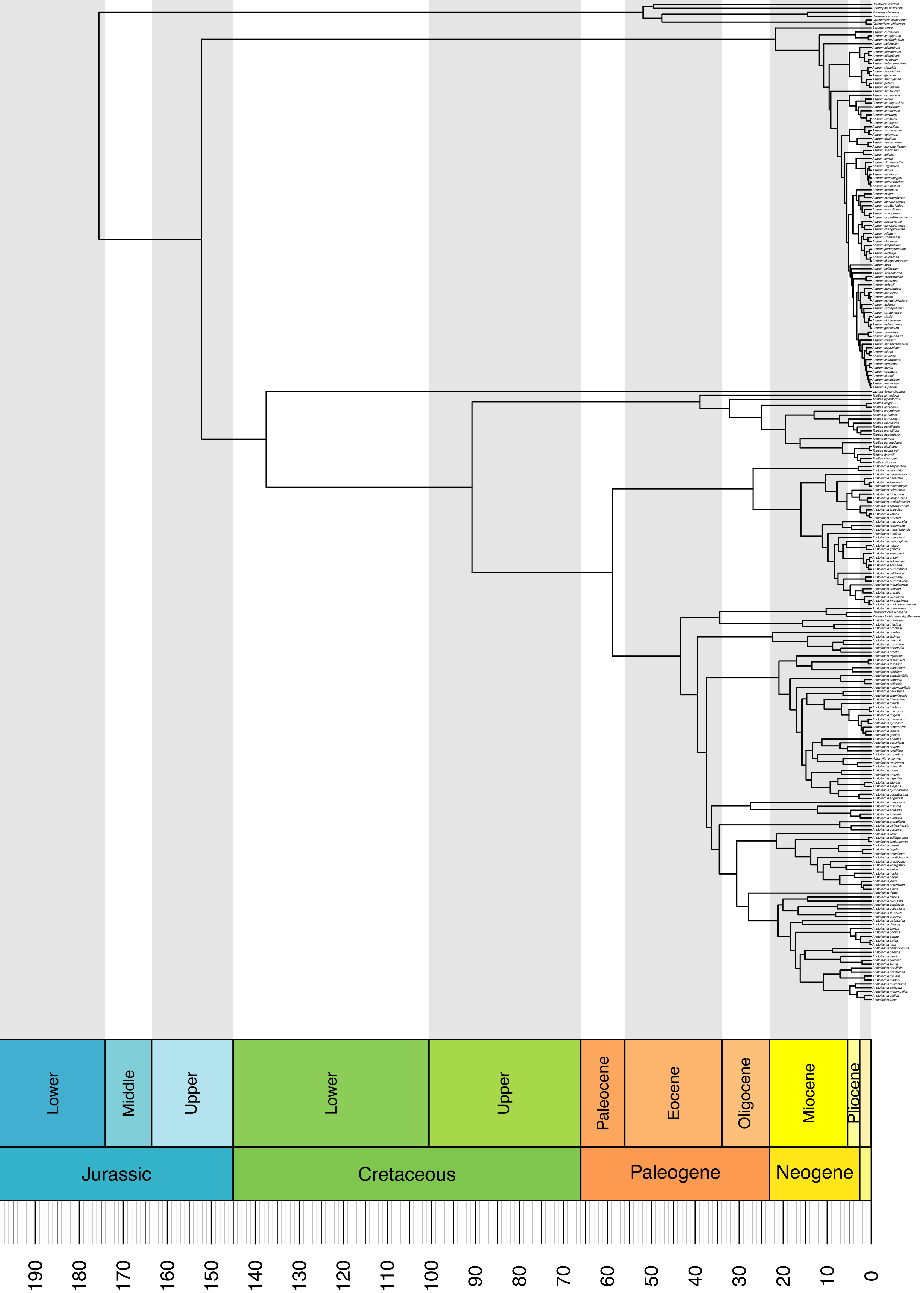

Lower
Middle
Upper
Lower
Upper
Paleocene
Eocene
Oligocene
Miocene
Pliocene
Jurassic
Cretaceous
Paleogene
Neogene
190
180
170
160
150
140
130
120
110
100
90
80
70
60
50
40
30
20
10
0

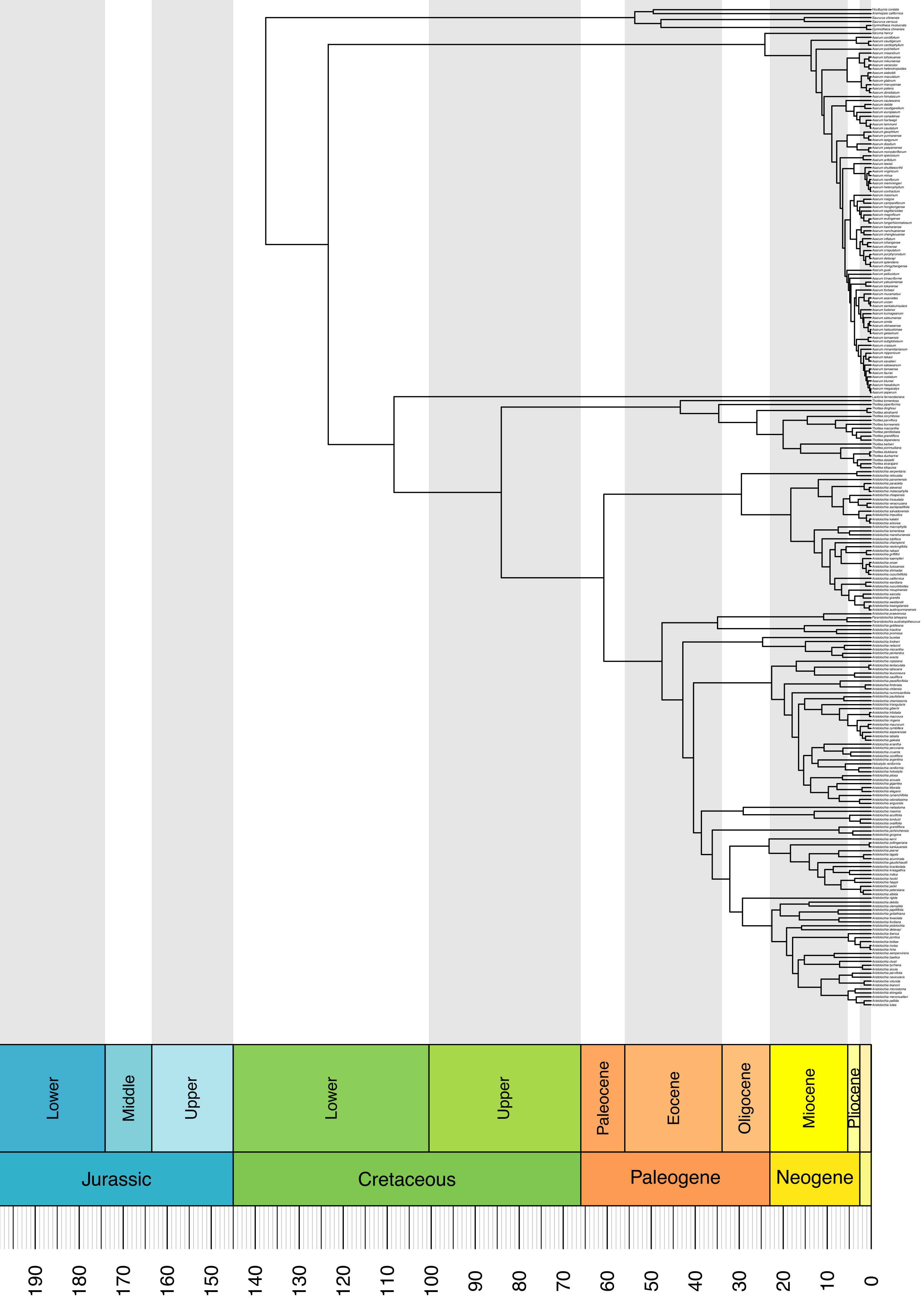

Lower
Middle
Upper
Lower
Upper
Paleocene
Eocene
Oligocene
Miocene
Pliocene
Jurassic
Cretaceous
Paleogene
Neogene
190
180
170
160
150
140
130
120
110
100
90
80
70
60
50
40
30
20
10
0

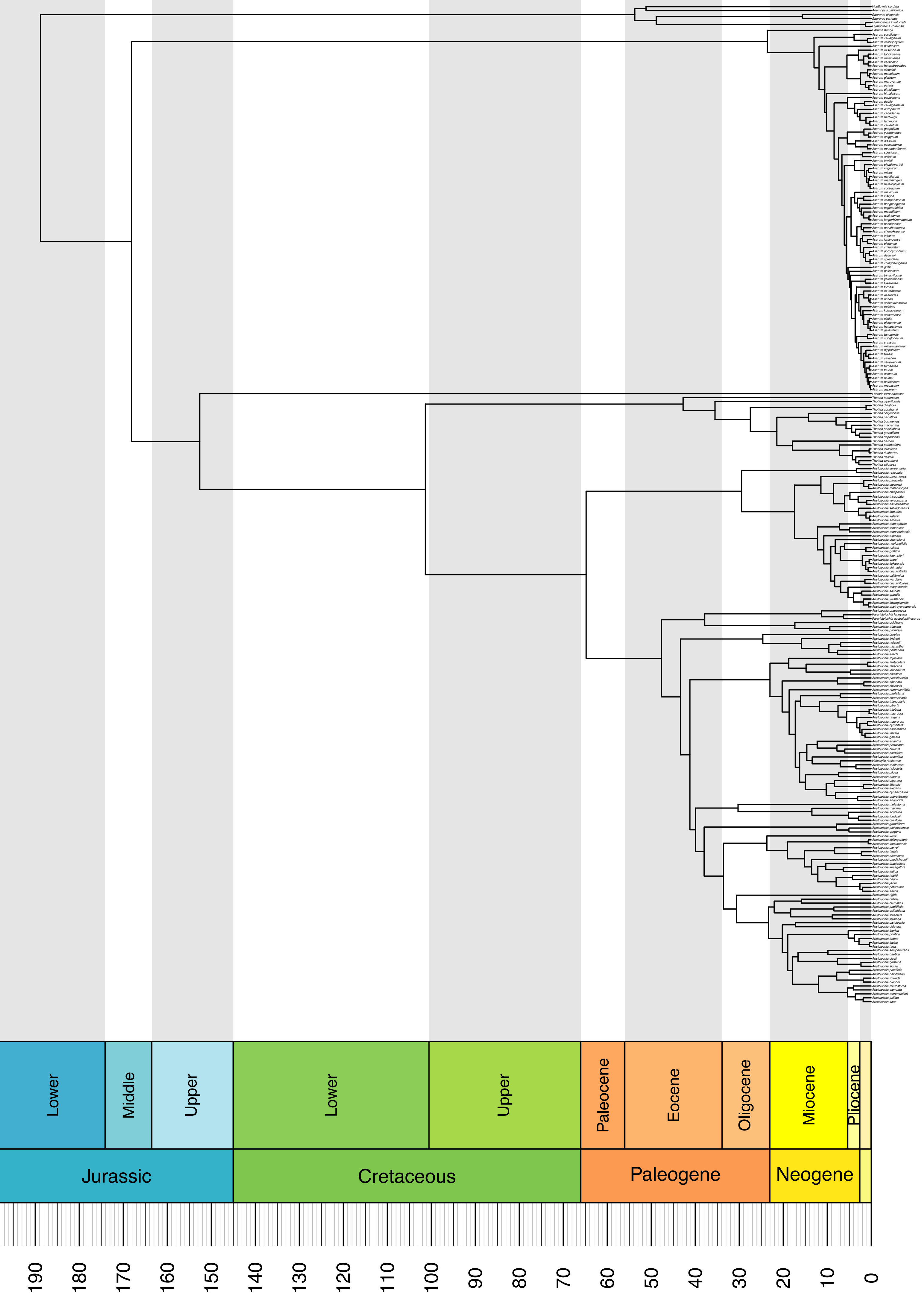

Lower
Middle
Upper
Lower
Upper
Paleocene
Eocene
Oligocene
Miocene
Pliocene
Jurassic
Cretaceous
Paleogene
Neogene
190
180
170
160
150
140
130
120
110
100
90
80
70
60
50
40
30
20
10
0

Supplementary figure 9. Median node ages and 95% credibility intervals (CI) for the two dating analyses of Papilionidae and the four dating analyses of Aristolochiaceae. The 95% CI overlap substantially between the two groups regardless of the dating analysis. J = Jurassic, Pl = Pliocene, P = Pleistocene. Pictures of the plant and butterfly made by Fabien Condamine.

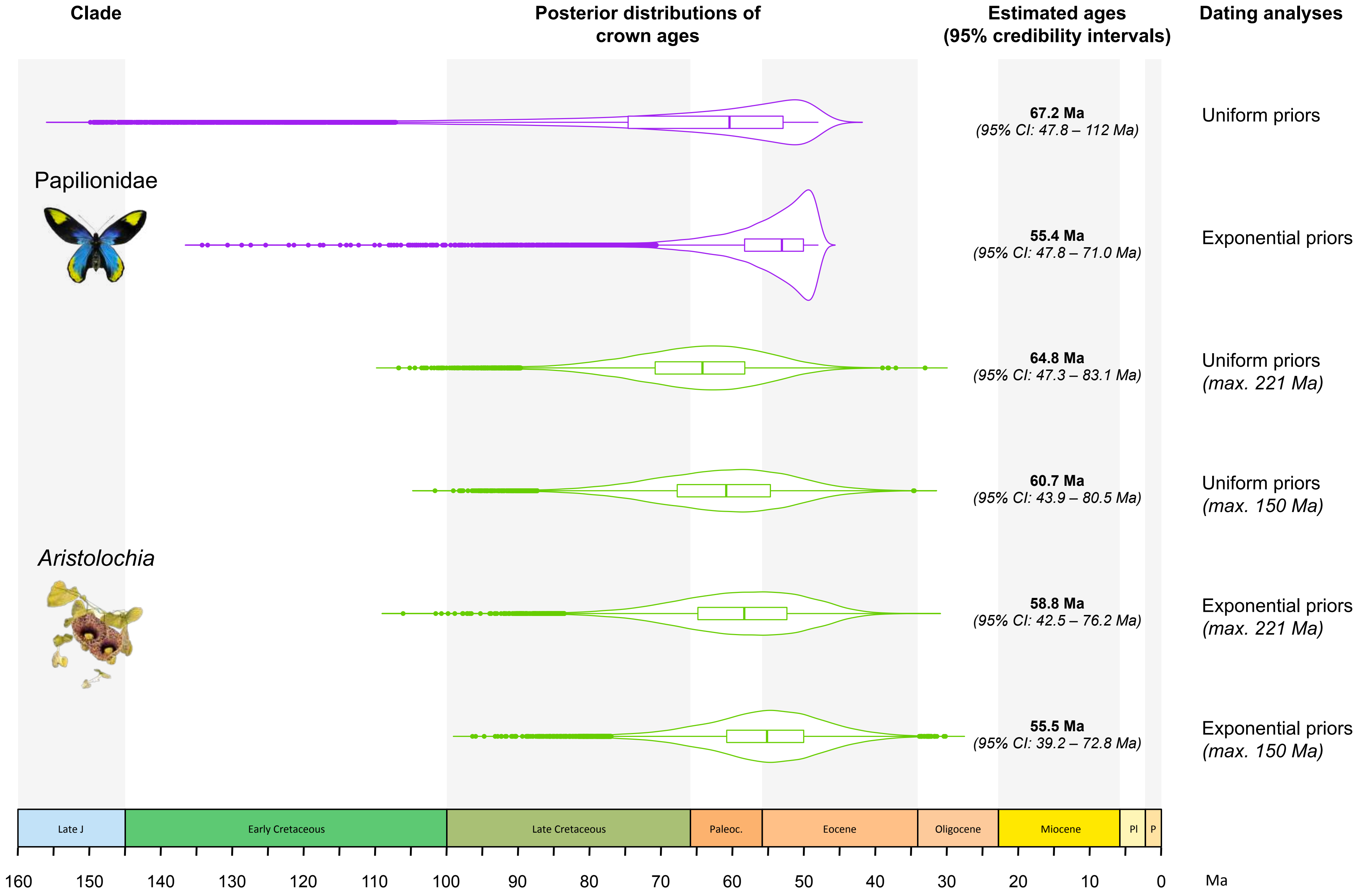

Clade
Posterior distributions of crown ages
Estimated ages (95% credibility intervals)
Dating analyses
Papilionidae
Aristolochia
67.2 Ma
(95% CI: 47.8 – 112 Ma)
Uniform priors
55.4 Ma
(95% CI: 47.8 – 71.0 Ma)
Exponential priors
64.8 Ma
(95% CI: 47.3 – 83.1 Ma)
Uniform priors
(max. 221 Ma)
60.7 Ma
(95% CI: 43.9 – 80.5 Ma)
Uniform priors
(max. 150 Ma)
58.8 Ma
(95% CI: 42.5 – 76.2 Ma)
Exponential priors
(max. 221 Ma)
55.5 Ma
(95% CI: 39.2 – 72.8 Ma)
Exponential priors
(max. 150 Ma)
Late J
Early Cretaceous
Late Cretaceous
Paleoc.
Eocene
Oligocene
Miocene
Pl
P
160
150
140
130
120
110
100
90
80
70
60
50
40
30
20
10
0
Ma

Supplementary figure 10. Estimation of the historical biogeography for the two molecular dated trees of Papilionidae with the Dispersal-Extinction-Cladogenesis (DEC) model. For each tree, two DEC analyses were performed: one with time-stratified palaeogeographic constraints, and one without such constraints. The swallowtail butterflies originated in a northern region centred around the Bering land bridge. K = Cretaceous, Pl = Pliocene, P = Pleistocene. Pictures of the butterflies and the world maps made by Fabien Condamine.

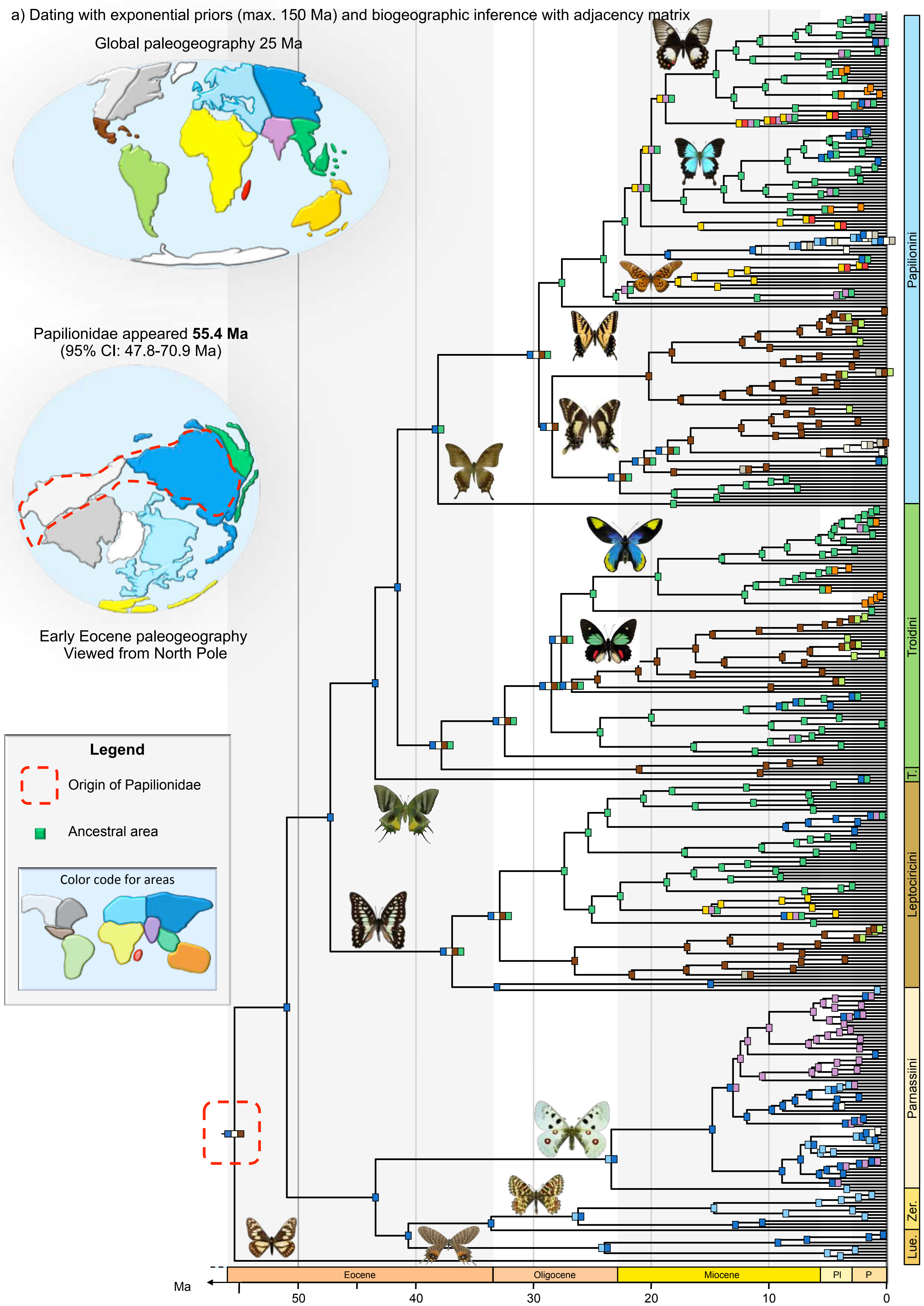
a) Dating with exponential priors (max. 150 Ma) and biogeographic inference with adjacency matrix
Global paleogeography 25 Ma
Papilionidae appeared **55.4 Ma**
(95% CI: 47.8-70.9 Ma)
Early Eocene paleogeography
Viewed from North Pole
Legend
Origin of Papilionidae
Ancestral area
Color code for areas
Papilionini
Troidini
T.
Leptocircini
Parnassiini
Zer.
Lue.
Eocene
Oligocene
Miocene
Pl
P
Ma
50
40
30
20
10
0

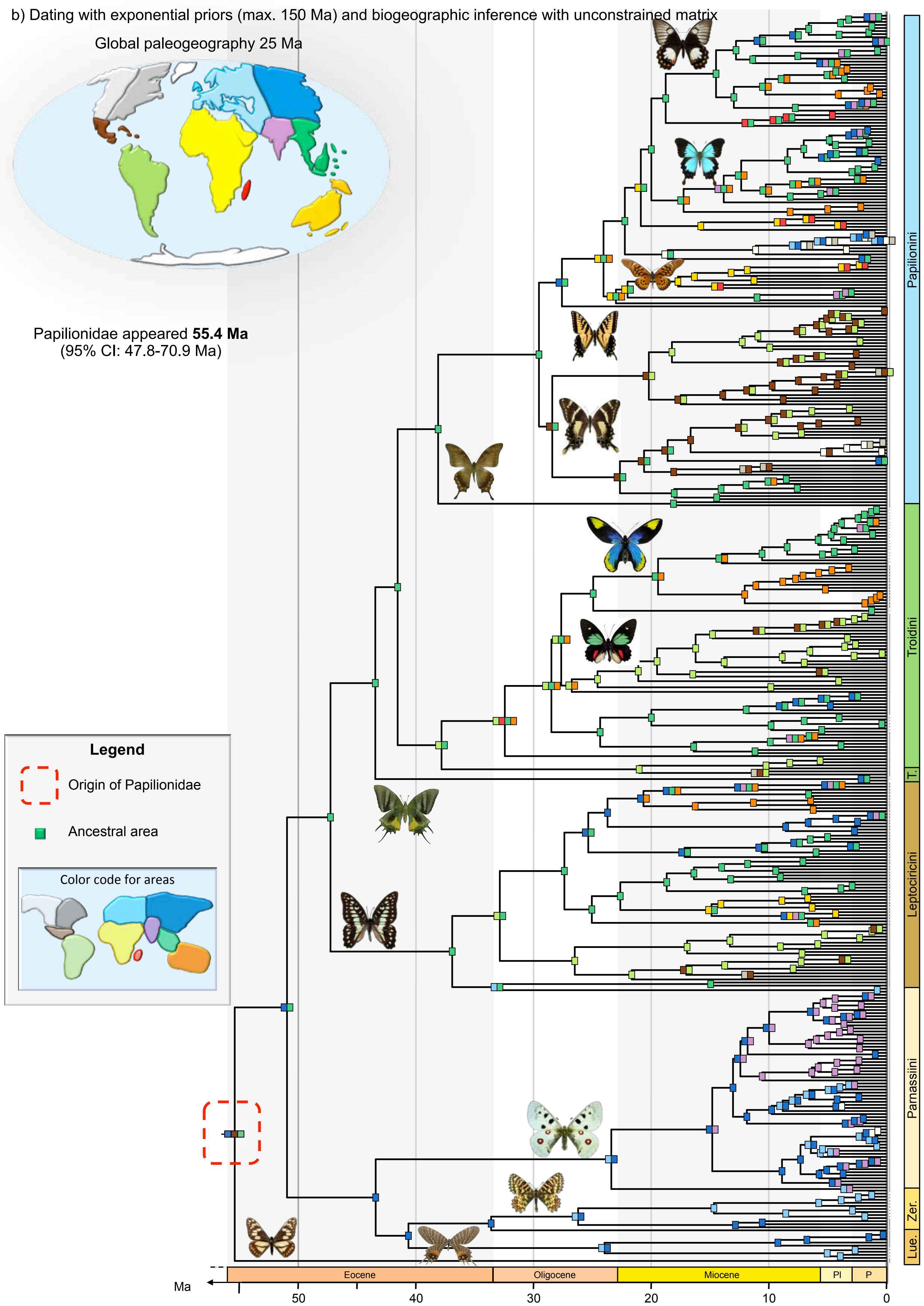
b) Dating with exponential priors (max. 150 Ma) and biogeographic inference with unconstrained matrix
Global paleogeography 25 Ma
Papilionidae appeared 55.4 Ma
(95% CI: 47.8-70.9 Ma)
Legend
Origin of Papilionidae
Ancestral area
Color code for areas
Papilionini
Troidini
T.
Leptocircini
Parnassiini
Zer.
Lue.
Eocene
Oligocene
Miocene
Pl
P
Ma
50
40
30
20
10
0

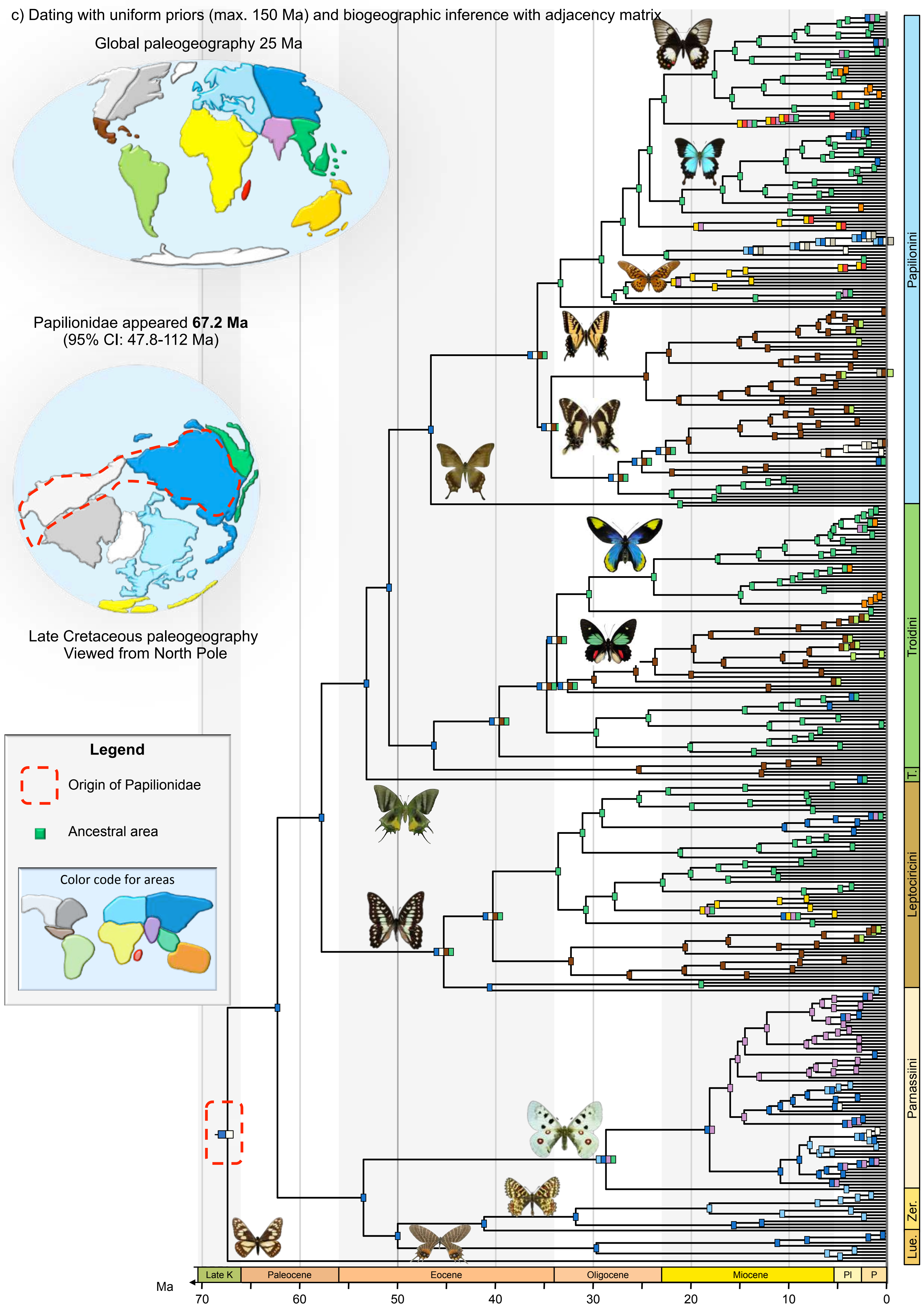

c) Dating with uniform priors (max. 150 Ma) and biogeographic inference with adjacency matrix
Global paleogeography 25 Ma
Papilionidae appeared 67.2 Ma
(95% CI: 47.8-112 Ma)
Late Cretaceous paleogeography
Viewed from North Pole
Legend
Origin of Papilionidae
Ancestral area
Color code for areas
Papilionini
Troidini
T.
Leptocircini
Parnassiini
Zer.
Lue.
Late K
Paleocene
Eocene
Oligocene
Miocene
Pl
P
Ma
70
60
50
40
30
20
10
0

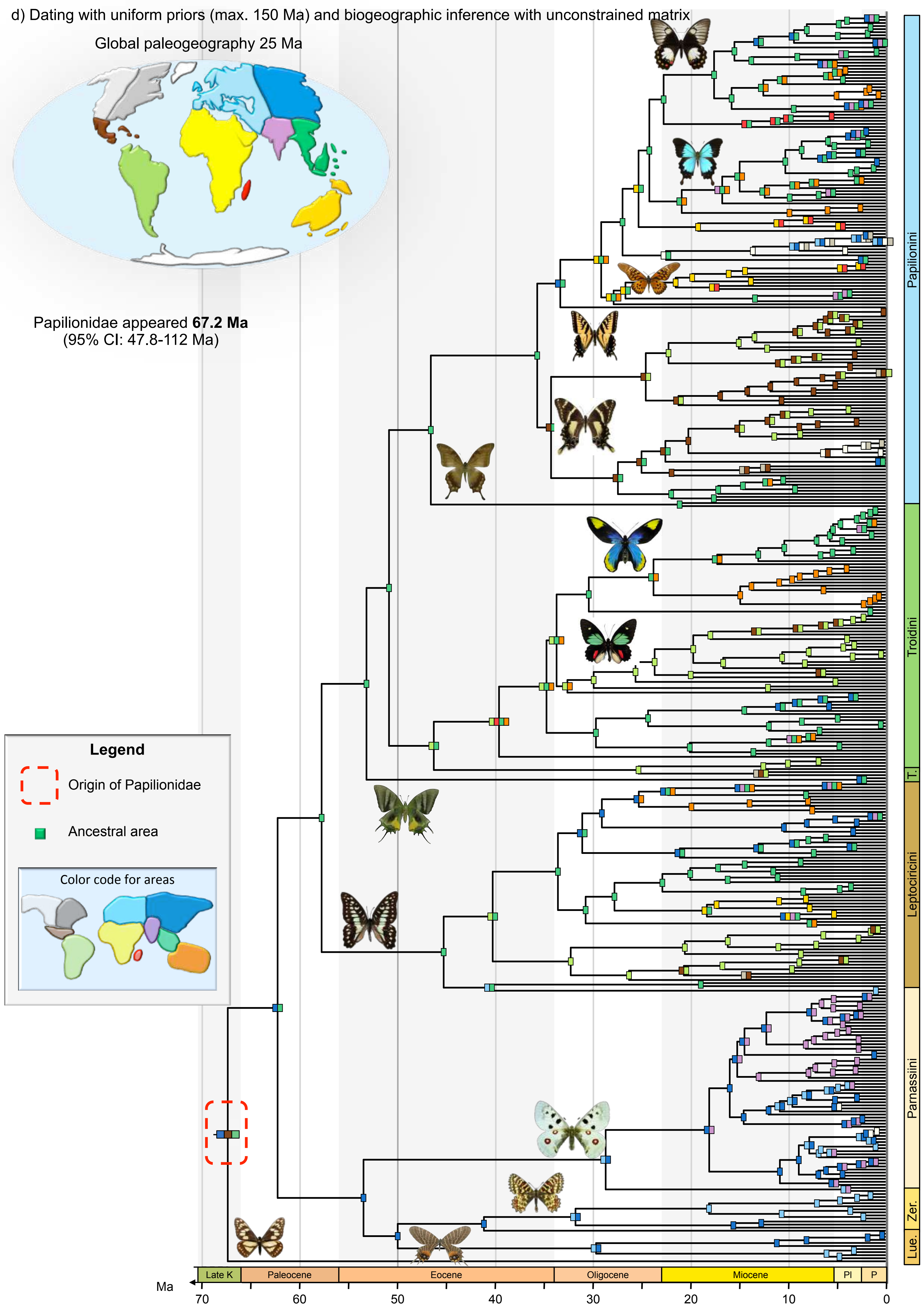

d) Dating with uniform priors (max. 150 Ma) and biogeographic inference with unconstrained matrix
Global paleogeography 25 Ma
Papilionidae appeared **67.2 Ma**
(95% CI: 47.8-112 Ma)
Legend
Origin of Papilionidae
Ancestral area
Color code for areas
Papilionini
Troidini
T.
Leptocircini
Parnassiini
Zer.
Lue.
Late K
Paleocene
Eocene
Oligocene
Miocene
Pl
P
Ma
70
60
50
40
30
20
10
0

Supplementary figure 11. Estimation of the historical biogeography for the four molecular dated trees of Aristolochiaceae with the Dispersal-Extinction-Cladogenesis (DEC) model. For each tree, two DEC analyses were performed: one with time-stratified palaeogeographic constraints, and one without such constraints. The genus *Aristolochia* originated in a northern region centred around the Bering land bridge. J = Jurassic, K = Cretaceous, Pl = Pliocene, P = Pleistocene. Pictures of the plants and the world maps made by Fabien Condamine.

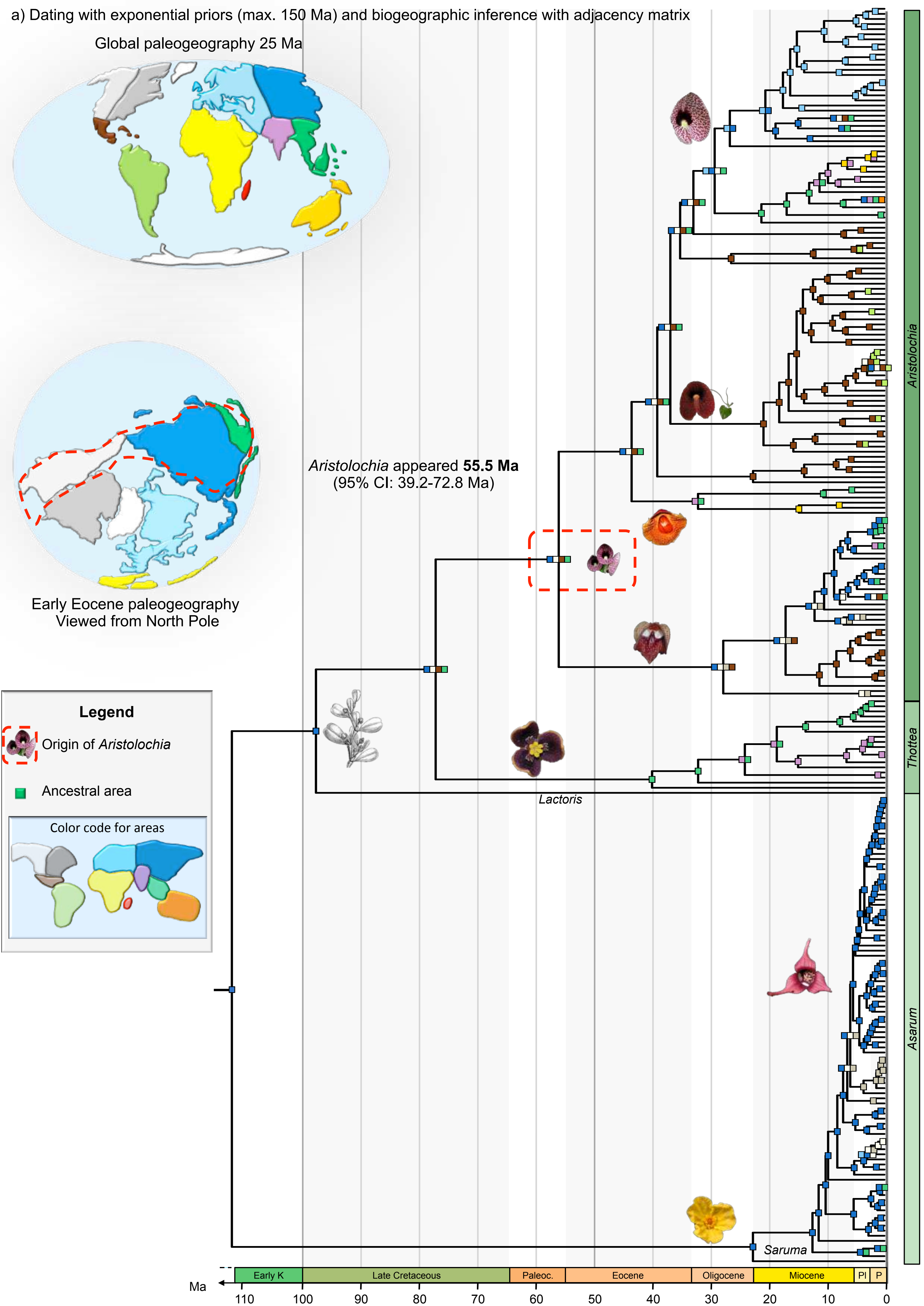

a) Dating with exponential priors (max. 150 Ma) and biogeographic inference with adjacency matrix
Global paleogeography 25 Ma
Early Eocene paleogeography
Viewed from North Pole
Aristolochia appeared 55.5 Ma
(95% CI: 39.2-72.8 Ma)
Legend
Origin of Aristolochia
Ancestral area
Color code for areas
Lactoris
Saruma
Aristolochia
Thottea
Asarum
Early K
Late Cretaceous
Paleoc.
Eocene
Oligocene
Miocene
Pl
P
Ma
110
100
90
80
70
60
50
40
30
20
10
0

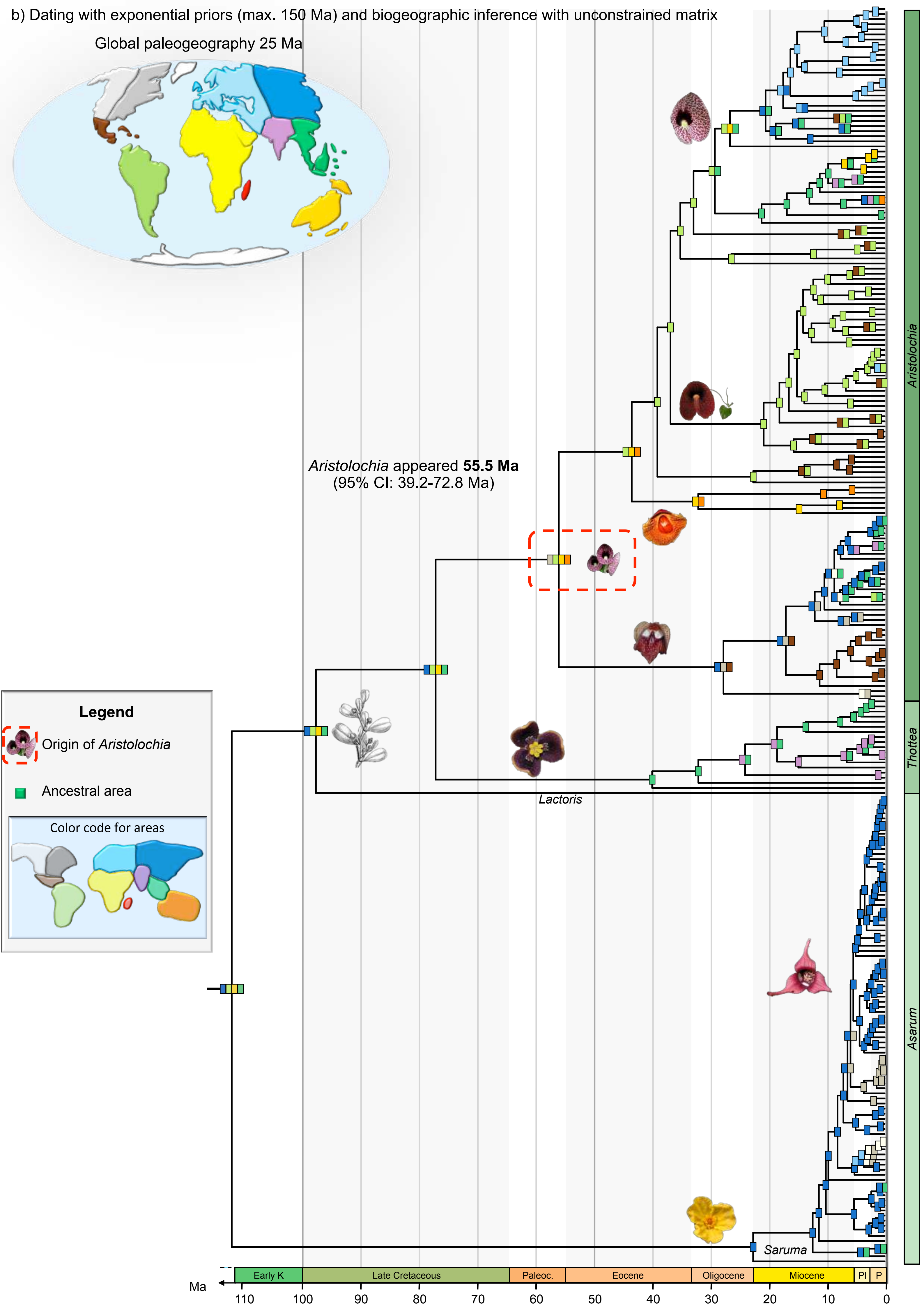

b) Dating with exponential priors (max. 150 Ma) and biogeographic inference with unconstrained matrix
Global paleogeography 25 Ma
Aristolochia appeared 55.5 Ma
(95% CI: 39.2-72.8 Ma)
Legend
Origin of Aristolochia
Ancestral area
Color code for areas
Lactoris
Saruma
Aristolochia
Thottea
Asarum
Early K
Late Cretaceous
Paleoc.
Eocene
Oligocene
Miocene
Pl
P
Ma
110
100
90
80
70
60
50
40
30
20
10
0

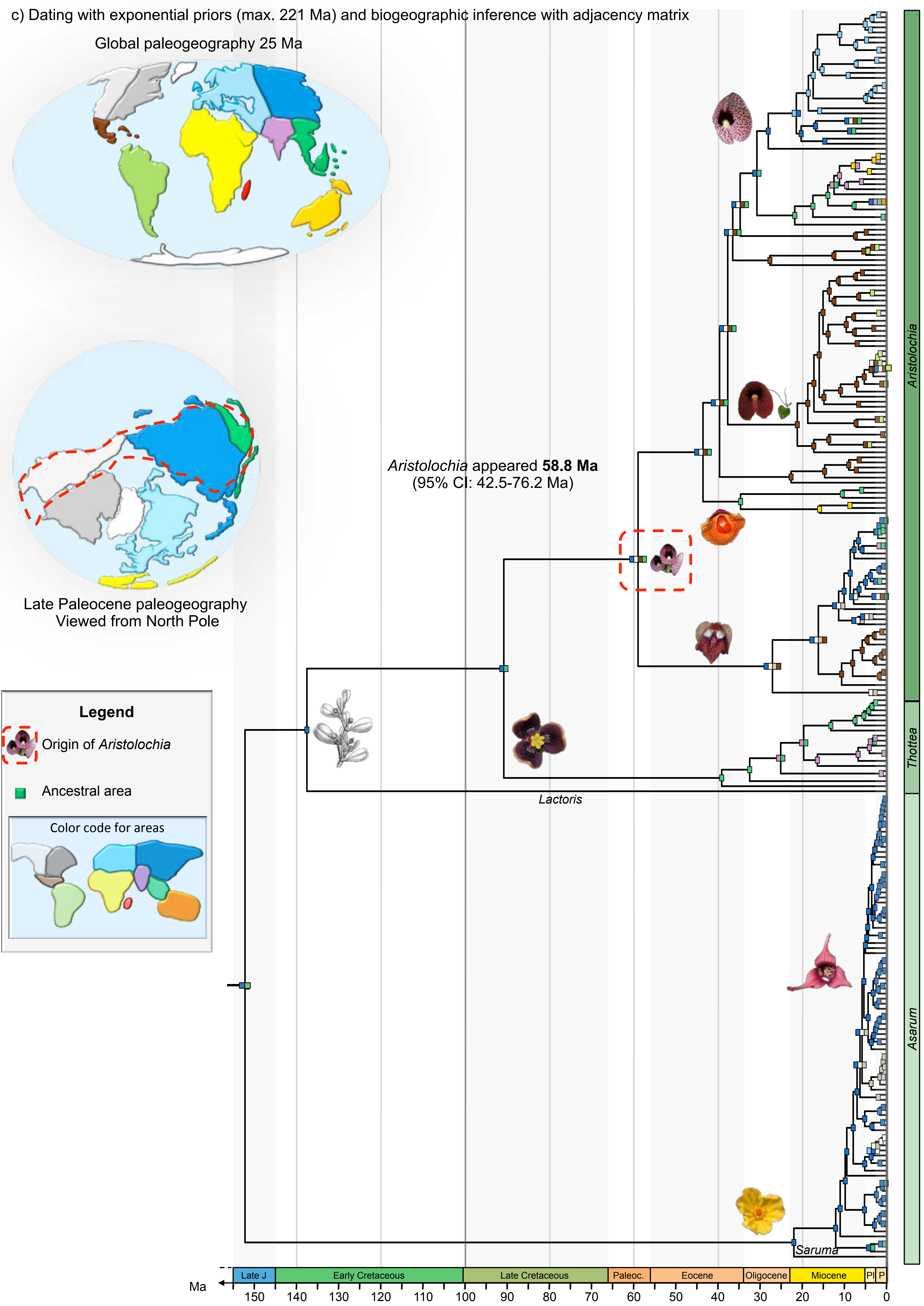
c) Dating with exponential priors (max. 221 Ma) and biogeographic inference with adjacency matrix
Global paleogeography 25 Ma
Late Paleocene paleogeography
Viewed from North Pole
Aristolochia appeared 58.8 Ma
(95% CI: 42.5-76.2 Ma)
Legend
Origin of Aristolochia
Ancestral area
Color code for areas
Lactoris
Saruma
Aristolochia
Thottea
Asarum
Late J
Early Cretaceous
Late Cretaceous
Paleoc.
Eocene
Oligocene
Miocene
Pl
P
Ma
150
140
130
120
110
100
90
80
70
60
50
40
30
20
10
0

d) Dating with exponential priors (max. 221 Ma) and biogeographic inference with unconstrained matrix

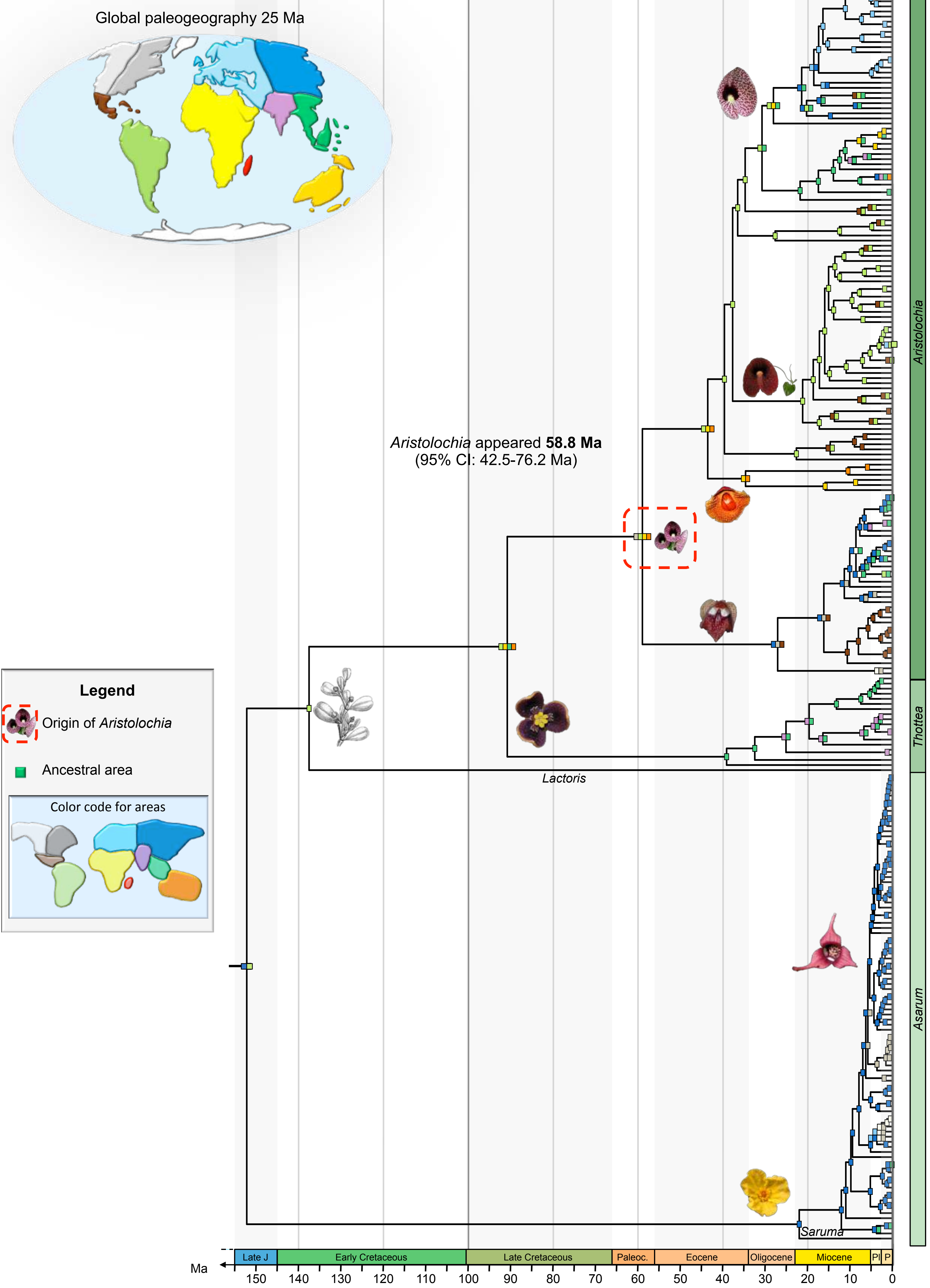

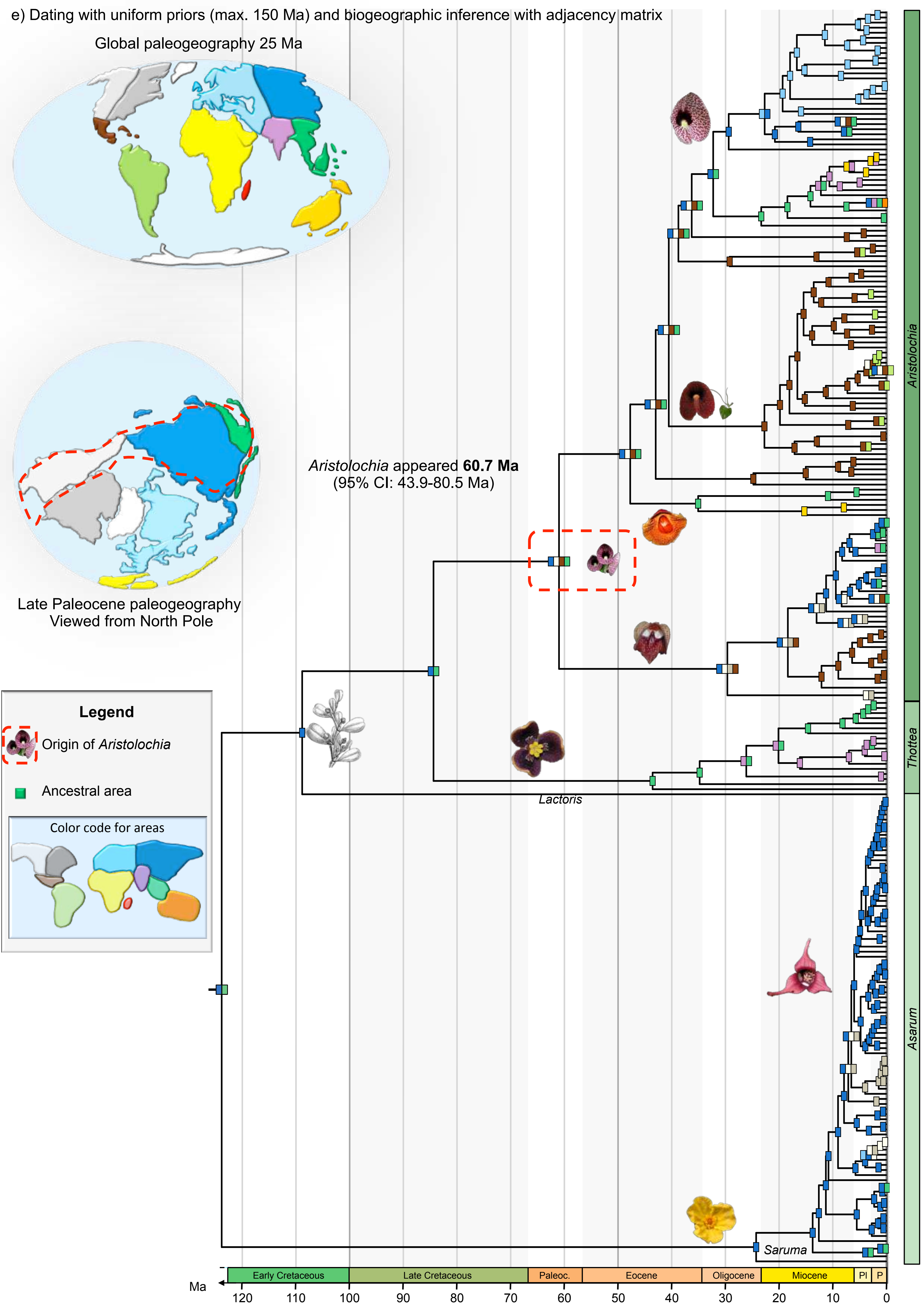

e) Dating with uniform priors (max. 150 Ma) and biogeographic inference with adjacency matrix
Global paleogeography 25 Ma
Late Paleocene paleogeography
Viewed from North Pole
Aristolochia appeared 60.7 Ma
(95% CI: 43.9-80.5 Ma)
Legend
Origin of Aristolochia
Ancestral area
Color code for areas
Lactoris
Saruma
Aristolochia
Thottea
Asarum
Early Cretaceous
Late Cretaceous
Paleoc.
Eocene
Oligocene
Miocene
Pl
P
Ma
120
110
100
90
80
70
60
50
40
30
20
10
0

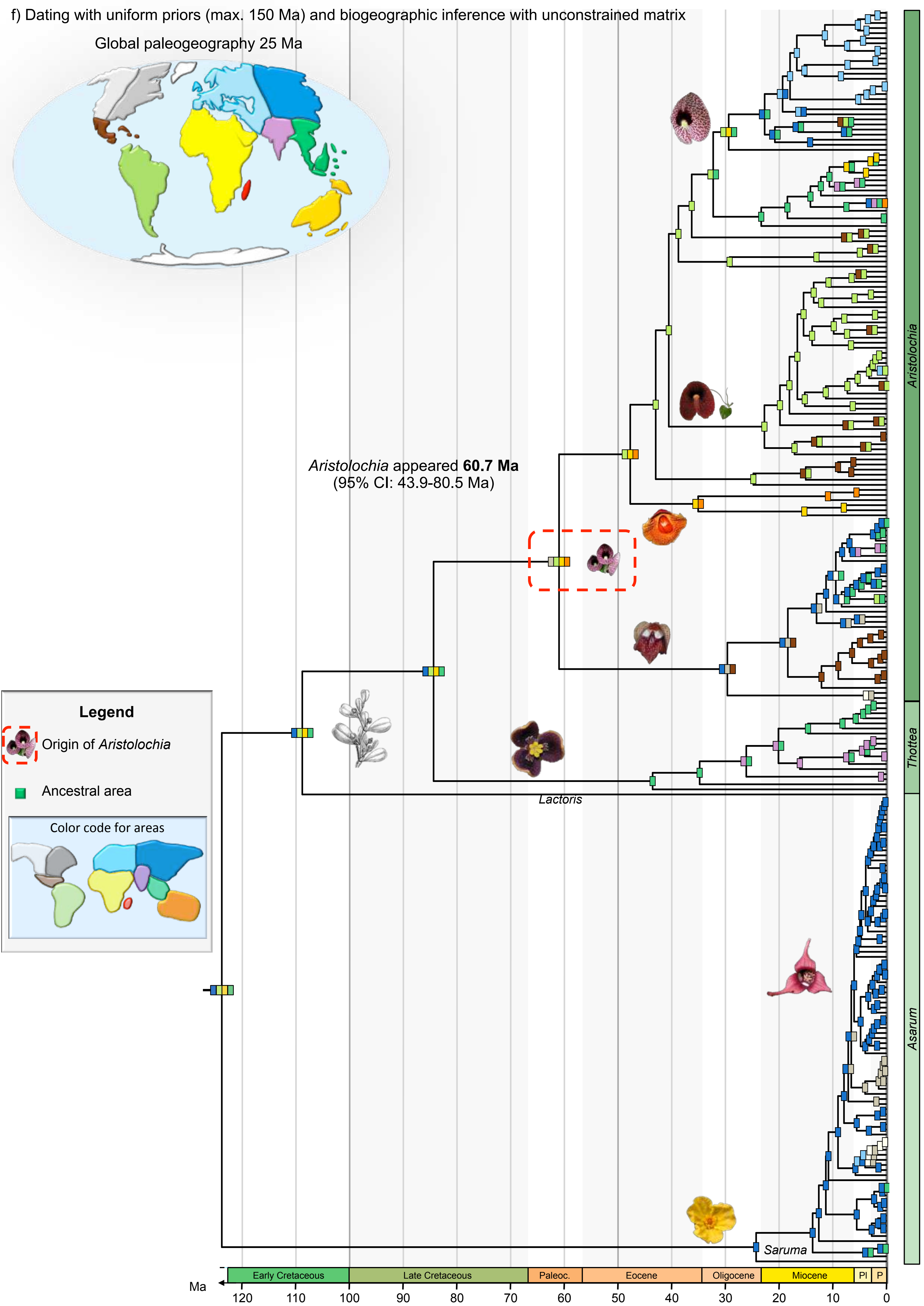

f) Dating with uniform priors (max. 150 Ma) and biogeographic inference with unconstrained matrix
Global paleogeography 25 Ma
Aristolochia appeared 60.7 Ma
(95% CI: 43.9-80.5 Ma)
Lactoris
Saruma
Aristolochia
Thottea
Asarum
Legend
Origin of Aristolochia
Ancestral area
Color code for areas
Early Cretaceous
Late Cretaceous
Paleoc.
Eocene
Oligocene
Miocene
Pl
P
Ma
120
110
100
90
80
70
60
50
40
30
20
10
0

g) Dating with uniform priors (max. 221 Ma) and biogeographic inference with adjacency matrix

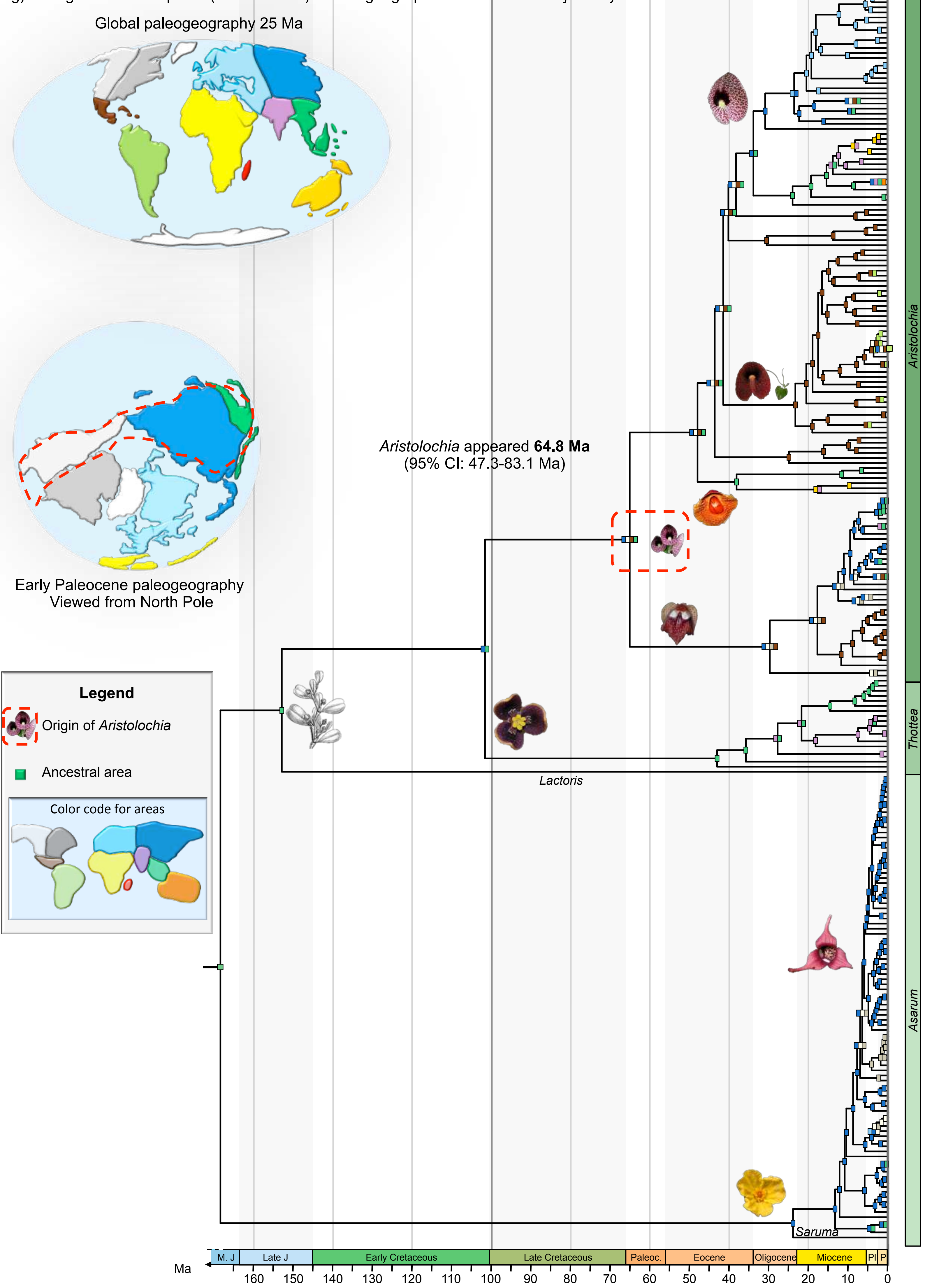

h) Dating with uniform priors (max. 221 Ma) and biogeographic inference with unconstrained matrix

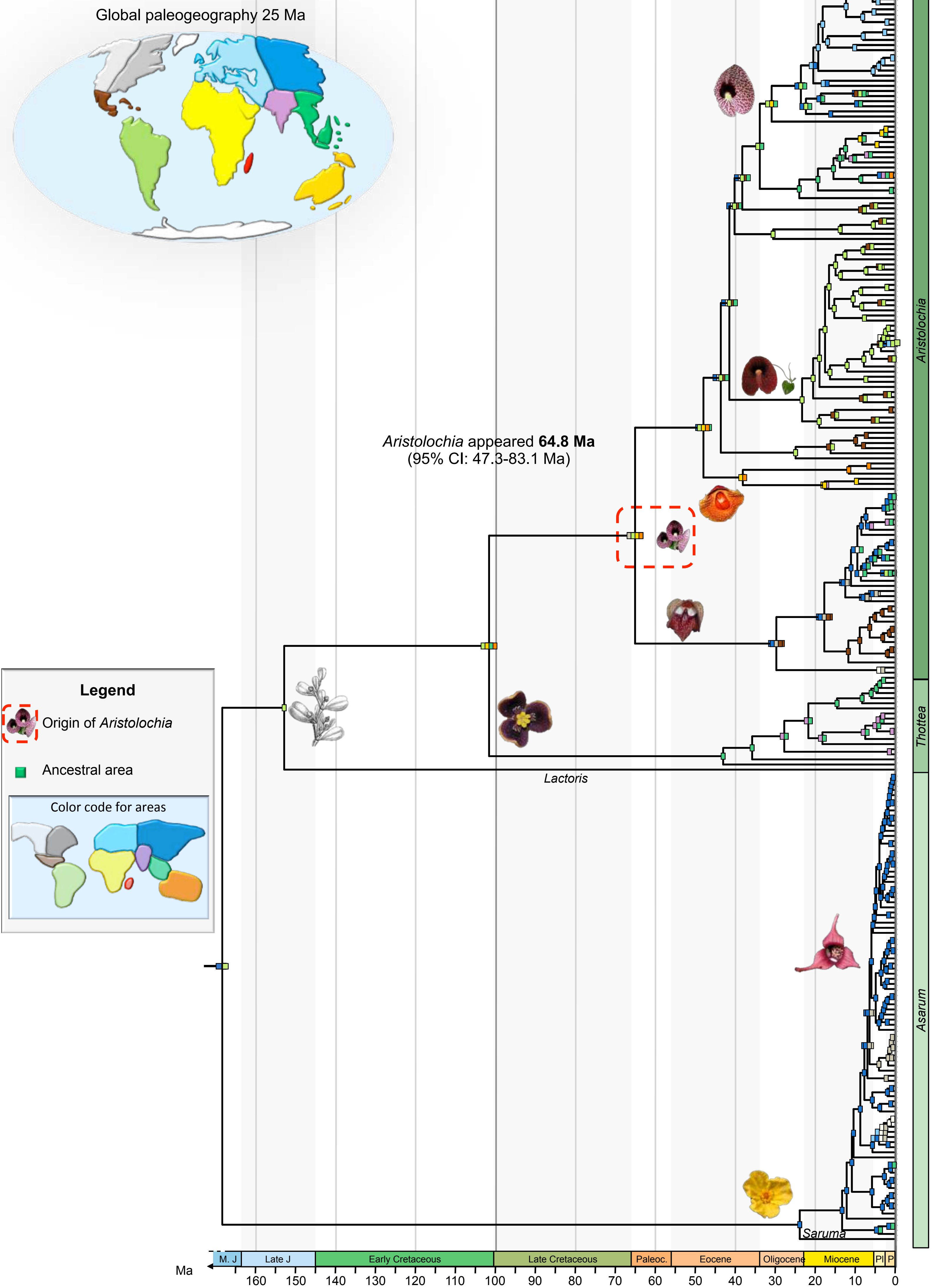

Supplementary figure 12. Trait-dependent diversification of Papilionidae linked to their host- plant. **a**, Bayesian inferences made with the full MuSSE model showed that speciation rates vary according to the host-plant trait. **b**, Boxplots showing the increase of diversification rates following host-plant shifts from the ancestral state (Aristolochiaceae). Only the species-poor swallowtail lineages feeding on Fabaceae, Zygophyllaceae and Magnoliaceae show decrease of diversification rates.

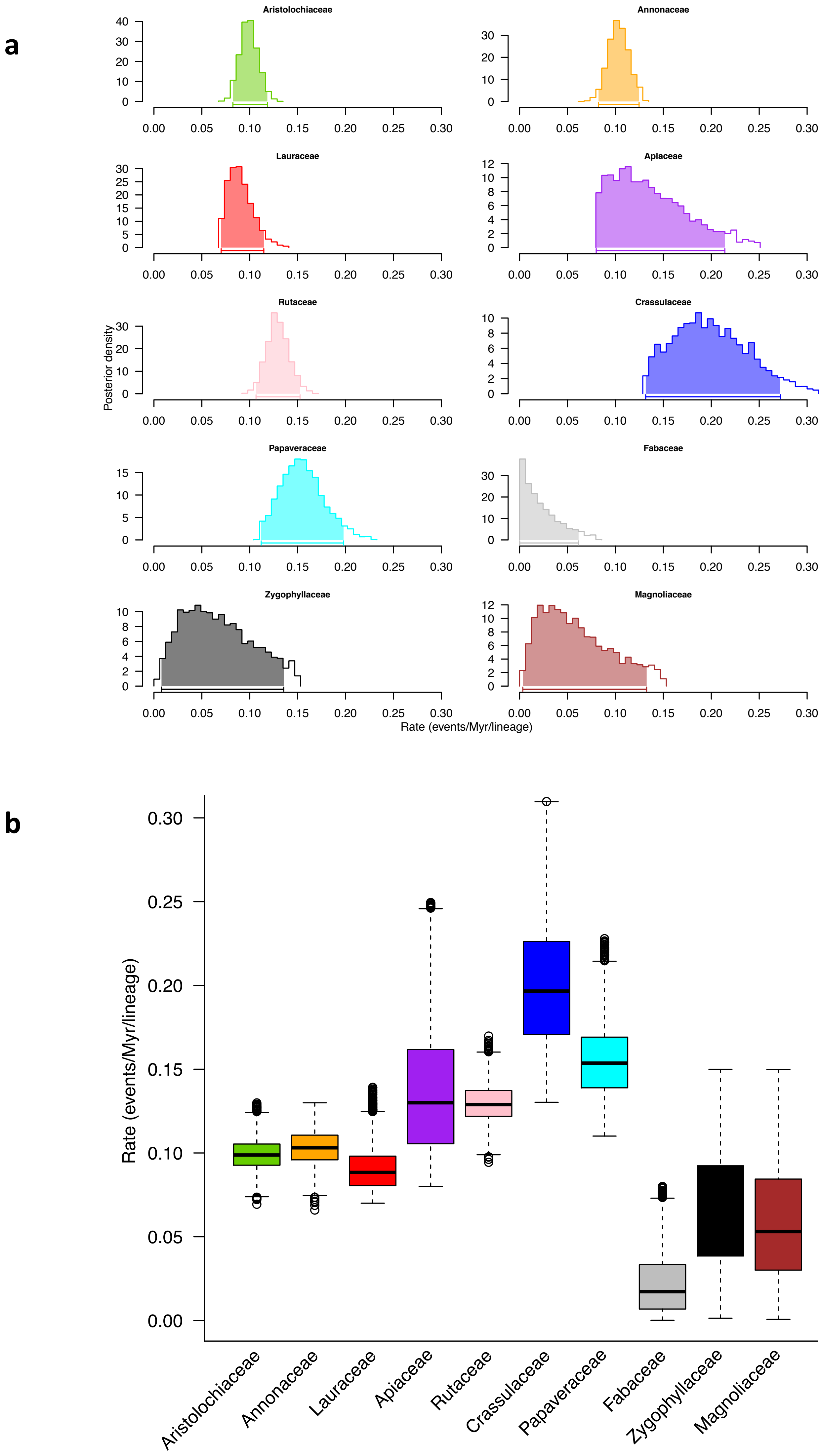
a
Aristolochiaceae
Annonaceae
Lauraceae
Apiaceae
Rutaceae
Crassulaceae
Papaveraceae
Fabaceae
Zygophyllaceae
Magnoliaceae
Posterior density
Rate (events/Myr/lineage)
b
Rate (events/Myr/lineage)
Aristolochiaceae
Annonaceae
Lauraceae
Apiaceae
Rutaceae
Crassulaceae
Papaveraceae
Fabaceae
Zygophyllaceae
Magnoliaceae

Supplementary figure 13. Time-dependent diversification of Papilionidae after shifting to new host-plants. Diversification is inferred with the RPANDA models, and the best-fit model is plotted showing rates through time for each clade. A model with increasing diversification over time best fits the Aristolochiaceae feeders. A model with a slowdown of diversification through time explained the diversification of Annonaceae feeders, Lauraceae feeders, and Papaveraceae feeders. A model with constant rates through time best fits the diversification of Apiaceae feeders, Crassulaceae feeders, and Rutaceae feeders. Solid lines indicate mean rates, and the shaded areas show 95% confidence intervals. K = Cretaceous, Pl = Pliocene, P = Pleistocene.

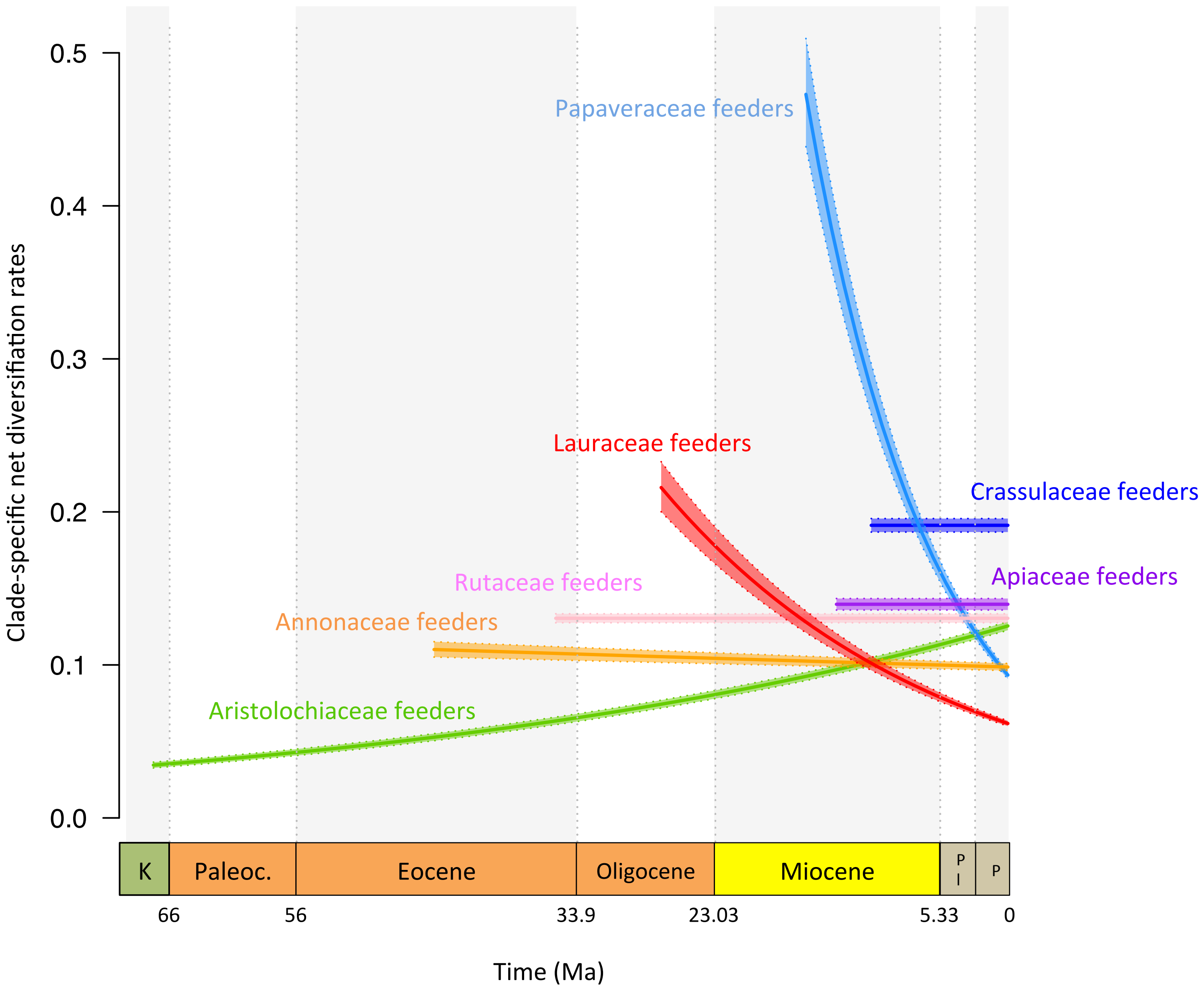

Papaveraceae feeders
Lauraceae feeders
Crassulaceae feeders
Apiaceae feeders
Rutaceae feeders
Annonaceae feeders
Aristolochiaceae feeders
0.5
0.4
0.3
0.2
0.1
0.0
Clade-specific net diversifiation rates
K
Paleoc.
Eocene
Oligocene
Miocene
P l
P
66
56
33.9
23.03
5.33
0
Time (Ma)

Supplementary figure 14. Bayesian analysis of clade-specific and time-dependent diversification of Papilionidae obtained with BAMM. a, Phylorate plot showing that global diversification rates increase through time in Papilionidae with no significant rate shifts detected by BAMM (the inset plot indicates the posterior probability for the estimated number of shifts). b, Rate-through-time plots for selected swallowtail lineages feeding on distinct host-plant families. The results also show an overall diversification increase through time for each group of swallowtails. P = Palaeocene, E = Eocene, O = Oligocene, M = Miocene.

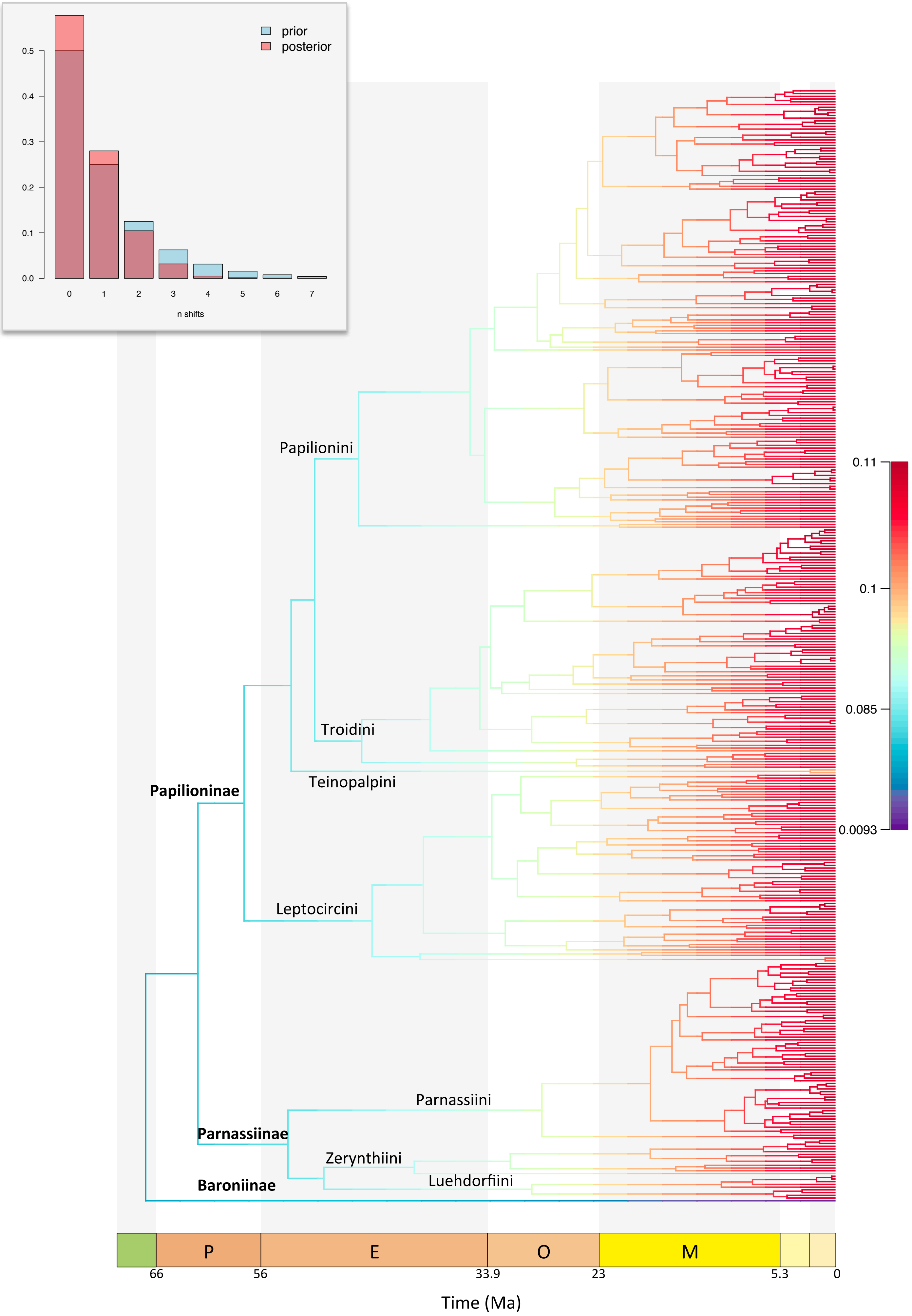

prior
posterior
0.5
0.4
0.3
0.2
0.1
0.0
0
1
2
3
4
5
6
7
n shifts
Papilionini
Troidini
Teinopalpini
Papilioninae
Leptocircini
Parnassiini
Parnassiinae
Zerynthiini
Luehdorfiini
Baroniinae
0.11
0.1
0.085
0.0093
P
E
O
M
66
56
33.9
23
5.3
0
Time (Ma)

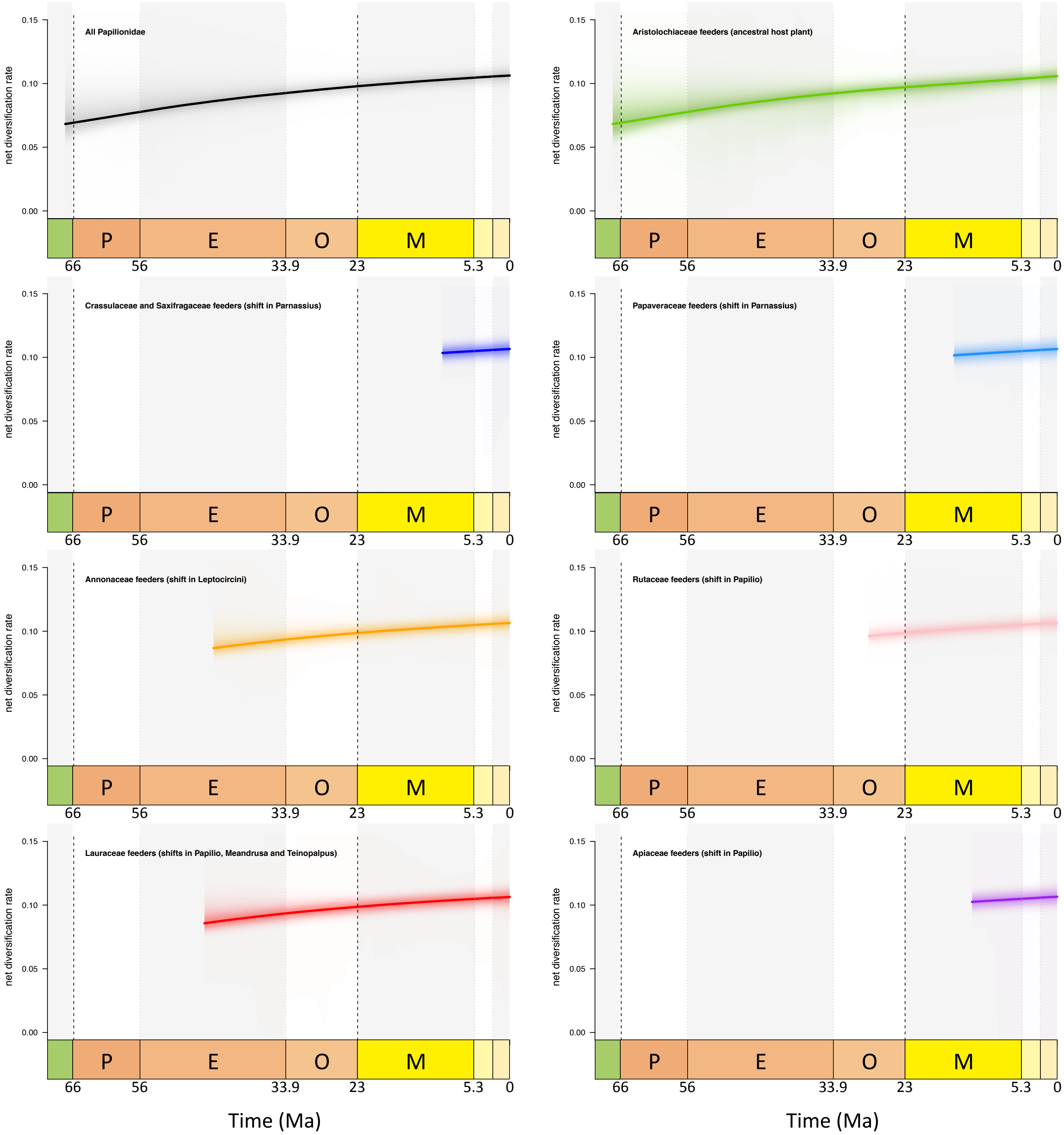
All Papilionidae
Aristolochiaceae feeders (ancestral host plant)
Crassulaceae and Saxifragaceae feeders (shift in Parnassius)
Papaveraceae feeders (shift in Parnassius)
Annonaceae feeders (shift in Leptocircini)
Rutaceae feeders (shift in Papilio)
Lauraceae feeders (shifts in Papilio, Meandrusa and Teinopalpus)
Apiaceae feeders (shift in Papilio)
net diversification rate
0.15
0.10
0.05
0.00
P
E
O
M
66
56
33.9
23
5.3
0
Time (Ma)

Supplementary figure 15. Bayesian analysis of branch-specific and time-dependent diversification of Papilionidae obtained with RevBayes. The median rates of diversification are plotted along each branch of the phylogeny, which shows a global increase of diversification rates through time in Papilionidae. Contrary to BAMM, this approach detected shifts in diversification rates in particular within the genera *Parnassius* and *Papilio* that have both shifted to new host-plant families. Mean posterior branch-specific rates are indicated in the scale bar on the topleft. P = Palaeocene, E = Eocene, O = Oligocene, M = Miocene. Pictures of butterflies made by Fabien Condamine.

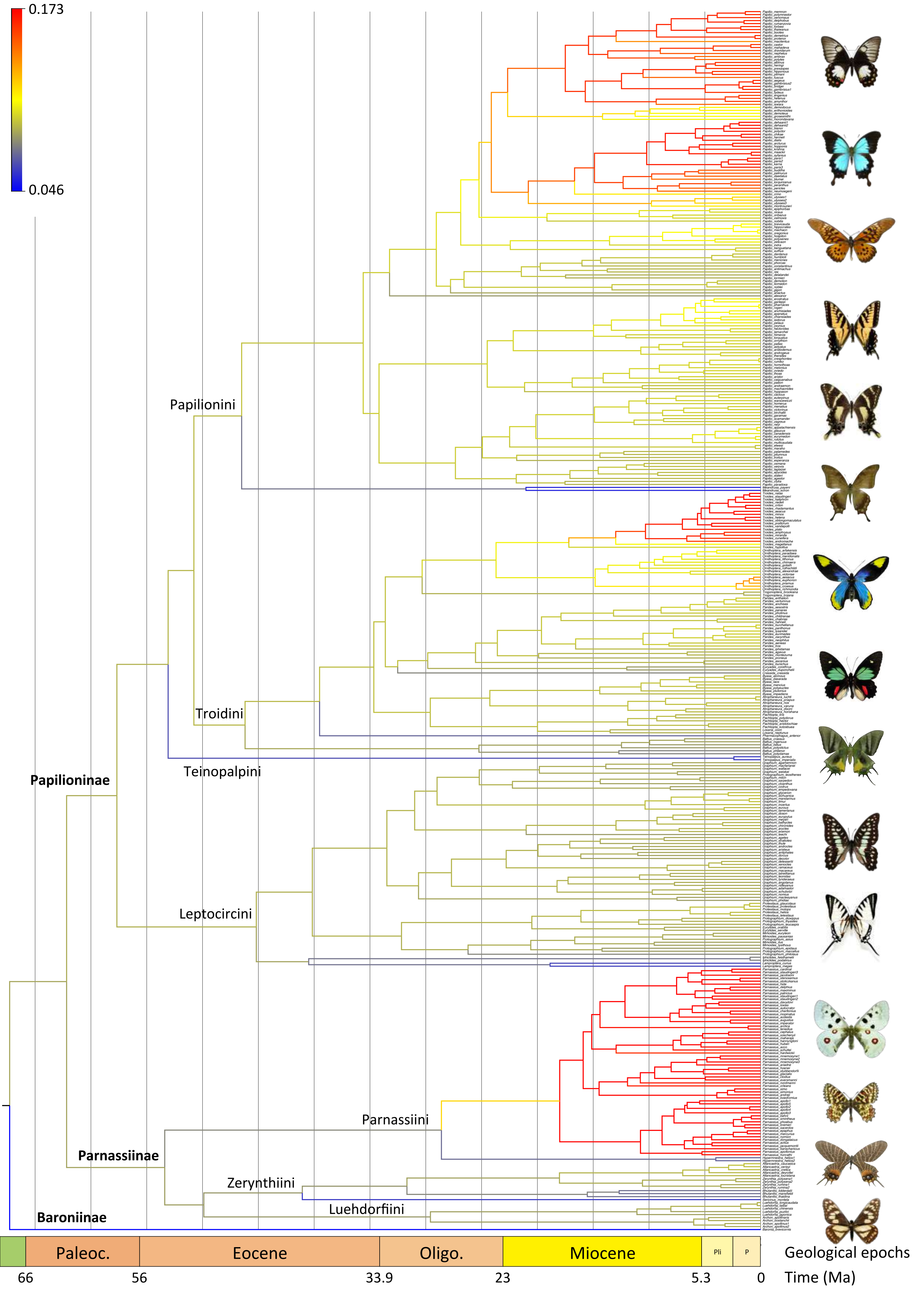
0.173
0.046
Papilionini
Troidini
Teinopalpini
Papilioninae
Leptocircini
Parnassiini
Parnassiinae
Zerynthiini
Luehdorfiini
Baroniinae
Paleoc.
Eocene
Oligo.
Miocene
Pli
P
Geological epochs
66
56
33.9
23
5.3
0
Time (Ma)

Supplementary figure 16. Bayesian analysis of episodic diversification of Papilionidae obtained with CoMET. The four plots represent speciation, extinction, net diversification, and relative extinction rates through time for the whole family. The result indicates a global increase of diversification rates over time, notably starting ~40 Ma. P = Palaeocene, E = Eocene, O = Oligocene, M = Miocene.

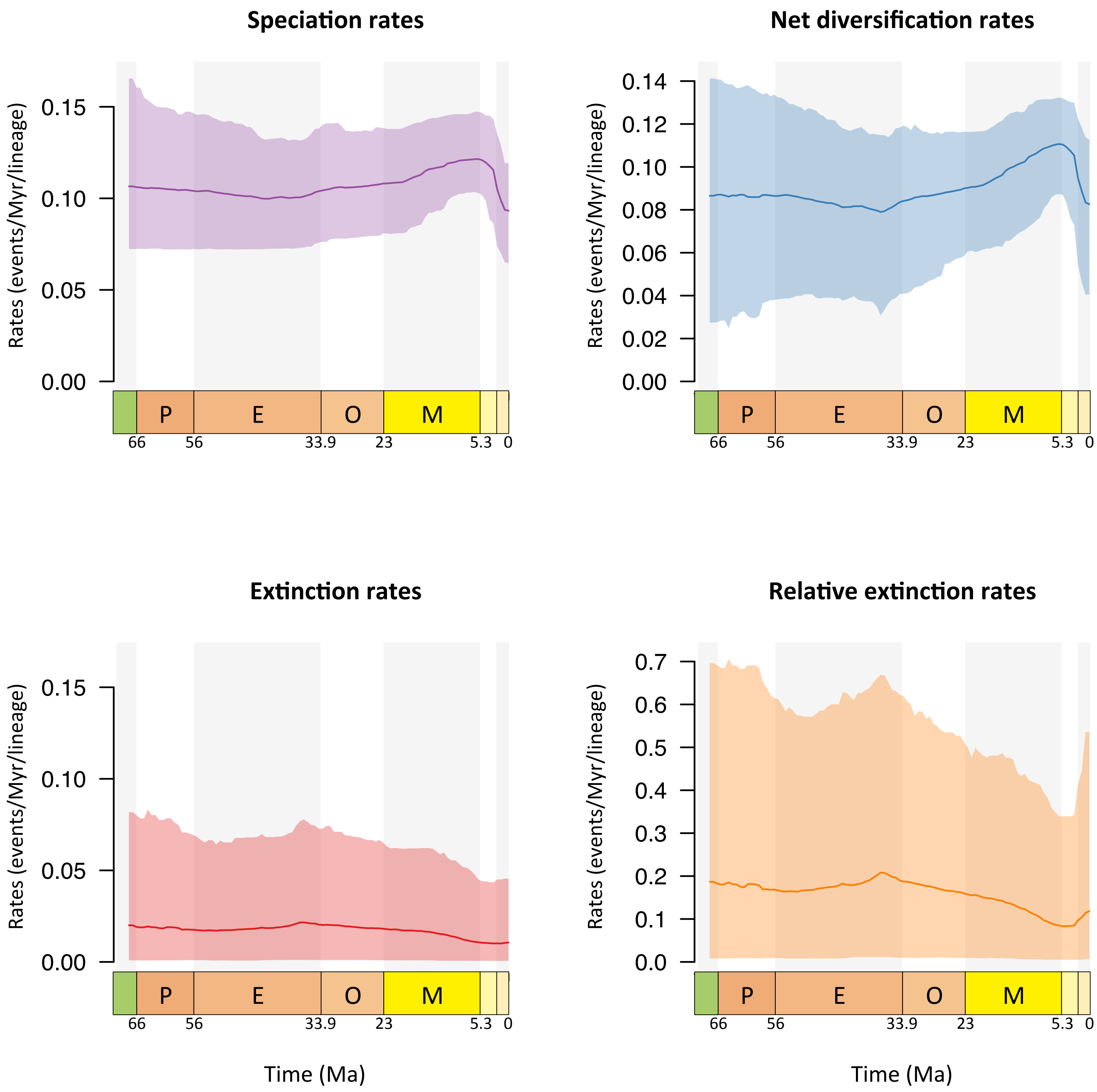

Speciation rates
Net diversification rates
Extinction rates
Relative extinction rates
Rates (events/Myr/lineage)
0.15
0.10
0.05
0.00
0.14
0.12
0.10
0.08
0.06
0.04
0.02
0.00
0.7
0.6
0.5
0.4
0.3
0.2
0.1
0.0
P
E
O
M
66
56
33.9
23
5.3
0
Time (Ma)

Supplementary figure 17. Number of host- plants consumed through time by Papilionidae. Using the estimation of ancestral host-plant preferences (Supplementary Fig. 4), we plotted the time at which a new host-plant family was colonised. This result shows that the swallowtail butterflies have a steady increase in the number of host families consumed over time. This ecological diversification can be paralleled with the global increase in diversification rates estimated by birth-death models (Supplementary Figs. 13-16). K = Cretaceous, Pl = Pliocene, P = Pleistocene.

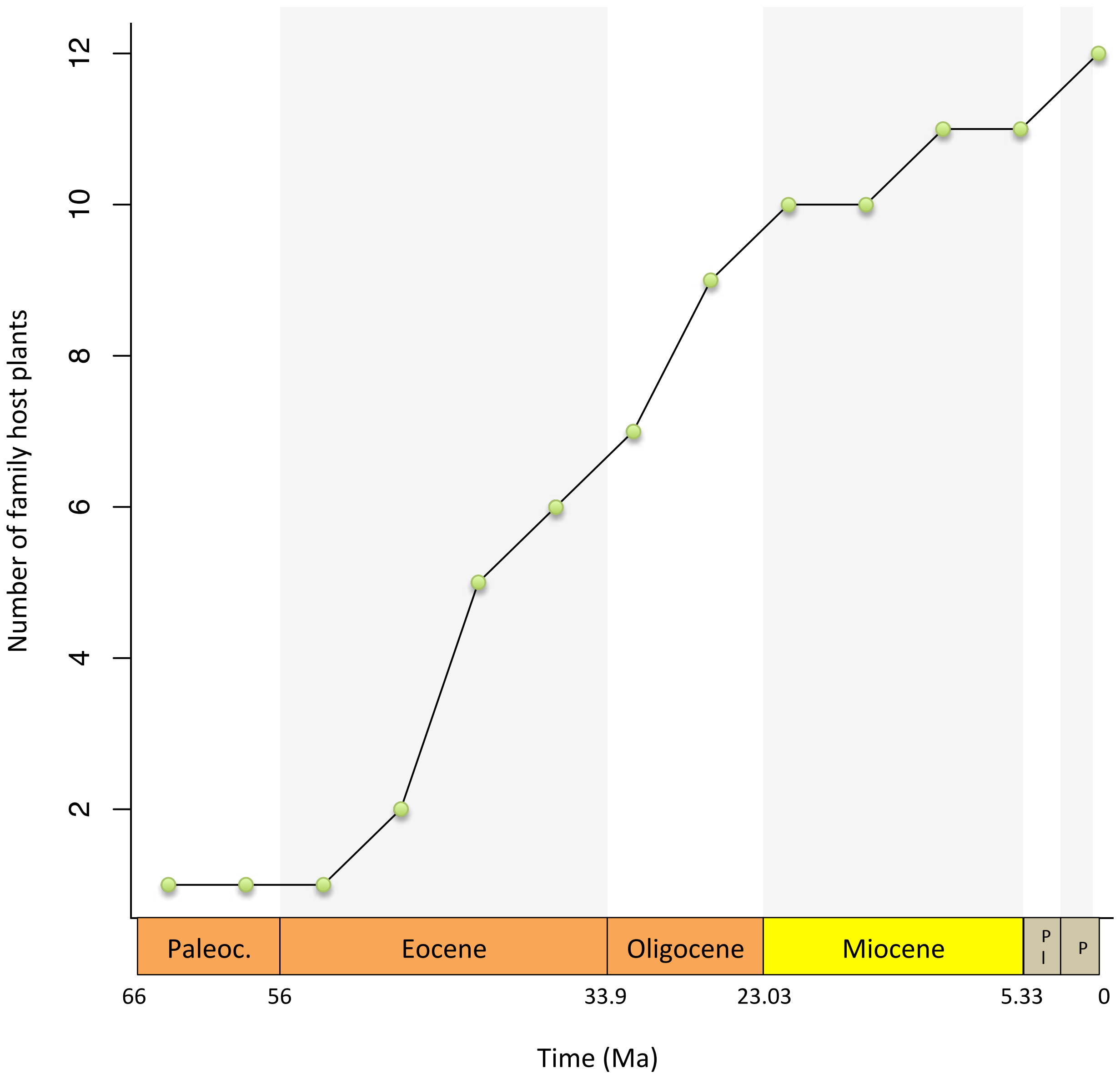

Number of family host plants
12
10
8
6
4
2
Paleoc.
Eocene
Oligocene
Miocene
P
l
P
66
56
33.9
23.03
5.33
0
Time (Ma)

Supplementary figure 18. Genus-level phylogenomic tree of Papilionidae showing the 14 selected branches with host-plant shifts and the 14 selected branches without host-plant shifts (control branches). The selection of these branches is based on the estimation of ancestral state models using the species-level phylogenies and current host-plant preferences (Supplementary Figs. 4, 5).

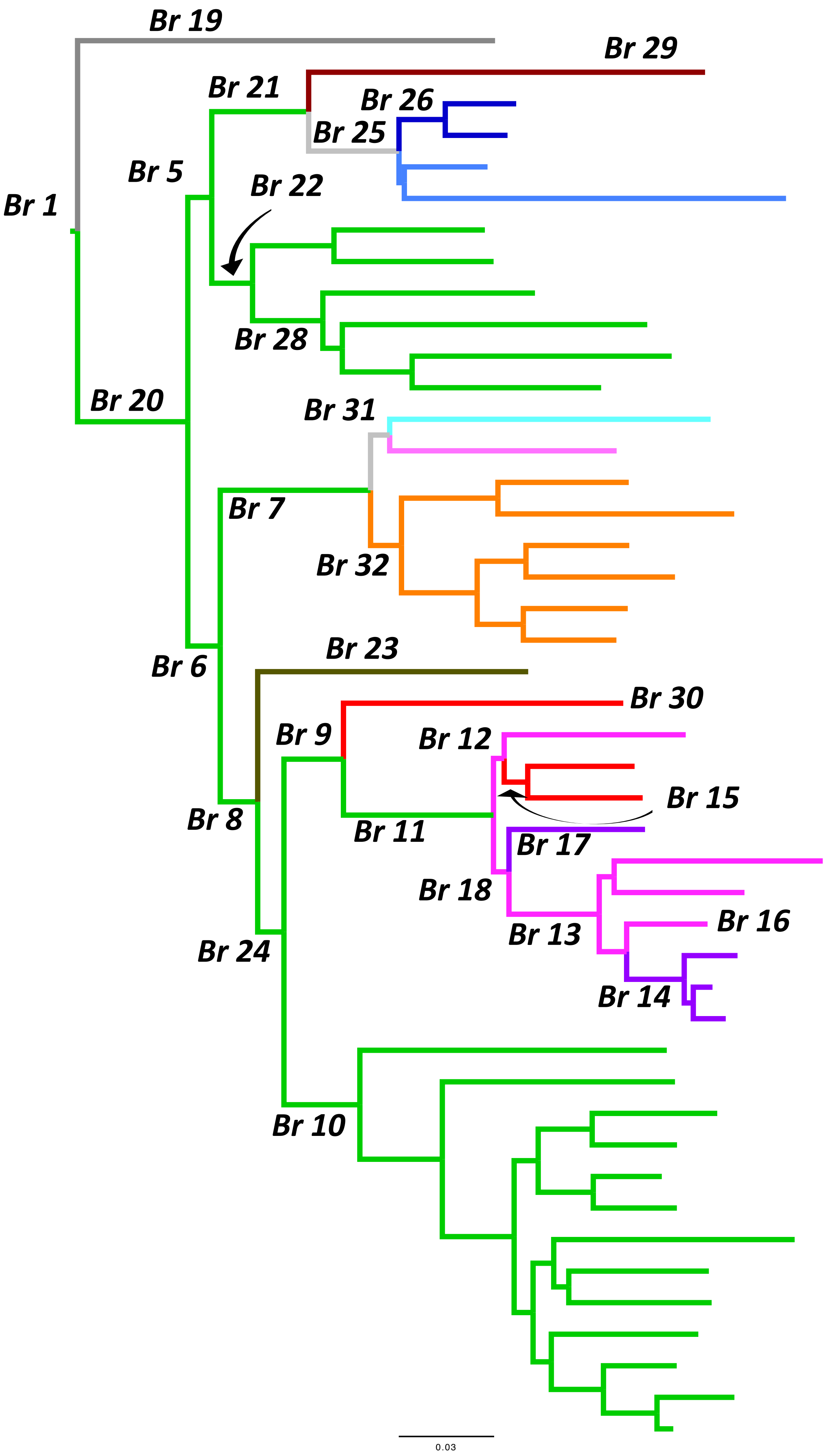
Br 19
Br 29
Br 21
Br 26
Br 25
Br 5
Br 22
Br 1
Br 28
Br 20
Br 31
Br 7
Br 32
Br 23
Br 6
Br 30
Br 9
Br 12
Br 15
Br 8
Br 11
Br 17
Br 18
Br 13
Br 16
Br 24
Br 14
Br 10
0.03

Branch ID	***Plant shift***	***Climate shift***	***Plant and climate shifts***
Branch1	Host-plant shift	No shift	Host shift only
Branch5	No shift	No shift	No shift
Branch6	No shift	No shift	No shift
Branch7	Host-plant shift	No shift	Host shift only
Branch8	No shift	No shift	No shift
Branch9	Host-plant shift	No shift	Host shift only
Branch10	No shift	No shift	No shift
Branch11	Host-plant shift	No shift	Host shift only
Branch12	No shift	No shift	No shift
Branch13	No shift	No shift	No shift
Branch14	Host-plant shift	Climate shift	Host and climate shifts
Branch15	Host-plant shift	Climate shift	Host and climate shifts
Branch16	No shift	No shift	No shift
Branch17	Host-plant shift	Climate shift	Host and climate shifts
Branch18	No shift	No shift	No shift
Branch19	Host-plant shift	No shift	Host shift only
Branch20	No shift	No shift	No shift
Branch21	Host-plant shift	No shift	Host shift only
Branch22	No shift	No shift	No shift
Branch23	Host-plant shift	No shift	Host shift only
Branch24	No shift	No shift	No shift
Branch25	Host-plant shift	Climate shift	Host and climate shifts
Branch26	No shift	No shift	No shift
Branch28	No shift	No shift	No shift
Branch29	Host-plant shift	No shift	Host shift only
Branch30	Host-plant shift	No shift	Host shift only
Branch31	Host-plant shift	Climate shift	Host and climate shifts
Branch32	No shift	No shift	No shift

Supplementary figure 19. a, Genus-level phylogenomic tree displaying branches with and without host-plant shifts, on which genome-wide analyses of molecular evolution are performed. **b,** Proportion of genes under positive selection (dN/dS > 1) for swallowtail lineages shifting to new host-plant families (n=14, green) or not (n=14, grey). **c,** Proportion of genes under positive selection for swallowtail lineages undergoing climate shifts (n=5, orange) or not (n=23, grey). **d,** Proportion of genes under positive selection for swallowtail lineages shifting to new host-plants (n=9, green), shifting both host-plant and climate (n=5, blue) or not (n=14, grey). The proportion of genes was estimated with *Dataset 1* (520 genes). This demonstrates genome-wide signatures of adaptations in swallowtail lineages shifting to new host-plant families. Genes under positive selection did not contain over- or under-represented functional GO categories (Supplementary data file 2). Wilcoxon rank-sum test: n.s. = not significant ($P > 0.05$), * = $P \leq 0.05$, ** = $P \leq 0.01$. Pictures and icons made by Fabien Condamine.

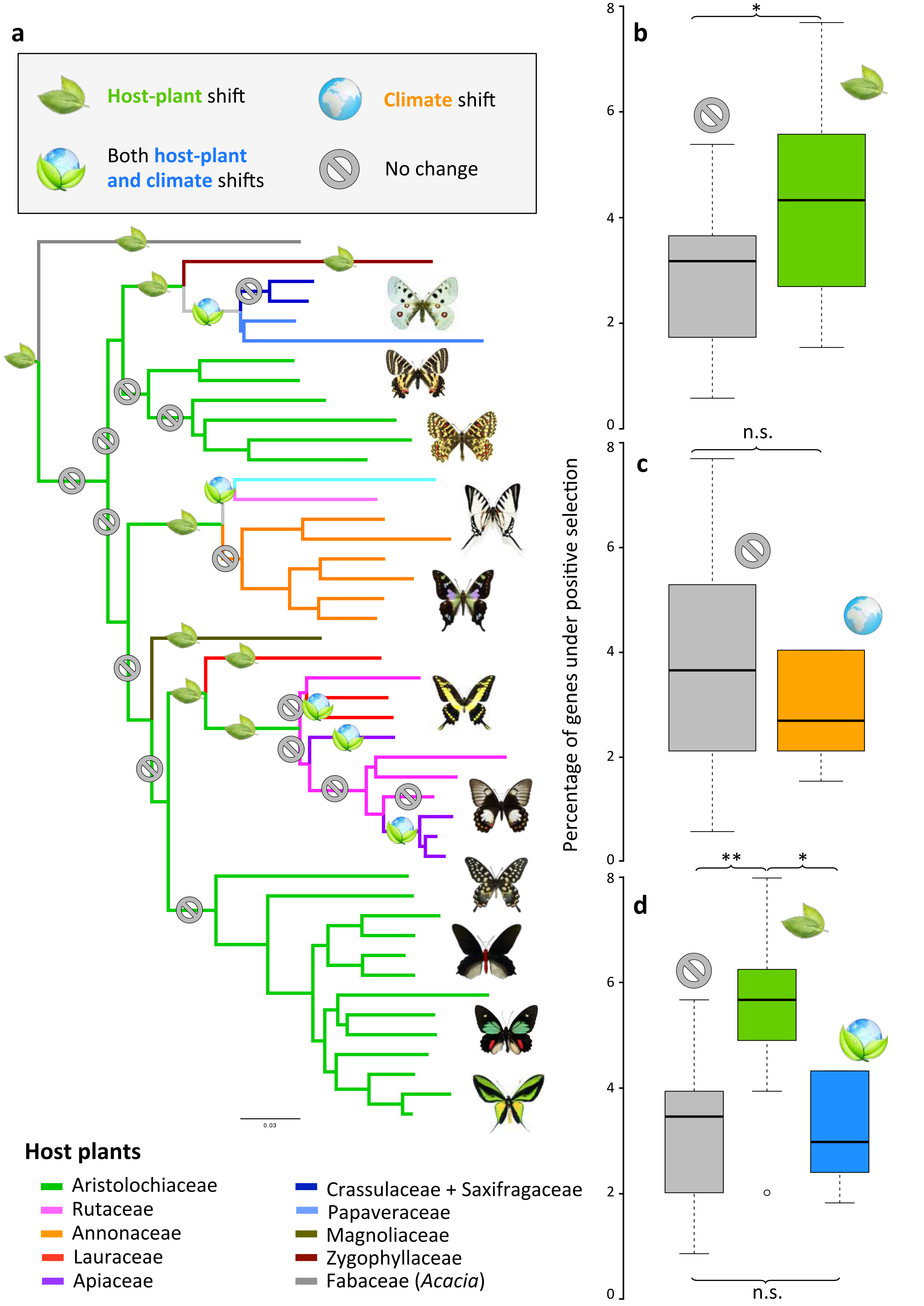
a
Host-plant shift
Climate shift
Both host-plant and climate shifts
No change
0.03
Host plants
Aristolochiaceae
Rutaceae
Annonaceae
Lauraceae
Apiaceae
Crassulaceae + Saxifragaceae
Papaveraceae
Magnoliaceae
Zygophyllaceae
Fabaceae (Acacia)
b
c
d
Percentage of genes under positive selection
*
n.s.
**
*
n.s.
0
2
4
6
8

Supplementary figure 20. Violin plots of the percentage of missing data ("N" or "-") and proportion of GC at third codon position (GC3) in alignment where positive selection have been detected ("Yes") and positive selection have not been detected ("No"). Panels a and b are *Dataset 1* with 520 genes, and panels c and d are *Dataset 2* with 1,533 genes.

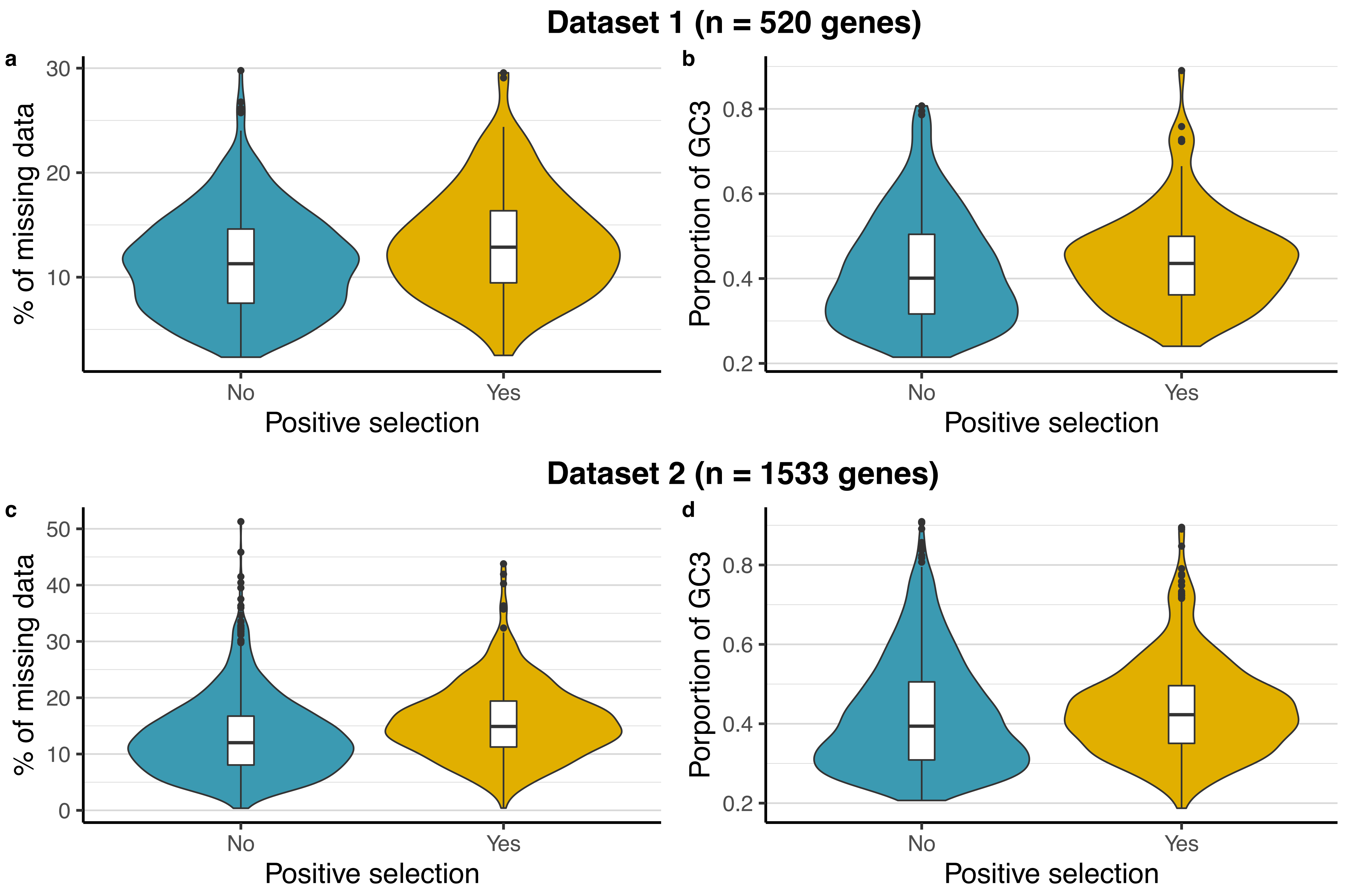

Dataset 1 (n = 520 genes)
a
% of missing data
30
20
10
No
Yes
Positive selection
b
Porportion of GC3
0.8
0.6
0.4
0.2
No
Yes
Positive selection
Dataset 2 (n = 1533 genes)
c
% of missing data
50
40
30
20
10
0
No
Yes
Positive selection
d
Porportion of GC3
0.8
0.6
0.4
0.2
No
Yes
Positive selection

Supplementary figure 21. The percentage of missing data ("N" or "-") per genes across species computed for *Dataset 1* and *Dataset 2*.

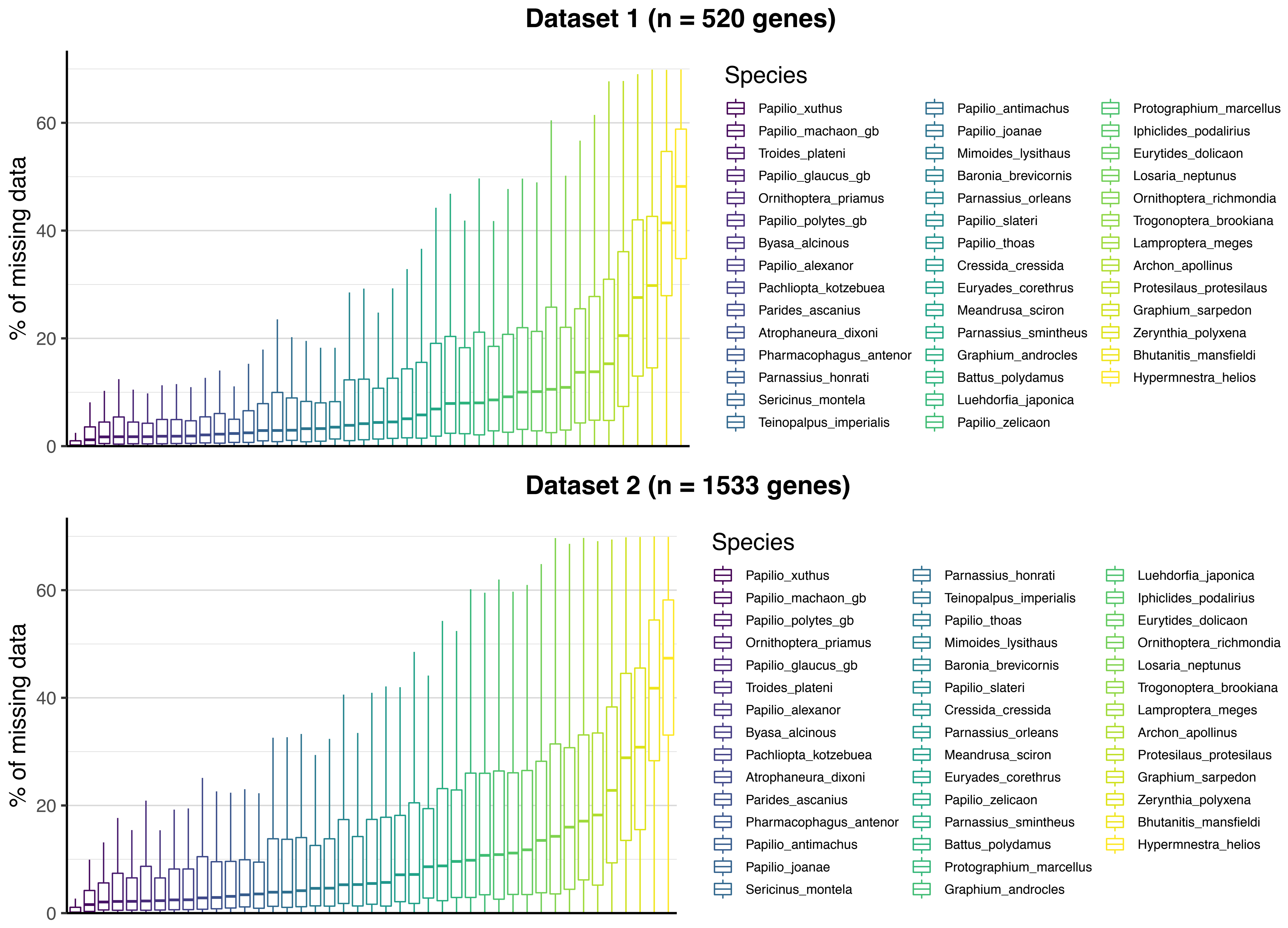
Dataset 1 (n = 520 genes)
% of missing data
0
20
40
60
Species
Papilio_xuthus
Papilio_machaon_gb
Troides_plateni
Papilio_glaucus_gb
Ornithoptera_priamus
Papilio_polytes_gb
Byasa_alcinous
Papilio_alexanor
Pachliopta_kotzebuea
Parides_ascanius
Atrophaneura_dixoni
Pharmacophagus_antenor
Parnassius_honrati
Sericinus_montela
Teinopalpus_imperialis
Papilio_antimachus
Papilio_joanae
Mimoides_lysithaus
Baronia_brevicornis
Parnassius_orleans
Papilio_slateri
Papilio_thoas
Cressida_cressida
Euryades_corethrus
Meandrusa_sciron
Parnassius_smintheus
Graphium_androcles
Battus_polydamus
Luehdorfia_japonica
Papilio_zelicaon
Protographium_marcellus
Iphiclides_podalirius
Eurytides_dolicaon
Losaria_neptunus
Ornithoptera_richmondia
Trogonoptera_brookiana
Lamproptera_meges
Archon_apollinus
Protesilaus_protesilaus
Graphium_sarpedon
Zerynthia_polyxena
Bhutanitis_mansfieldi
Hypermnestra_helios
Dataset 2 (n = 1533 genes)
% of missing data
0
20
40
60
Species
Papilio_xuthus
Papilio_machaon_gb
Papilio_polytes_gb
Ornithoptera_priamus
Papilio_glaucus_gb
Troides_plateni
Papilio_alexanor
Byasa_alcinous
Pachliopta_kotzebuea
Atrophaneura_dixoni
Parides_ascanius
Pharmacophagus_antenor
Papilio_antimachus
Papilio_joanae
Sericinus_montela
Parnassius_honrati
Teinopalpus_imperialis
Papilio_thoas
Mimoides_lysithaus
Baronia_brevicornis
Papilio_slateri
Cressida_cressida
Parnassius_orleans
Meandrusa_sciron
Euryades_corethrus
Papilio_zelicaon
Parnassius_smintheus
Battus_polydamus
Protographium_marcellus
Graphium_androcles
Luehdorfia_japonica
Iphiclides_podalirius
Eurytides_dolicaon
Ornithoptera_richmondia
Losaria_neptunus
Trogonoptera_brookiana
Lamproptera_meges
Archon_apollinus
Protesilaus_protesilaus
Graphium_sarpedon
Zerynthia_polyxena
Bhutanitis_mansfieldi
Hypermnestra_helios

Supplementary figure 22. The percentage of missing data (“N” or “-”) per branch for the branches with (“Yes”, n = 14) and without (“No”, n = 14) host-plant shift. For a given branch, the percentage of missing data is the mean value of the species of a clade for which the branch is the root.

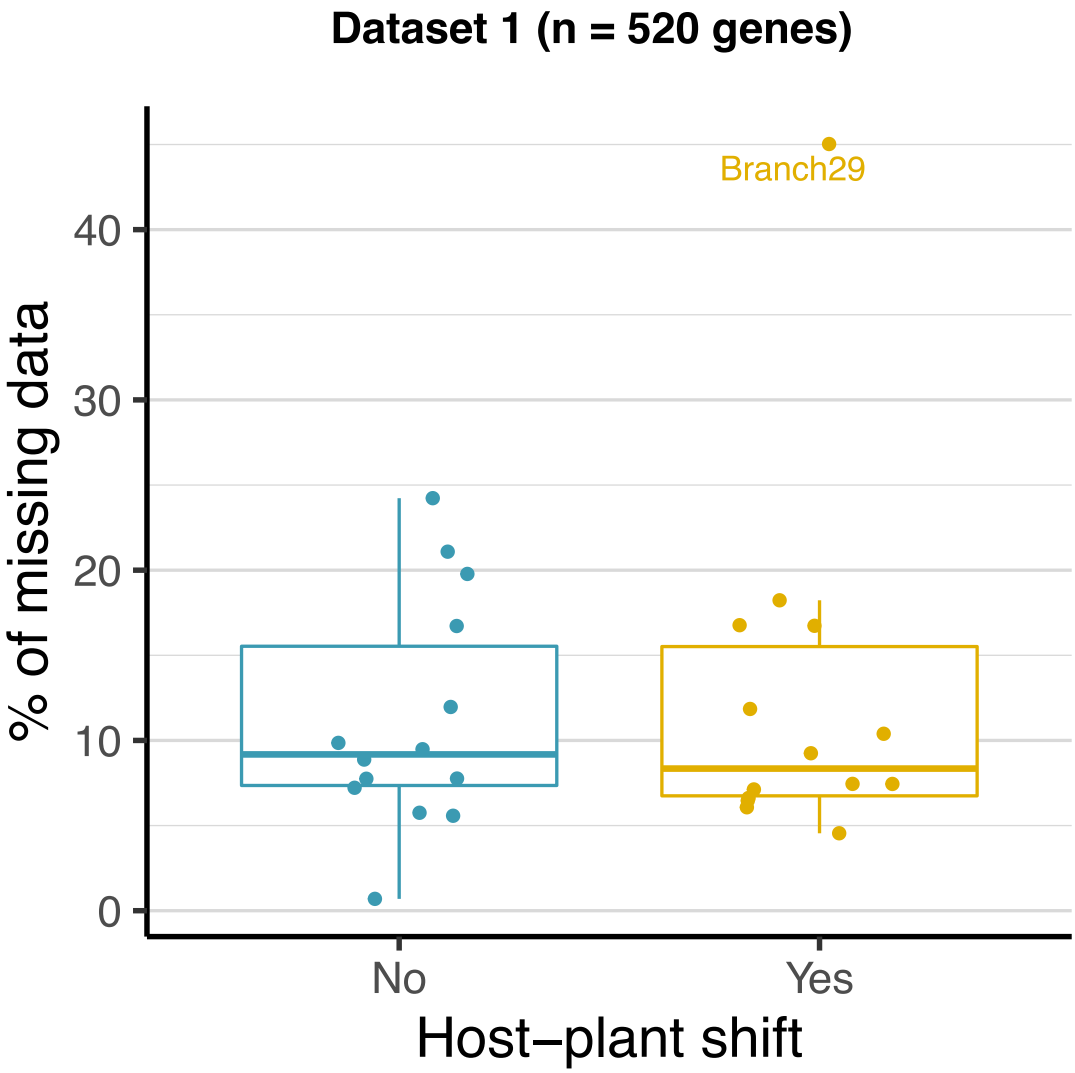

Dataset 1 (n = 520 genes)
Branch29
40
30
20
10
0
% of missing data
No
Yes
Host−plant shift

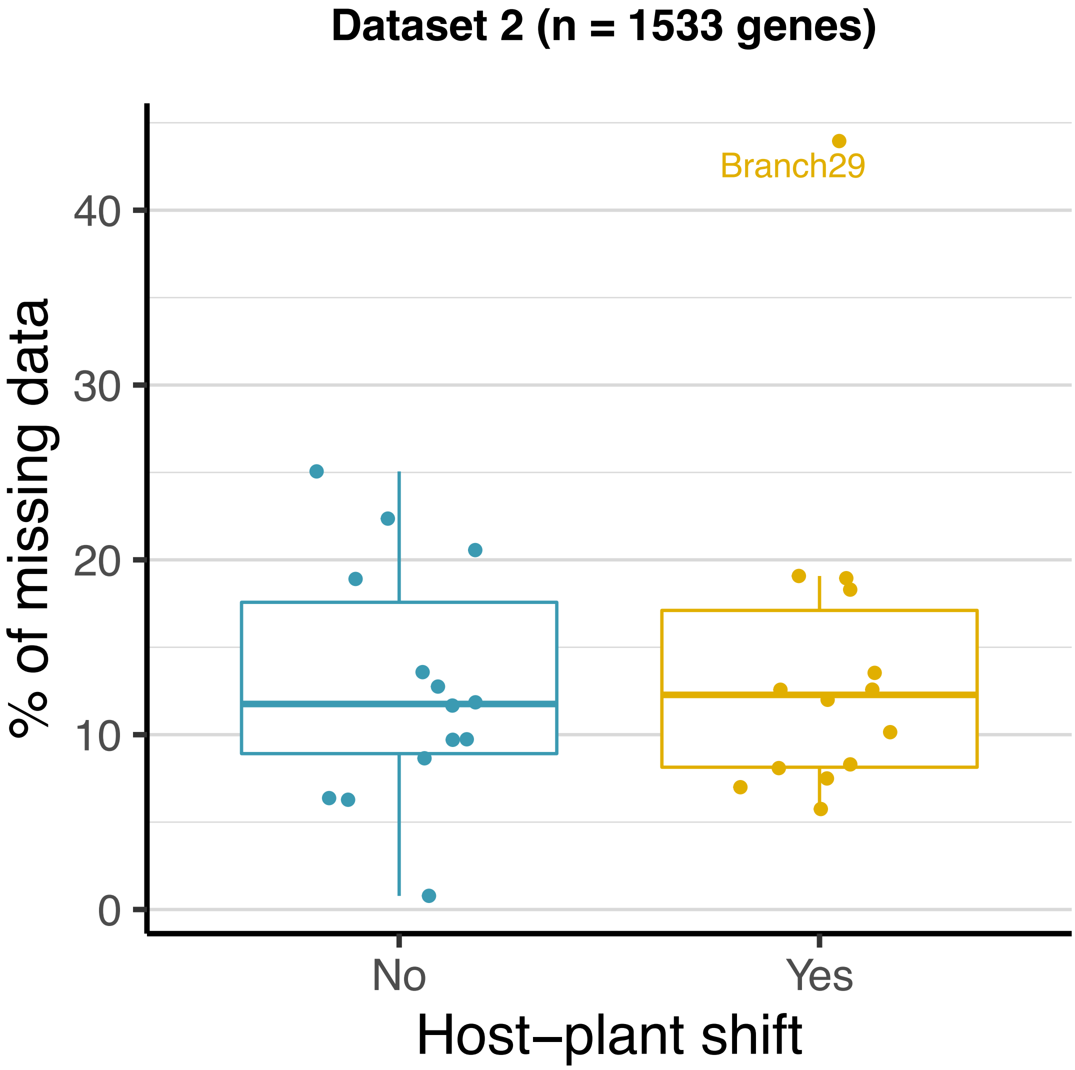

Dataset 2 (n = 1533 genes)
Branch29
40
30
20
10
0
% of missing data
No
Yes
Host−plant shift

Supplementary figure 23. Relationship between the percentage of missing data (“N” or “-”) and the number of positively selected genes per branch. For a given branch, the percentage of missing data is the mean value of the species of a clade for which the branch is the root.

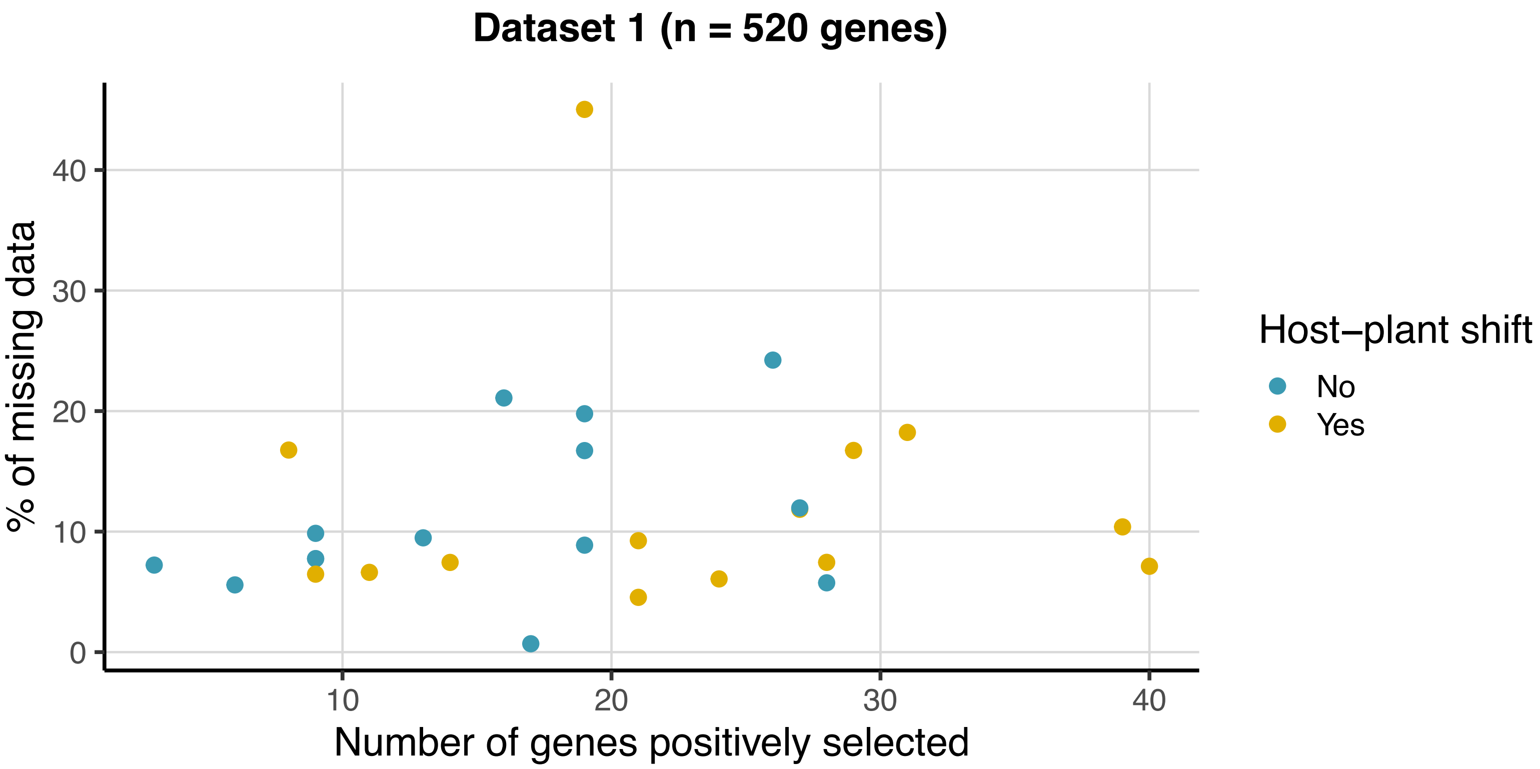
Dataset 1 (n = 520 genes)
% of missing data
0
10
20
30
40
10
20
30
40
Number of genes positively selected
Host−plant shift
No
Yes

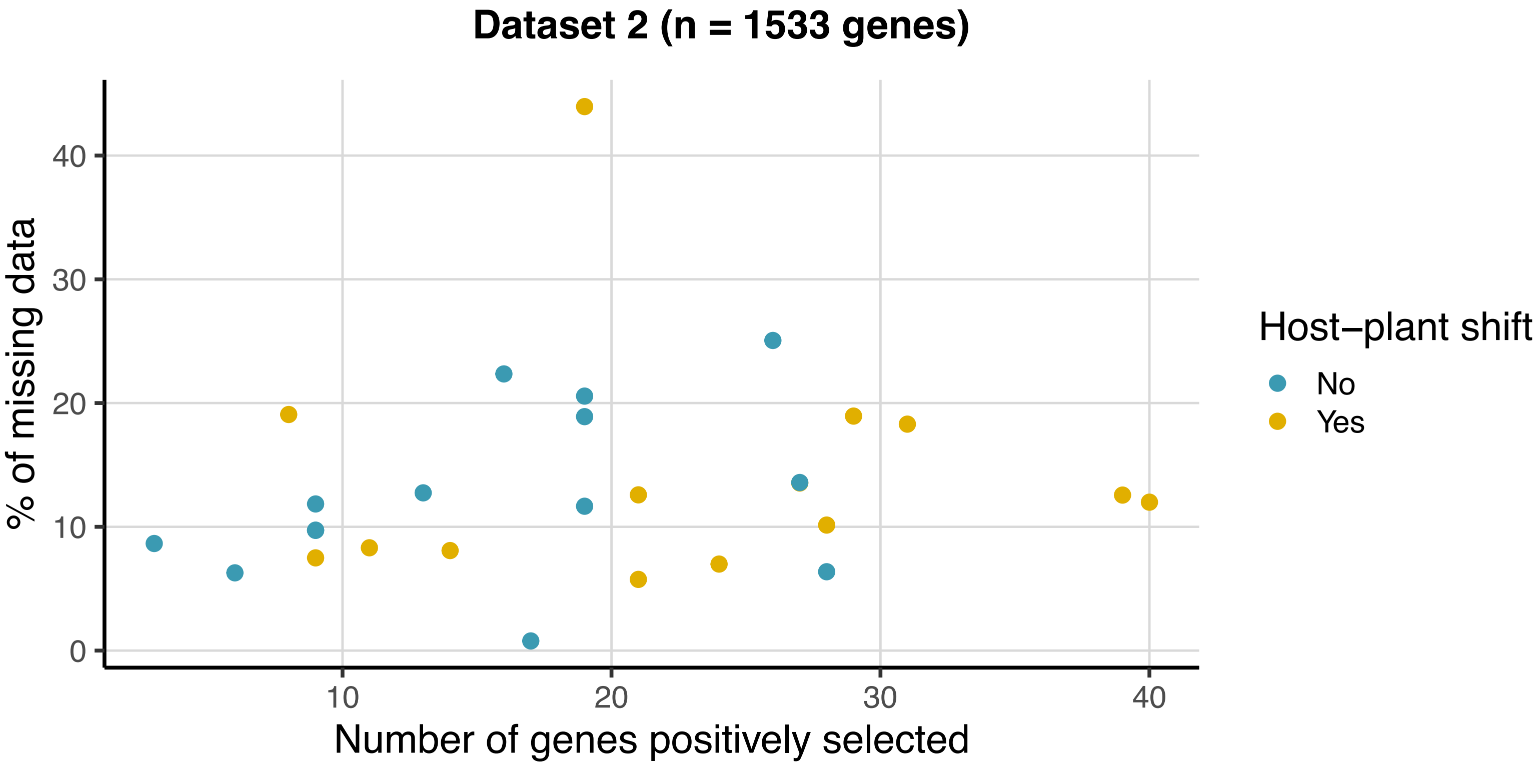
Dataset 2 (n = 1533 genes)
% of missing data
0
10
20
30
40
10
20
30
40
Number of genes positively selected
Host−plant shift
No
Yes

Supplementary figure 24. The percentage of GC at third codon position (GC3) per gene across species computed for *Dataset 1* and *Dataset 2*.

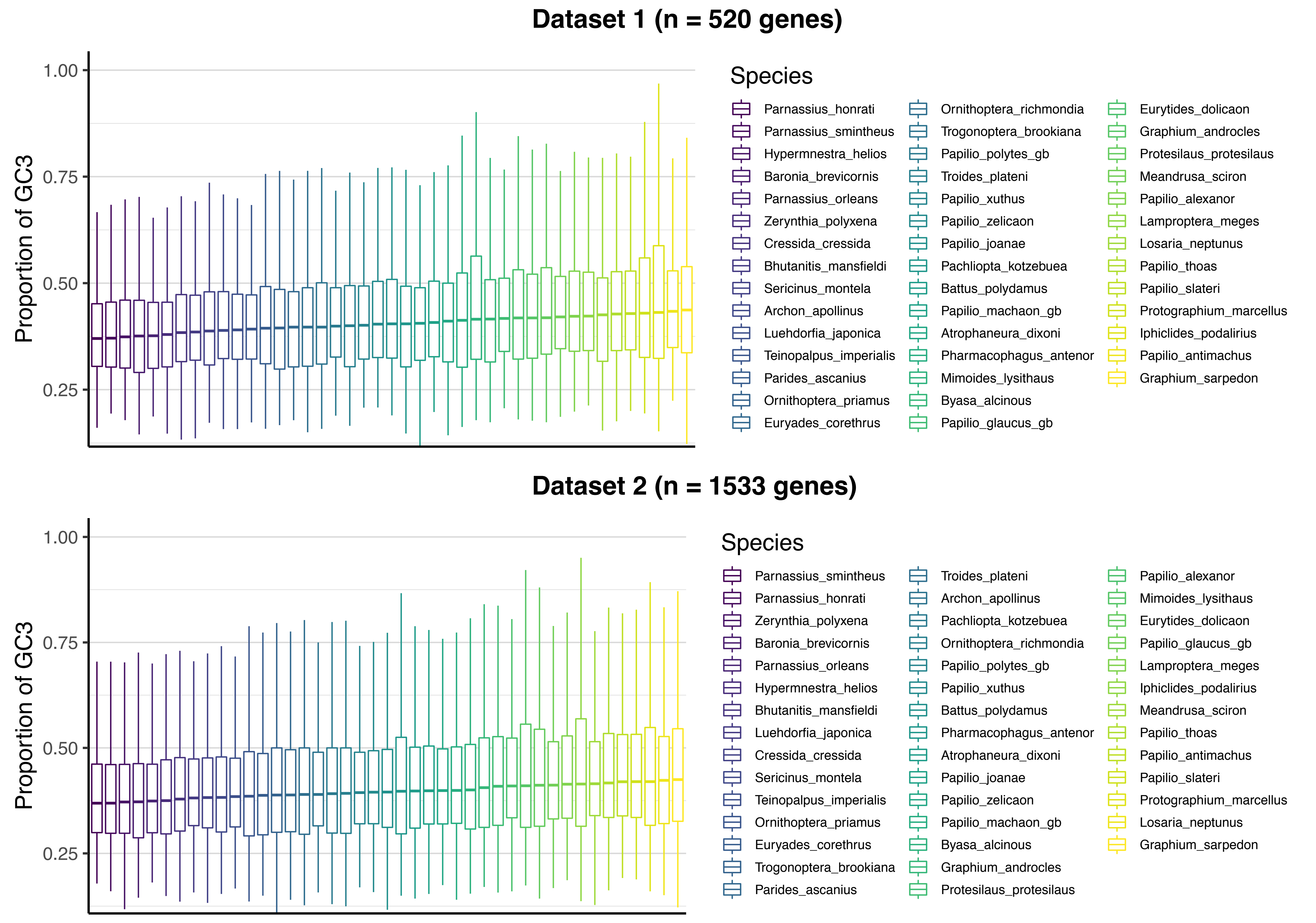
Dataset 1 (n = 520 genes)
Proportion of GC3
1.00
0.75
0.50
0.25
Species
Parnassius_honrati
Parnassius_smintheus
Hypermnestra_helios
Baronia_brevicornis
Parnassius_orleans
Zerynthia_polyxena
Cressida_cressida
Bhutanitis_mansfieldi
Sericinus_montela
Archon_apollinus
Luehdorfia_japonica
Teinopalpus_imperialis
Parides_ascanius
Ornithoptera_priamus
Euryades_corethrus
Ornithoptera_richmondia
Trogonoptera_brookiana
Papilio_polytes_gb
Troides_plateni
Papilio_xuthus
Papilio_zelicaon
Papilio_joanae
Pachliopta_kotzebuea
Battus_polydamus
Papilio_machaon_gb
Atrophaneura_dixoni
Pharmacophagus_antenor
Mimoides_lysithaus
Byasa_alcinous
Papilio_glaucus_gb
Eurytides_dolicaon
Graphium_androcles
Protesilaus_protesilaus
Meandrusa_sciron
Papilio_alexanor
Lamproptera_meges
Losaria_neptunus
Papilio_thoas
Papilio_slateri
Protographium_marcellus
Iphiclides_podalirius
Papilio_antimachus
Graphium_sarpedon
Dataset 2 (n = 1533 genes)
Proportion of GC3
1.00
0.75
0.50
0.25
Species
Parnassius_smintheus
Parnassius_honrati
Zerynthia_polyxena
Baronia_brevicornis
Parnassius_orleans
Hypermnestra_helios
Bhutanitis_mansfieldi
Luehdorfia_japonica
Cressida_cressida
Sericinus_montela
Teinopalpus_imperialis
Ornithoptera_priamus
Euryades_corethrus
Trogonoptera_brookiana
Parides_ascanius
Troides_plateni
Archon_apollinus
Pachliopta_kotzebuea
Ornithoptera_richmondia
Papilio_polytes_gb
Papilio_xuthus
Battus_polydamus
Pharmacophagus_antenor
Atrophaneura_dixoni
Papilio_joanae
Papilio_zelicaon
Papilio_machaon_gb
Byasa_alcinous
Graphium_androcles
Protesilaus_protesilaus
Papilio_alexanor
Mimoides_lysithaus
Eurytides_dolicaon
Papilio_glaucus_gb
Lamproptera_meges
Iphiclides_podalirius
Meandrusa_sciron
Papilio_thoas
Papilio_antimachus
Papilio_slateri
Protographium_marcellus
Losaria_neptunus
Graphium_sarpedon

Supplementary figure 25. The percentage of GC at third codon position (GC3) per branch for the branches with ("Yes", n = 14) and without ("No", n = 14) host-plant shift. For a given branch, the percentage of GC3 is the mean value of the species of a clade for which the branch is the root.

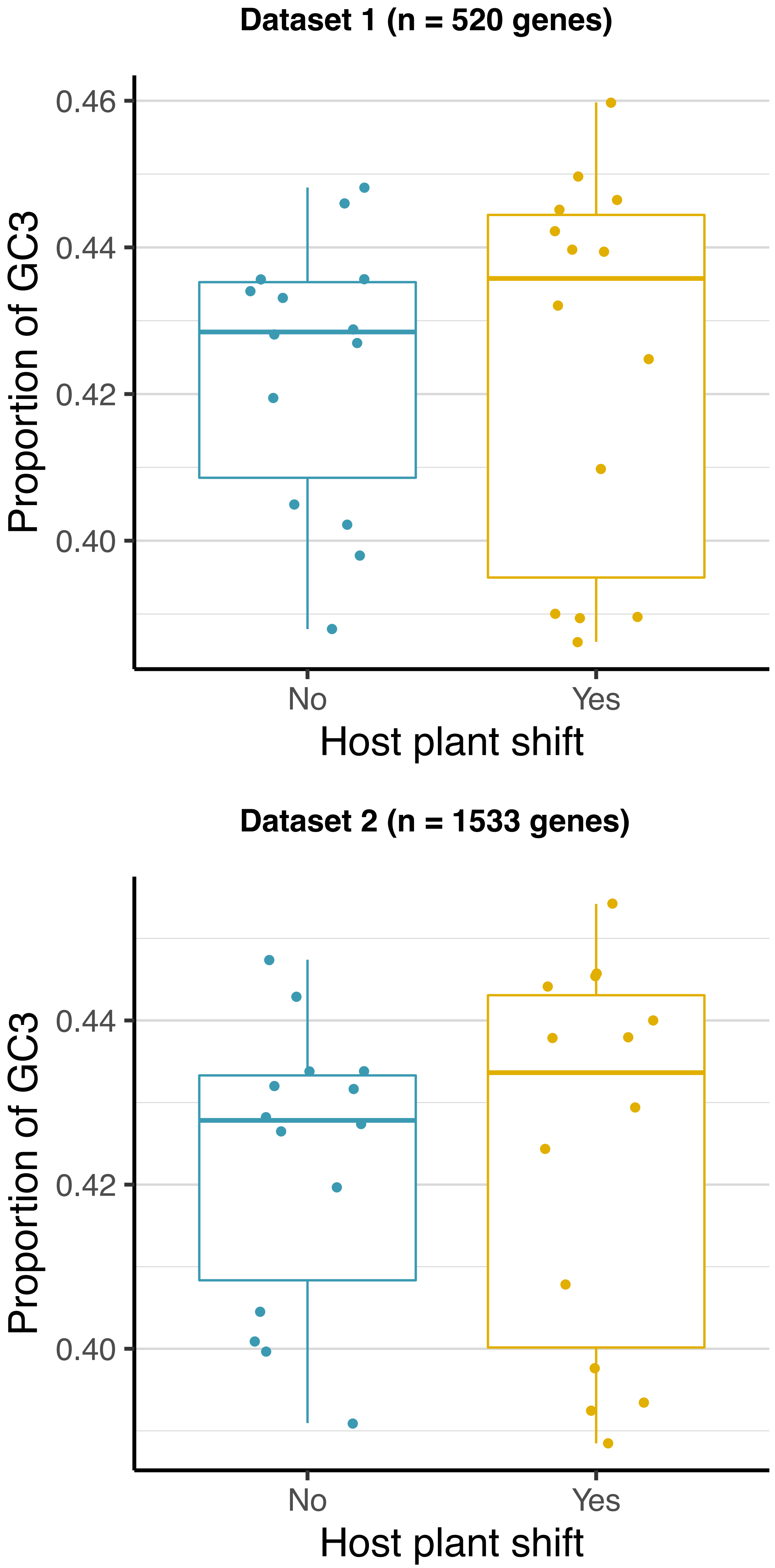

Dataset 1 (n = 520 genes)
0.46
0.44
0.42
0.40
Proportion of GC3
No
Yes
Host plant shift
Dataset 2 (n = 1533 genes)
0.44
0.42
0.40
Proportion of GC3
No
Yes
Host plant shift

Supplementary figure 26. Relationship between the percentage of GC at third codon position (GC3) and the number of positively selected genes per branch. For a given branch, the percentage of GC3 is the mean value of the species of a clade for which the branch is the root.

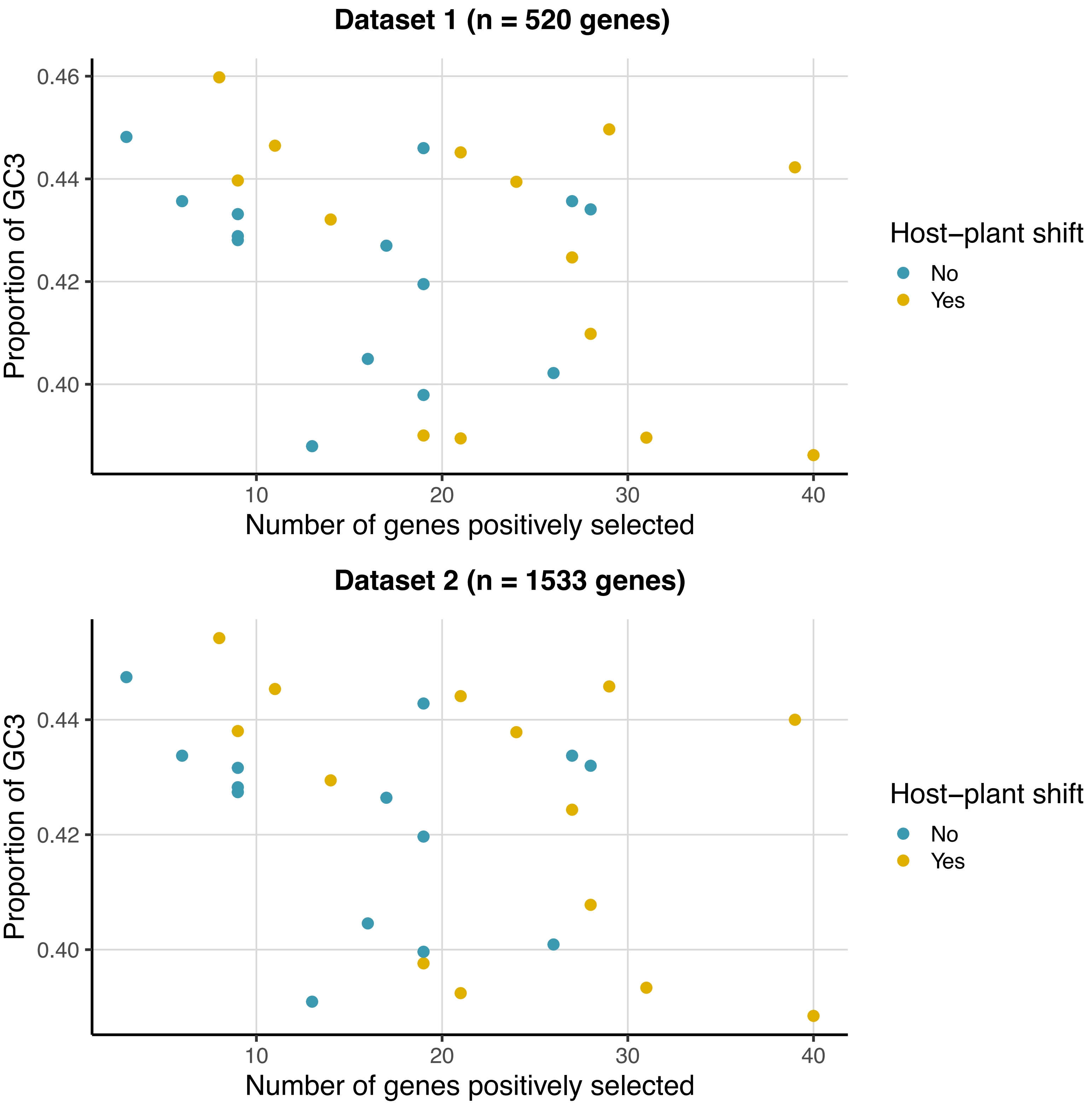

Dataset 1 (n = 520 genes)
Proportion of GC3
0.46
0.44
0.42
0.40
10
20
30
40
Number of genes positively selected
Host−plant shift
No
Yes
Dataset 2 (n = 1533 genes)
Proportion of GC3
0.44
0.42
0.40
10
20
30
40
Number of genes positively selected
Host−plant shift
No
Yes

Supplementary Table 1. Results from analyses of diversification rates performed with LASER. For clades shifting to new host plants, net diversification rates are estimated based on their crown age and extant species diversity using the method of moments. Net diversification rates for shifting clades are higher than the global rates of the family, suggesting that shifting to a new host plant confer higher rates of species diversification. Estimates of expected clade size based on the global diversification rates and crown age of shifting clades show that four clades diversified significantly faster than background diversification rates of non-shifting clades.

**Clades**	**Host plants**	**Crown age (Ma)**	**Extant species**	**Net diversification rate per Myr**			**Expected clade size**			***P* value**		
				**ε=0**	**ε=0.5**	**ε=0.9**	**ε=0**	**ε=0.5**	**ε=0.9**	**ε=0**	**ε=0.5**	**ε=0.9**
Leptocircini	Annonaceae	40.16	132	0.1043	0.0973	0.0646	84.9	112.9	196.7	0.0588	0.1254	0.2899
Papilio subg. *Papilio*	Apiaceae	13.54	9	0.1111	0.0966	0.0394	9.1	11.8	27.0	0.2166	0.3023	0.5693
Papilio subg. *Pterourus*, *Chilasa* and *Agehana*	Lauraceae	27.35	37	0.1067	0.0970	0.0539	29.0	39.4	82.8	0.1108	0.1980	0.4436
Papilio other subgenera	Rutaceae	35.61	163	0.1236	0.1157	0.0785	57.9	77.9	146.0	0.0023	0.0194	0.1262
Parnassius subg. *Parnassius*	Crassulaceae	10.77	19	0.2091	0.1866	0.0908	7.2	9.1	20.4	0.0012	0.0145	0.1812
Parnassius other subgenera	Papaveraceae	15.89	35	0.1944	0.1776	0.1016	11.1	14.7	33.6	0.0001	0.0020	0.0873
Papilionidae as a whole	-	67.28	570	0.0840	0.0798	0.0596	-	-	-	-	-	-

Supplementary Table 2. Summary of CodeML analyses for the Dataset 2 (1,533 genes). The ratio of genes under positive selection is reported for each branch. The columns "Plant shift", "Env. shift" and "Plant/Env. shift" report whether a shift and what type of shift occurred or not along each branch. The columns with "recovering the focal branch" indicate that the gene trees of this dataset recovered the focal branch as in the species tree.

Branch ID	Nb. of genes	Genes recovering the focal branch	Genes under positive selection	Genes under positive selection recovering the focal branch	% positive selection	% positive selection (recovering the focal branch)	Plant/Env. shift	Plant shift	Env. shift
Branch1	1533	1533	69	69	4.5010	4.5010	Host shift only	Host-plant shift	No shift
Branch5	1533	254	42	11	2.7397	4.3307	No shift	No shift	No shift
Branch6	1533	270	19	6	1.2394	2.2222	No shift	No shift	No shift
Branch7	1517	795	81	53	5.3395	6.6667	Host shift only	Host-plant shift	No shift
Branch8	1533	373	20	9	1.3046	2.4129	No shift	No shift	No shift
Branch9	1483	618	19	15	1.2812	2.4272	Host shift only	Host-plant shift	No shift
Branch10	1455	652	43	24	2.9553	3.6810	No shift	No shift	No shift
Branch11	1483	975	59	42	3.9784	4.3077	Host shift only	Host-plant shift	No shift
Branch12	1474	254	13	2	0.8820	0.7874	No shift	No shift	No shift
Branch13	1518	887	59	37	3.8867	4.1714	No shift	No shift	No shift
Branch14	1472	1026	42	25	2.8533	2.4366	Host and climate shifts	Host-plant shift	Climate shift
Branch15	1436	614	25	15	1.7409	2.4430	Host and climate shifts	Host-plant shift	Climate shift
Branch16	1530	1530	45	45	2.9412	2.9412	No shift	No shift	No shift
Branch17	1521	1521	56	56	3.6818	3.6818	Host and climate shifts	Host-plant shift	Climate shift
Branch18	1521	331	17	2	1.1177	0.6042	No shift	No shift	No shift
Branch19	1533	1533	104	104	6.7841	6.7841	Host shift only	Host-plant shift	No shift
Branch20	1533	617	65	38	4.2401	6.1588	No shift	No shift	No shift
Branch21	952	492	55	29	5.7773	5.8943	Host shift only	Host-plant shift	No shift
Branch22	1533	471	38	13	2.4788	2.7601	No shift	No shift	No shift
Branch23	1533	1533	80	80	5.2185	5.2185	Host shift only	Host-plant shift	No shift
Branch24	1533	314	22	11	1.4351	3.5032	No shift	No shift	No shift
Branch25	1325	958	61	43	4.6038	4.4885	Host and climate shifts	Host-plant shift	Climate shift
Branch26	1248	864	32	23	2.5641	2.6620	No shift	No shift	No shift
Branch28	1343	634	52	25	3.8719	3.9432	No shift	No shift	No shift
Branch29	952	952	30	30	3.1513	3.1513	Host shift only	Host-plant shift	No shift
Branch30	1483	1483	79	79	5.3270	5.3270	Host shift only	Host-plant shift	No shift
Branch31	1349	269	21	8	1.5567	2.9740	Host and climate shifts	Host-plant shift	Climate shift
Branch32	1330	321	32	10	2.4060	3.1153	No shift	No shift	No shift

Supplementary Table 3. Summary of diversification models in BAMM compared across a gradient of values for the Poisson process governing the number of rate shifts. The analyses show that a scenario with no shift of diversification best explained the macroevolution of Papilionidae. Although it might suggest that the Poisson prior has some effect on the analyses, it is important to note that all shifts are in common between the analyses. Based on the effective sample size (ESS) and the highest posterior distribution per number of shifts for the Poisson rate prior, we selected the model with a Poisson prior of 1. In addition this model did not favour a model with more shifts.

Poisson prior	ESS *logL*	ESS *Nb. shifts*	*Posterior distribution of the number of shifts*							
			0	1	2	3	4	5	6	7
1	403.5	501.0	0.60*	0.28	0.091	0.026	0.007	0.002	<0.001	<0.001
5	398.5	462.8	0.38*	0.31	0.17	0.083	0.031	0.014	0.005	<0.001
10	385.6	282.9	0.34*	0.3	0.19	0.093	0.045	0.019	0.006	0.004

Note: for each model, the asterisk (*) denotes the highest posterior probability for the number of shifts.

Supplementary Table 4. Comparison of the gene-tree inferences with the species tree to define the control and focal branches. The 'Branch recovered' column reports the number of gene trees (gene trees were inferred in Allio *et al.* 2020) showing the focal branch as monophyletic (i.e. including all genera for the focal clade), and the 'Branch not recovered' column indicates the number of gene trees for which the focal branch is not recovered. Overall, gene trees are more often in agreement with the species tree for the branches with a host-plant shift than without shift (P = 0.03, Wilcoxon-rank test).

Branch	Nb. genes	Branch not recovered	Branch recovered	Ratio	Plant/Env. shift	Plant shift	Env. shift
branch1	1528	665	863	0.77057	Host shift only	Host-plant shift	No shift
branch5	1528	1274	254	5.01575	No shift	No shift	No shift
branch6	1528	1258	270	4.65926	No shift	No shift	No shift
branch7	1528	725	803	0.90286	Host shift only	Host-plant shift	No shift
branch8	1528	1155	373	3.09651	No shift	No shift	No shift
branch9	1480	862	618	1.39482	Host shift only	Host-plant shift	No shift
branch10	1451	799	652	1.22546	No shift	No shift	No shift
branch11	1480	505	975	0.51795	Host shift only	Host-plant shift	No shift
branch12	1469	1215	254	4.78346	No shift	No shift	No shift
branch13	1513	626	887	0.70575	No shift	No shift	No shift
branch14	1467	441	1026	0.42982	Host and climate shifts	Host-plant shift	Climate shift
branch15	1431	817	614	1.33062	Host and climate shifts	Host-plant shift	Climate shift
branch18	1516	1185	331	3.58006	No shift	No shift	No shift
branch20	1528	911	617	1.47650	No shift	No shift	No shift
branch21	952	460	492	0.93496	Host shift only	Host-plant shift	No shift
branch22	1528	1057	471	2.24416	No shift	No shift	No shift
branch24	1528	1214	314	3.86624	No shift	No shift	No shift
branch25	1322	364	958	0.37996	Host and climate shifts	Host-plant shift	Climate shift
branch26	1319	383	936	0.40919	No shift	No shift	No shift
branch28	1341	707	634	1.11514	No shift	No shift	No shift
branch31	1347	1078	269	4.00743	Host and climate shifts	Host-plant shift	Climate shift
branch32	1328	1007	321	3.13707	No shift	No shift	No shift
