# Supplementary material for: Genome-wide macroevolutionary signatures of key innovations in butterflies colonizing new host plants
Source: Nat Commun. 2021 Jan 13;12:354. doi: 10.1038/s41467-020-20507-3 (PMC7806994; doi:10.1038/s41467-020-20507-3)
Supplement: Supplementary file 3 — Reporting Summary [file 41467_2020_20507_MOESM3_ESM.pdf]

Corresponding author(s): DBPR NCOMMS-20-25210-T

Last updated by author(s): Jun 30, 2020

# Reporting Summary

Nature Research wishes to improve the reproducibility of the work that we publish. This form provides structure for consistency and transparency in reporting. For further information on Nature Research policies, see our Editorial Policies and the Editorial Policy Checklist.

## Statistics

For all statistical analyses, confirm that the following items are present in the figure legend, table legend, main text, or Methods section.

| n/a | Confirmed | |
|---|---|---|
| ☐ | ☒ | The exact sample size (*n*) for each experimental group/condition, given as a discrete number and unit of measurement |
| ☐ | ☒ | A statement on whether measurements were taken from distinct samples or whether the same sample was measured repeatedly |
| ☐ | ☒ | The statistical test(s) used AND whether they are one- or two-sided<br>*Only common tests should be described solely by name; describe more complex techniques in the Methods section.* |
| ☒ | ☐ | A description of all covariates tested |
| ☐ | ☒ | A description of any assumptions or corrections, such as tests of normality and adjustment for multiple comparisons |
| ☐ | ☒ | A full description of the statistical parameters including central tendency (e.g. means) or other basic estimates (e.g. regression coefficient) AND variation (e.g. standard deviation) or associated estimates of uncertainty (e.g. confidence intervals) |
| ☐ | ☒ | For null hypothesis testing, the test statistic (e.g. *F*, *t*, *r*) with confidence intervals, effect sizes, degrees of freedom and *P* value noted<br>*Give P values as exact values whenever suitable.* |
| ☐ | ☒ | For Bayesian analysis, information on the choice of priors and Markov chain Monte Carlo settings |
| ☒ | ☐ | For hierarchical and complex designs, identification of the appropriate level for tests and full reporting of outcomes |
| ☒ | ☐ | Estimates of effect sizes (e.g. Cohen's *d*, Pearson's *r*), indicating how they were calculated |

*Our web collection on statistics for biologists contains articles on many of the points above.*

## Software and code

Policy information about availability of computer code

| | |
|---|---|
| Data collection | No software was used for the data collection. |
| Data analysis | 1 - Species-level phylogenies of Papilionidae and of Aristolochiaceae with Sanger DNA data:<br>(a) Supermatrix construction: MAFFT 7.110 and Mesquite 3.1<br><br>(b) Phylogenetic inferences: PartitionFinder 2.1.1, IQ-TREE 1.6.8 (With ModelFinder), MrBayes 3.2.6 (with BEAGLE), and Tracer 1.7.1<br><br>(c) Molecular dating: BEAST 1.8.4, Tracer 1.7.1, and LogCombiner 1.8.4<br><br>(d) Estimation of ancestral host-plant preferences (for Papilionidae only): Dispersal-Extinction-Cladogenesis (DEC), BayesTraits 3.0.1, and Mesquite 3.1 (Markov 1-parameter model)<br><br>(e) Historical biogeography: DEC<br><br>(f) Birth-death analyses (for Papilionidae only): R-package LASER 2.3 (bd.ms, crown.limits and crown.p functions), R-package diversitree 0.9-10 (MuSSE model), R-package RPANDA 1.3 (fit_bd function), BAMM 2.5 with the R-package BAMMtools 2.1, RevBayes 1.0.10, and R-package TESS 2.1 (CoMET model)<br><br>2 - Genome-wide analyses for Papilionidae only:<br>(a) Dataset construction: Trimmomatic 0.33, SOAPdenovo-63mer 2.04, BLAST, TranslatorX, Croco 0.1, OrthoFinder 2.2.0, HMMcleaner 1.8, trimAl 1.2rev59, and SeaView 4 |

(b) dN/dS analyses: PAML 4 (CodeML, branch-site model)

(c) Sensitiviity analyses: Home-made C++ program created with BIO++ library, R-package tidyverse, R-package cowplot, and SeaView 4

(d) Gene ontology: PANTHER 14 and EggNOG 5.0 (eggNOG-mapper v2 with the LepNOG database)

For manuscripts utilizing custom algorithms or software that are central to the research but not yet described in published literature, software must be made available to editors and reviewers. We strongly encourage code deposition in a community repository (e.g. GitHub). See the Nature Research guidelines for submitting code & software for further information.

## Data

Policy information about availability of data

All manuscripts must include a data availability statement. This statement should provide the following information, where applicable:

- Accession codes, unique identifiers, or web links for publicly available datasets
- A list of figures that have associated raw data
- A description of any restrictions on data availability

All data, including supermatrix datasets (for phylogenetic analyses), phylogenetic trees, host-plant preferences, species geographic distributions, gene alignments (for dN/dS analyses) and bioinformatic scripts, that are necessary for repeating the analyses described here have been made available through the Figshare digital data repository (https://figshare.com/s/1ce98308a3c012514857).

# Field-specific reporting

Please select the one below that is the best fit for your research. If you are not sure, read the appropriate sections before making your selection.

☐ Life sciences ☐ Behavioural & social sciences ☒ Ecological, evolutionary & environmental sciences

For a reference copy of the document with all sections, see nature.com/documents/nr-reporting-summary-flat.pdf

# Ecological, evolutionary & environmental sciences study design

All studies must disclose on these points even when the disclosure is negative.

| | |
|---|---|
| Study description | This study aims at testing whether host-plant shifts have impacted both genomic adaptation and species diversification in a butterfly family (Papilionidae) over geological times. |
| Research sample | The insect family Papilionidae (the swallowtail butterflies) and the plant family Aristolochiaceae (the birthworts).<br>The research sample included all species for which we could obtain DNA sequence data such that they could be included in comprehensive phylogenetic trees. The sample was global in scope, and there were no data exclusions. |
| Sampling strategy | Sample size was determined by the availability of genetic and geographic data for both focal clades. We used all available data in our study. The sample size used in our study exceeds the vast majority of published genome-wide studies at a macroevolutionary scale. |
| Data collection | DNA sequence data were extracted from public DNA sequence databases (GenBank or Ensembl, most of which have been previsously generated by the authors). Species geographic distribution data were extracted from the literature and our previous works. Ecological data for the swallowtails (i.e. host-plant preferences) were also extracted from the literature and our previous works. |
| Timing and spatial scale | Global scale<br>Throughout the Cenozoic |
| Data exclusions | No data were excluded from the analyses. |
| Reproducibility | Our data are not experimental and experiments were thus not replicated. However, we used multiple distinct statistical tests (e.g. BAMM, MuSSE, RevBayes, RPANDA) and all approaches yielded concordant results. We also tested extensively for artifacts that might explain the genomic pattern. |
| Randomization | We did not perform an experiment and there was thus no group allocation. We used all species of swallowtail butterflies and birthwort plants for which (1) DNA sequence data were available, and (2) spatial and ecological data were available. There was no further group partitioning of data beyond the natural groupings associated with geography and clade membership. |
| Blinding | Blinding was not relevant to our study, because all available data were used (our study did not perform an experiment). |

Did the study involve field work? ☐ Yes ☒ No

# Reporting for specific materials, systems and methods

We require information from authors about some types of materials, experimental systems and methods used in many studies. Here, indicate whether each material, system or method listed is relevant to your study. If you are not sure if a list item applies to your research, read the appropriate section before selecting a response.

## Materials & experimental systems

| n/a | Involved in the study |
| --- | --- |
| ☒ | ☐ Antibodies |
| ☒ | ☐ Eukaryotic cell lines |
| ☒ | ☐ Palaeontology and archaeology |
| ☒ | ☐ Animals and other organisms |
| ☒ | ☐ Human research participants |
| ☒ | ☐ Clinical data |
| ☒ | ☐ Dual use research of concern |

## Methods

| n/a | Involved in the study |
| --- | --- |
| ☒ | ☐ ChIP-seq |
| ☒ | ☐ Flow cytometry |
| ☒ | ☐ MRI-based neuroimaging |
