# Supplementary material for: Genome-wide macroevolutionary signatures of key innovations in butterflies colonizing new host plants
Source: Nat Commun. 2021 Jan 13;12:354. doi: 10.1038/s41467-020-20507-3 (PMC7806994; doi:10.1038/s41467-020-20507-3)
Supplement: Supplementary file 4 — Description of Additional Supplementary Files [file 41467_2020_20507_MOESM4_ESM.pdf]

Description of Additional Supplementary Files

Supplementary Data 1. Genomic coverage for each gene and for each species. An average coverage of 26.7x is found for all genes and all species with a minimum average coverage of 4.74x for Hypermnestra helios and a maximum average coverage of 110.08x for Papilio zelicaon.

Supplementary Data 2. Information on orthogroups of Dataset 2 (1,533 genes). The columns 2 to 6 indicate whether the genes are under positive selection and along which branch (column 'Branch ID' see Supplementary Fig. 18 for the annotated tree with branch numbers). The column 'Papilio xuthus seq ID' is the GenBank accession number for the corresponding sequences in Papilio xuthus. The column 'PANTHER family:subfamily accession' is family and subfamily accessions, and the column 'PANTHER family name' list the names for gene families based on PANTHER classification (see http://pantherdb.org/ for more information). Finally, 'HMM e-value score' is the Hidden Markov model e-value score, as reported by HMMER (Eddy 2011) performed through the online PANTHER scoring tool ftp://ftp.pantherdb.org/hmm_scoring/current_release. Following PANTHER recommendation, we have not considered e-values above 10-11 as significant. The EggNOG results are also reported with the columns 'EggNOG family name' and 'EggNOG function' providing information about families associated with each orthologous gene. The 'EggNOG seed ortholog evalue' column indicates the e-value reported by EggNOG for each orthologous gene.
