# Supplementary material for: Genome-wide macroevolutionary signatures of key innovations in butterflies colonizing new host plants
Source: Nat Commun. 2021 Jan 13;12:354. doi: 10.1038/s41467-020-20507-3 (PMC7806994; doi:10.1038/s41467-020-20507-3)
Supplement: Supplementary file 2 — Peer Review File [file 41467_2020_20507_MOESM2_ESM.pdf]

Reviewer #1 (Remarks to the Author):

This manuscript provides an in depth look at co-evolution between the Papilionidae family of butterflies and the plants on which they prey. This study is a rigorous effort which is comprised of several large datasets used to integrate signals of co-evolution to form a larger picture about the history of these groups. The authors study diversification rates, speciation times, and molecular evolution to convincingly gather evidence for this arms race. I have only two major comments and a few minor comments for the authors to consider.

Major points

1. For the positive selection analysis, it is unclear whether the authors used a single species tree topology for all genes, or whether they used the individual gene tree inferred from that gene for each test. Because gene trees can be discordant from inferred species relationships, it is critical that gene trees are used for these tests. Using a single species tree can spuriously inflate substitution rates on some branches.

2. PAML's site-based tests have recently been shown to be sensitive to multi-nucleotide mutations (MNMs; https://www.nature.com/articles/s41559-018-0584-5). Do the authors find that the sites in genes under positive selection through the branch-site test in their data contain MNMs? If so, the authors should consider mentioning this in the manuscript. I do not think this will impact the results because the authors have used proper controls (non-shifting sister lineages), and it will be likely that all branches tested would suffer equally from MNMs. I only say this to make the authors aware of it, not to specifically request it be used (because I haven't had a chance to test its efficacy yet either), but HyPhy recently released a method that compares models with and without MNMs: https://github.com/veg/hyphy-analyses/tree/master/FitMultiModel.

Minor points

1. Figure 2: I think the y-axis could be labeled more descriptively for those unfamiliar with Bayesian sampling methods.

2. Line 564: Was the Aristolochiaceae phylogeny also inferred first with IQ-tree? I don't see that mentioned here.

3. Line 801: It is clear to me from the main text and Figure 4 that you are comparing lineages with a host-plant shift to those without, but on this line it is ambiguous. Perhaps consider the following wording:

"We tested if branches representing inferred host-plant shifts along the phylogeny of swallowtails have more genes with dN/dS > 1 than lineages that did not have an inferred shift."

4. Line 862: "Overall, given the our alignment" should read "Overall, given our alignment".

Reviewer #2 (Remarks to the Author):

Title:
Genome-wide macroevolutionary signatures of key innovations in butterflies colonizing new host plants

Summary

The authors have brought together diverse datasets and analyses to study the evolutionary consequences of plant-insect interactions in a classic group of species. What is novel and important here is the extension of plant-insect coevolutionary studies to the genomic scale, to begin using an unbiased approach to searching for evolutionary consequences of these interactions. In order to provide the proper context for this type of analysis, the authors generate time calibrated phylogenies for the plant and insect clades, followed by assessing the spatial and temporal context of these clades during their early evolution, finding that these plants and insects arose close in time and geographic space. They further mapped the hostplants that the butterflies use upon the butterfly phylogeny, reconstructed their ancestral states and were thus able to place major hostplant shifts in a temporal context, against which they assessed butterfly species diversification rates in light of hostplant shifts. This revealed some dramatic increases in speciation associated with such shifts, as expected by coevolutionary theory. Then, using a previously published large dataset of low coverage genomes, they assessed whether host shifts were associated with an increase in signatures of macroevolutionary positive selection using ~1500 genes, finding significantly more genes under positive selection in branches associated with shifts to novel hostplants (as compared to control branches).

At face value, this is an important and significant step forward in the study of plant insect interactions, testing the expectations of coevolutionary interactions. However, there are a number of issues that need to be resolved before I think this is ready for a journal such as Nature Communications. If the authors are able to address these issues sufficiently, I think this could make a significant contribution to the literature. However, some of these issues are very serious and at the core of the paper.

Major concerns.

Molecular tests of selection

The biggest question I have is with regards to what the authors have actually detected in their study of positive selection. First, given their model specifications, as I understand them, they have branches in their tree that are either involved with shifting to a novel hostplant vs. those that are not and are used as a control. They then estimate, for about 1500 genes, site branch values of omega and then determine those that are significant. Then they group these significant genes by what type of branch they occurred upon (a shifting branch vs control branch), and they find more sig. genes on shift branches compared to controls. This leaves me with several questions.

Is the total number of genes reported under positive selection the number of genes with even just one site in one branch of their analysis? Did they look at the BEB values per site? Did all such genes have significant BEB values, or are they only using the lnL values at the gene level? What about genes with more than one site or more than one branch significant? Were these genes evenly distributed among branches or were some branches having more such genes? Complicating this, there is a lack of clarity and descriptions about what was done for this analysis. There is no summary table per gene for the lnL or BEB values, and I can’t even find the raw totals of these analyses (genes per group, etc).

Second, their analysis did not look across the entire genome. Rather it focused upon only a very small set of genes ~1500 and ~500, due to their low quality data and post filtering. But, using even this small set they identified many genes under positive selection at every node in their phylogeny. If I am interpreting their findings correctly, they infer that out of random set of 1500 genes, they are detecting between 10 to 20 genes under positive selection at nearly EVERY branch across the entire phylogeny. This is true for even the 520 gene dataset (SF22), where there are about 26 branches, where on average 15 genes per branch were found significant, this is therefore 26*15 = ~390 genes identified as positively selected, which is > 50% of genes. Perhaps I have missed something, but if this is what the authors are claiming, well, that is simply not possible based upon the existing literature in comparative genomics when looking at random gene sets. Rather, this is suggestive of a

high false positive rate arising from their use of low quality genomic datasets.
Along these lines, I want to applaud the authors efforts to assess these issues by conducting several posthoc analyses of their genomic data used in the molecular tests analyses. First, they do find evidence that missing data and GC3 content increase false positive rate in detecting positive selection (L831-833). Specifically, there is more missing data and higher GC3 levels in genes detected as being under positive selection, which is a hallmark of false positives. Great that they detected this, and they rightly point out that this could be a direct effect of lower quality data and GC biases. They then proceed to assess whether missing data and GC3 levels are significantly different between their compared groups, and find that they are not. This leads the authors to conclude that while the false positive rate is high, it is equally distributed among groups (e.g. host-shift vs. control), thus the differences between the two groups are meaningful – i.e. the higher number of genes under positive selection in the host shift group is biologically meaninful. But, I have a different take away from these posthoc forensic analyses than the authors. In contrast, I conclude that the error rate is so high as to be problematic and to call into question the use of the low coverage dataset in general for this type of analysis.
Going a bit deeper, when I look at your posthoc analyses of the codeML results, it looks like nearly your entire dataset has on average 10-15% missing data. I wonder what that means for your analyses. Doesn't it suggest that the genomic coverage is rather poor for many species? Gene regions should be the best assembled portion of the genome, thus this is high % of missing data is troubling. Moreover, what was the data quality in these regions that were used for analysis. Since they lack compete coverage, this suggests that the contigs themselves didn't have much coverage, and thus the assembly from which these exons were mined likely, on average, do not have had much sequence coverage and therefore have a high sequence error rate, as contig accuracy in genome assembly comes from high genomic coverage. Any such error rate inflation in these regions will be detected as positive selection given the increased likelihood of AA changes via such errors.
Unless the authors can somehow improve their dataset to reduce these false positives, I find interpretation of their results very difficult at best. A suggestion would be if they were not just mining exons from draft genome assemblies, but also assessing the depth of sequencing coverage for each such exonic region, since if those regions have low coverage, they likely have high error and should not be used.

Also, the authors say a lot about their functional annotations, yet I find these have not be used to any significant extent in their analyses nor does this add much insight at all. Perhaps the authors could use their annotations in a more insightful way or push this more to the back of the MS.

Comparison of findings to previous work
The authors generated two time calibrated phylogenies, one for their butterflies and one for the major plant clade. How do their results compare to previous analyses, as this is never discussed by the authors, making it difficult to evaluate the accuracy and importance of this contribution.

Also, and perhaps one of the most difficult issues the authors need to address are the findings of a recent study that documents the inherent danger in conducting the type of evolutionary analyses that are at the core of their paper (see Louca, S., and Pennell, M.W. (2020). Extant timetrees are consistent with a myriad of diversification histories. Nature 580, 502–505; and Pagel, M. (2020). Evolutionary trees can't reveal speciation and extinction rates. Nature 580, 461–462.). Specifically, estimating time dependent changes in speciation rate is potentially fraught with serious error and over interpretation. However, nowhere in the current MS do the authors show any circumspection regarding the potential errors in their analysis. I find that a troubling.

L176-178: This is a strange argument. Given my understanding of the literature and plant insect interactions community, I don't think that anyone believes that single genes or a single gene family are enough to act as a key innovation. Rather, everyone believes that key innovations are complex phenotypes, ranging from hostplant preference, to detoxification, coupled with all the changes

necessary for exploiting this resource due to the ecological dynamics involved. Researchers have focused upon specific genes cause that is all they could do to make the connection between genotype and phenotype, and no one to my knowledge is believing that these genes or gene familes was the whole story. Thus, this statement is really a false straw man that reflects misunderstanding upon the authors than the refences in question, or the community as a whole. I suggest rephrasing a bit to reflect the complexity of the issue. Certainly highlight your novel perspective and contribution here, but please don't use the existing straw man argument, as it doesn't reflect the literature in a way you would want to be remembered for.

Clarity of methods and datasets is lacking:
Beyond some of the issues I mentioned above, the reader needs to see summary statistics for dataset completeness for phylogenetic analysis of Papilio, as well as plant clades.
My preliminary look at their input datasets suggests that there is a large number of missing genetic content for many of their species. How does this affect their results? Usual analyses looks at these issues and quantify this effect.

Overstatement:
Language throughout the manuscript over interprets their findings. This occurs in several places. First, the authors argue that the butterfly and plant clades are "synchronous", which is difficult to assess and given their results do not appear to be synchronous to this reader. Rather they should be stating that they appear to have crown ages very close in time. Specifically, the peak of their posterior distributions are not identical (Fig1), nor should even such results be fully trusted as time calibrations are much like reading the entrails of chickens – so best to be circumspect here. What can be concluded is that temporal estimates for the two clades appear to be very close in evolutionary time.

Other example of overstatement are in regards to the phylogenetic tree, to the dates, to the reconstructed hostplants, historical ranges, to genes identified as being under positive selection – ALL of these are mere hypotheses. But, the authors use phrasing that states these findings as if they are somehow "the truth", e.g. L145 "showed more genome-wide molecular adaptations", L88 "results show that both Papilionidae and Aristolochia were ancestrally co-distributed", etc.

L195: "our study reveals" is an overstatement that needs to be properly framed, along the lines of understanding that you have not shown anything, merely obtained sig Pvalues, which could be driven by many factors (e.g. model misspecification, sequence errors, all the issues that confound test of positive selection, etc). Without such perspective, your paper is grossly overselling its achievements.

Diversificaiton
I find it great that the authors used 5 different methods to get at their diversification dynamics. Wonderful. However, I can't find a proper discussion comparing these results and how you decided to report what you did. Specifically, the results from RPANDA and BAMM for clade specific diversification rates through time appear to be quite different, with the BAMM results not agreeing with those from RPANDA. Rather than talking about this difference, the authors only use the RPANDA model results. I greatly appreciate the authors using both methods and reporting their findings, but this raises some complications with interpretation of the results. Also, it is not clear what results you are reporting in the main text compared to all the different analyses you ran.
In a similar vein, Fig. 3 does a good job of showing that the average rates per clade do not show a strong increase in diversification associated with hostplant shifts. These differences need to be tested statistically (L102-103) not just qualitatively reported, and it looks like most clades will not be different from the Aristolochiaceae-feeding swallowtails. But, your time dependent estimates of diversification show that these averages can be misleading (Fig3b). You should be discussing this difference between these two approaches and how the latter is more informative. Currently you

present both as showing the same thing, which they do not.
Further, why are the authors not showing their results for Fabaceae, Zygophyllaceae, Magnoliaceae? Is this because they have lower diversification rates after their shift, and therefore don't fit their expectations, or cause these clades are small? In sum, there appear to be as nearly as many clades increasing as decreasing in diversification post host shifts, if these are included … Some explanation needs to be made as to why these clades are excluded from the main text and the general impression the authors are making in the main text, as what they are currently arguing doesn't appear supported by their own findings.
L105-107: "no observed classical slowdown of diversification". Give the authors own data, I disagree with this statement. Specifically, the results and the authors statements "A model with a slowdown of diversification through time explained the diversification of Annonaceae feeders, Lauraceae feeders, and Papaveraceae feeders (SF13). Clearly Lauracea and Papaveraceae, which are two with the largest increase, show a dramatic decrease in diversification rate through time. If you are looking at Papilio as a whole, perhaps this is an issue of temporal / species diversity scale.

Molecular tests of positive selection
CodeML specifications: I find your code a bit strange in a way that could inflate your estimates of omega. Why is your initial omega in your alternative analyses starting at 1.5, which would be strong positive selection as a prior? Won't this greatly bias your analyses? In the original control file, this is 0.4 and I've never seen this changed in an analysis. Why are you doing this? What happens if you use the default 0.4 for your alternative model?

Data availability:
This is insufficient details and datasets in the current MS, as well as upon the Figshare data repository, leaving the reader unable to assess the quality of the work performed.

Thus, this statement by the authors is incorrect at the time of my accessing the repository, "All data, including supermatrix datasets (for phylogenetic analyses), phylogenetic trees, host-plant preferences, species geographic distributions, gene alignments (for dN/dS analyses) and bioinformatic scripts, that are necessary for repeating the analyses described here have been made available through the Figshare digital data repository"

There are no gene alignments at this repository and many of the bioinformatic scripts are missing. For example, where are the R scripts used for analyses? (e.g. for the Lazer, MuSSE, RPANDAS, BAMM analyses?). Also, where is the table of codeML results per gene per site, lnL scores, etc? Further, while I am greatly excited by your use of genomic data from 45 species, the reader needs to know much more about the average statistics for these different genomes, in terms of coverage, assembly quality, gene coverage, etc., as you have gathered them all here for your paper, you should report upon their state in a unified fashion.

Minor Comments
L41: citation for this?

L49: evolutionary "linkages" …. This phrasing is unclear about the meaning, please restate.

L68: which family, plant or insect? More clarity please.

L77: "highly synchronous" is perhaps an overstatement, rather place these estimates into a more quantified statement for the reader to appreciate these findings.

L102: upshifts when talking in the same sentence as shifts in diversification …. That's a lot of shifts. Isn't "increase" good enough for one of these?

L146: not clear to me why there are two pvalues and why they are separated by a "/" symbol … this is a bit confusing to me.

157-160: great that you checked these issues. You should also be including these aligned gene datasets for review as well as publication, as these need to be assessed.

L165-166: you might want to restate this for clarity.

Other issues that might have been covered above.

What were the exact commands used in iQtree?

My Bayes run details. What where the minimum thresholds used for the ESS and PSRF values for each parameter?

What were the ESS minimum values from the BEAST temporal calibration analysis?

Where are the R scripts used for analyses? (e.g. for the Lazer, MuSSE, RPANDAS analyses?)

BAMM analyses can be very sensitive to priors, great that you used 3 different CPP values. How did their convergence look, as I can't find any discussion of that.

L169-171: you are highlighting the number of gene with unknown function by identifying total number with function. This makes for a strange sentence. Rather, say that 30% lacked annotation, … But, I'm surprised that you had functional information for 70% of your genes. What thresholds on your annotations did you apply. For example, the authors for eggnog recommend using the cutoff of 200 on their scoring metric, rather then just using the annotations from all the genes that are returned. I imagine there should be some similar filter for PANZER. What this applied? Does it change these numbers?
Also, when looking at your annotation table, even though you "annotation" from PANZER, there is no gene description. Is this cause there is only GO information? How general is that GO information, as I usually find it rather useless. Thus, please step back and have a second look at your annotations. I don't think they are as informative as you think. Also, do the gene annotations between PANZER and eggNOG agree? If you only looked at genes with descriptions, or informative GO assignemtn, how many genes actually have useful annotaitons. I'd be surprised if > 40% did … thus I'm surprised you make a big deal of this, as I can't find anything to support these claims in your findings and you don't even use much of your annotation information at all in your results …

In your Table S2, you should also include your eggNOG score for annotation, just as you did the HMM e-value score for the PANZER annotation.

Fig 4 is showing gene totals from which dataset?

L180: "deeper adaptation" …. Can you please restate for greater clarity.

L182-191: I suggest that here is a good place to say that your analyses have generated a list of novel candidates possibly involved in plant-insect interactions, which require functional validation, as what you have generated are a series of hypotheses.
L196: "more pervasive than previously recognized". I disagree with this statement. Rather, you study is the first to be able to look at this scale, and it simply is able to get at this question in a more comprehensive / global fashion. Everyone in the plant-insect community knows that there are certain to be many genes involved, as host plant shifts are complex.
L198: "more adaptations than in just the gene families in detoxification pathway". This is falling into the same logical fallacy as earlier sentiments. Please revise. Again, suggest that you argue that your

able to extend beyond the candidate gene/gene family perspective that others have previously used (cause that was all they had for study and focus – they didn't have the glorious dataset you have).
L200-202: Certainly, this is a true statement and I think the community expects this. You're the first to be able to gain such insights. Thus rather than saying you've discovered what is likely to be more general, I'd restructure your argument throughout the paper to be that this is expected, and here we provide evidence of it at the genomic scale.
Figure 2: I suggest alternating the gray/clear color pattern for the epochs, such that your images of the hostplant and butterfly are on a white background rather than gray.
Fig. 3. I suggest having straight arrows or now arrows in panel B to show the shift, as this gives a strange impression of when the shift occurred. Horizontal lines would be better.
SM15: please include the species names on this tree so that the reader can explicitly see which branches on the tree are what in their analysis.
L773: "for all consisting on all genera" is unclear, please restate this sentence for greater clarity.
L771: a robust phylogenomic tree of Papilionidae. This is never demonstrated, but rather claimed. I find now tree for these 45 taxa that has bransh support, and even then, what is needed, given the dataset of > 6000 genes to build this, are reports of gene concordance factors (implemented easily in iQtree) to assess how well supported this tree actually is. Currently the reader is directed to SF18, but this lacks taxa labels and branch support information. Given the low gCF and sCF in the Allio et al. 2020 paper, I assume these are similar here, and thus, calling this phylogeny robust is inappropriate as there remains substantial potential discordance in the nodes.
How did the BAMM model specifications of priors influence results? This was never properly discussed, as it usually has a very large effect.
What is the false discovery rate used for the dN/dS analysis of the codeML results?
What value you were using for the pvalue for the per gene omega?

Reviewer #3 (Remarks to the Author):

The authors present a highly interesting study on the drivers of diversification in the butterfly family Papilionidae. It is one of the first to link changes in host plant use and range shifts to new biomes to changes in genomic signatures of selection. The data used to investigate these connections is comprehensive and adequate for the questions asked. As I am not an expert in functional genomics, I will restrict my comments to the phylogenetics, timing of divergence and diversification analyses. These have been done rigourously, as one has come to expect from the Condamine lab. The authors use the latest methods, and justify their use of the currently contentious analyses of diversification dynamics based on molecular phylogenies. With the addition of the historical biogeography analysis (also well done), these analyses form the bases for what is the main news of the study, the genomic signatures of selection associated with changes in host plant use. I leave it to reviewers with functional genomic expertise to tell us whether those analyses are done according to standards and whether interpretations of results are correct, but the bases for those analyses are solid.

Signed,
Niklas Wahlberg

REVIEWER COMMENTS

Reviewer #1 (Remarks to the Author):

This manuscript provides an in depth look at co-evolution between the Papilionidae family of butterflies and the plants on which they prey. This study is a rigorous effort which is comprised of several large datasets used to integrate signals of co-evolution to form a larger picture about the history of these groups. The authors study diversification rates, speciation times, and molecular evolution to convincingly gather evidence for this arms race. I have only two major comments and a few minor comments for the authors to consider.

Thank you for reviewing our study and providing constructive comments.

Major points

1. For the positive selection analysis, it is unclear whether the authors used a single species tree topology for all genes, or whether they used the individual gene tree inferred from that gene for each test. Because gene trees can be discordant from inferred species relationships, it is critical that gene trees are used for these tests. Using a single species tree can spuriously inflate substitution rates on some branches.

Thank you for pointing this out. As shown in Dieckmann & Pereira-Leal (2015 - Evol. Bioinfo.), the choice of a tree is an important factor for the branch-site analysis of positive selection. Indeed, constraining the topology for a given gene may lead to overestimating the number of substitution events for the constrained branches (as shown by Mendes & Hahn 2016 - Syst. Biol.) and so could lead to overestimating the dN/dS ratio. Estimating dN/dS over thousands of gene trees would make the branch comparison unequal between control and test branches. Indeed, in a given gene tree it is likely and expected that the species topology may not be recovered, which results in a different number of branches compared to the species tree. For instance the host-plant shift to Annonaceae might disappear in certain proportions of genes. We thus decided to estimate dN/dS on a fixed species tree topology for all genes to be sure to be able to measure this ratio for each gene. Because we focus on particular branches (i.e. host-plant shift branches) that must be present in the topology, we fixed the tree topology for all genes.

However, given that this issue can lead to a bias in our analysis, we computed the number of gene trees that did not recover the branches of interest. We then checked whether the branches leading to a host-plant shift were more often unrecovered than the branches without shift (i.e. the so-called “control branches”). We found that overall the control branches were less often recovered than host-plant shift branches (P = 0.030, Wilcoxon-rank test; data presented in Supplementary Table 6).

If gene tree/species discordance leads to overestimation of positive selection, this overestimation should be higher for control branches than for host-plant shift branches. To confirm that the results hold despite this bias, we filtered out the gene trees for which the focal branches are recovered (in agreement with the species tree) and used these genes to re-estimate the proportion of genes under positive selection among this new set of genes. One can expect to lose the significance given that we decrease a lot the number of genes for each branch, but we surprisingly found that the P-value remains significant (P = 0.0444, Wilcoxon-rank test; more genes during host-plant shifts than along control branches). These results are now reported in Supplementary Table 3.

As a consequence, we believe that the main message of the study holds, i.e. more genes under positive selection along branches shifting to new plants. Finally, we have added explanatory sentences in the revised version of the manuscript.

2. PAML's site-based tests have recently been shown to be sensitive to multi-nucleotide mutations (MNMs; https://www.nature.com/articles/s41559-018-0584-5). Do the authors find that the sites in genes under positive selection through the branch-site test in their data contain MNMs? If so, the authors should consider mentioning this in the manuscript. I do not think this will impact the results because the authors have used proper controls (non-shifting sister lineages), and it will be likely that all branches tested would suffer equally from MNMs. I only say this to make the authors aware of it, not to specifically request it be used (because I haven't had a chance to test its efficacy yet either), but HyPhy recently released a method that compares models with and without MNMs: https://github.com/veg/hyphy-analyses/tree/master/FitMultiModel.

Thank you for this comment. In our study, we did not consider multi-nucleotide mutations (MNMs), although we expect that the effect of MNMs is shared between control and host-plant shift branches because we designed the control branches to be similar to the host-plant shift branches (i.e. in terms of branch length and depth inside the topology). Because the use of HyPhy would be very computationally intensive and given the fact that we have used proper controls, we decided to add an acknowledgement in the revised text to mention this point, and we now cite the MNMs study.

Minor points

1. Figure 2: I think the y-axis could be labeled more descriptively for those unfamiliar with Bayesian sampling methods.

"Bayesian probability density" has been changed to "Frequency".

2. Line 564: Was the Aristolochiaceae phylogeny also inferred first with IQ-tree? I don't see that mentioned here.

The phylogeny of Aristolochiaceae was only reconstructed under Bayesian inference with MrBayes.

3. Line 801: It is clear to me from the main text and Figure 4 that you are comparing lineages with a host-plant shift to those without, but on this line it is ambiguous. Perhaps consider the following wording:

"We tested if branches representing inferred host-plant shifts along the phylogeny of swallowtails have more genes with dN/dS > 1 than lineages that did not have an inferred shift."

Thank you. We have modified this sentence following your recommendation.

4. Line 862: "Overall, given the our alignment" should read "Overall, given our alignment". Corrected accordingly.

Reviewer #2 (Remarks to the Author):

Title:
Genome-wide macroevolutionary signatures of key innovations in butterflies colonizing new host plants

Summary
The authors have brought together diverse datasets and analyses to study the evolutionary consequences of plant-insect interactions in a classic group of species. What is novel and important here is the extension of plant-insect coevolutionary studies to the genomic scale, to begin using an unbiased approach to searching for evolutionary consequences of these interactions. In order to provide the proper context for this type of analysis, the authors generate time calibrated phylogenies for the plant and insect clades, followed by assessing the spatial and temporal context of these clades during their early evolution, finding that these plants and insects arose close in time and geographic space. They further mapped the hostplants that the butterflies use upon the butterfly phylogeny, reconstructed their ancestral states and were thus able to place major hostplant shifts in a temporal context, against which they assessed butterfly species diversification rates in light of hostplant shifts. This revealed some dramatic increases in speciation associated with such shifts, as expected by coevolutionary theory. Then, using a previously published large dataset of low coverage genomes, they assessed whether host shifts were associated with an increase in signatures of macroevolutionary positive selection using ~1500 genes, finding significantly more genes under positive selection in branches associated with shifts to novel hostplants (as compared to control branches).

Thank you for your comments and very helpful extended feedback.

At face value, this is an important and significant step forward in the study of plant insect interactions, testing the expectations of coevolutionary interactions. However, there are a number of issues that need to be resolved before I think this is ready for a journal such as Nature Communications. If the authors are able to address these issues sufficiently, I think this could make a significant contribution to the literature. However, some of these issues are very serious and at the core of the paper.

We appreciate this positive note on the fact that our study could be an "important and significant step forward in the study of plant insect interactions". We have taken into account all your comments and made the requested changes to improve the clarity, avoid overstatements, and add important aspects that you have suggested. We feel this has significantly improved the manuscript.

Major concerns.
Molecular tests of selection

The biggest question I have is with regards to what the authors have actually detected in their study of positive selection. First, given their model specifications, as I understand them, they have branches in their tree that are either involved with shifting to a novel hostplant vs. those that are not and are used as a control. They then estimate, for about 1500 genes, site branch values of omega and then determine those that are significant. Then they group these

significant genes by what type of branch they occurred upon (a shifting branch vs control branch), and they find more sig. genes on shift branches compared to controls.

We would like to clarify a few points regarding the approach we used. Using our ancestral state reconstruction for host plants, we were able to pinpoint the branches of the phylogeny along which a host-plant shift likely occurred. We also selected control branches without shifts but with approximately the same length and that were as deep in the phylogeny as the host-plant shifts. Then, we created two datasets, taking into account missing species. For the first dataset (*Dataset 1*), we selected the genes for which all branches were available (i.e. a gene must have a phylogeny that includes all host-plant shifts and control branches) leading to 520 genes. For the second dataset, we selected all genes available for a given branch and we did that for the branches of interest. The second dataset selection leads to more genes (1,533 genes) but some of them have missing branches. Once the two datasets were created, we tested for positive selection using the branch-site model of CODEML for each gene and for each branch (see below for additional replies to your comments on this aspect). In other words, we tested all genes available for a given branch and did that for all the branches independently (14 branches host-plant shifts and 14 control branches). For the second dataset (*Dataset 2*), it is also important to compare the proportion of genes under positive selection instead of just looking at the number of genes under positive selection (as this number may vary from branch to branch).

Hence, as you said, we found a higher proportion of genes under positive selection on branches with host-plant shifts compared to control branches. However, we did not "*group these significant genes by what type of branch they occurred upon*", instead we estimated the trend of selection observed for each gene in all branches. For instance, it is possible to recover gene 1 as positively selected in branch X (host-plant shift branch) and in branch Y (control branch) and not in branch Z (another control branch). Doing so, overall, we found that more genes are positively selected when a host-plant shift occurs than when the host plant is conserved along control branches.

This leaves me with several questions.
Is the total number of genes reported under positive selection the number of genes with even just one site in one branch of their analysis? Did they look at the BEB values per site? Did all such genes have significant BEB values, or are they only using the lnL values at the gene level? What about genes with more than one site or more than one branch significant? Were these genes evenly distributed among branches or were some branches having more such genes? Complicating this, there is a lack of clarity and descriptions about what was done for this analysis. There is no summary table per gene for the lnL or BEB values, and I can't even find the raw totals of these analyses (genes per group, etc).

To address this comment, we need to clarify how we reported the genes under positive selection. Our strategy was to use the null and alternative hypotheses of neutral and adaptive protein evolution described in Zhang et al. (2005 - Mol. Biol. Evol.; see below our reply to your comment related to specifically running CODEML, section *Molecular tests of positive selection*). First, using CODEML, we test how the null hypothesis fits the evolution of a given gene in a given branch (the null hypothesis is composed of two parameters for the selection: sites under negative selection and site under neutral selection). Then, we compute the alternative hypothesis, in which a third parameter allows a proportion of sites to be under positive selection. CODEML estimates the likelihood of the two hypotheses. Then using a likelihood ratio test, we are able to determine which of the two models best fits the evolution of the gene in the given branch. Finally, given the number of genes, and therefore the number

of tests, we compute a Benjamini & Hochberg (1995)'s correction (FDR) to take into account the bias of multiple comparisons. After correction, a significant test means that at least a few sites are affected by positive selection along a particular lineage.

We did not use BEB values because that test is designed to identify specific sites with a high posterior probability of belonging to the positively selected category. Although tempting and potentially interesting, we recall that the goal of our CODEML analyses was to identify positively selected genes and not to study the sites underlying these adaptations. Indeed, even if, for example, looking at the amino-acid scale was of interest for finding functionally important sites, this finer-scale analysis is beyond the scope of our study.

Second, their analysis did not look across the entire genome. Rather it focused upon only a very small set of genes ~1500 and ~500, due to their low quality data and post filtering.

This comment could be applied to any study that analysed orthologous sequences between species – none of them could claim to analyse "the entire genome". It is nonetheless important to mention that our study looked "across" the entire genome because our genome-scale data traverses from one end to the other of genomes, using a method that has sampled relatively evenly across all regions of the genome. We did manage to analyse 5 to >10% of all protein-coding genes, which already represents a considerable effort as most sequences were newly generated, and these genes provide a representative sampling across the full genome. Moreover, given that the same set of orthologous genes is used for both types of branches (with or without host-plant shift), an increased number of genes would likely increase the power of our analysis.

*But, using even this small set they identified many genes under positive selection at every node in their phylogeny. If I am interpreting their findings correctly, they infer that out of random set of 1500 genes, they are detecting between 10 to 20 genes under positive selection at nearly EVERY branch across the entire phylogeny. This is true for even the 520 gene dataset (SF22), where there are about 26 branches, where on average 15 genes per branch were found significant, this is therefore 26*15 = ~390 genes identified as positively selected, which is > 50% of genes. Perhaps I have missed something, but if this is what the authors are claiming, well, that is simply not possible based upon the existing literature in comparative genomics when looking at random gene sets. Rather, this is suggestive of a high false positive rate arising from their use of low quality genomic datasets.*

We feel that the reviewer has misunderstood us here. We have thus clarified our point here as well as in the revised manuscript. As said above, a gene could be positively selected in many branches of the phylogeny. We indeed performed the test over 28 branches but the same genes were used for all branches (at least in *Dataset 1*). It is well known that some genes are particularly prone to fast evolution under positive selection (e.g. genes involved in immunity, Manna et al. 2019 - PNAS; Vianna et al. 2020 - PNAS). As a consequence these genes could be detected as positively selected in many branches. This is why we used control branches to discriminate the relevant genes specifically selected in the host-shift plants branches (Thomas & Hahn 2015 - Mol. Biol. Evol.; Zhou & Zhang 2015 - Mol. Biol. Evol.). As you can see in the new figure 4 (with the proportion of genes under positive selection instead of the number of genes), only 3.2% of the genes are positively selected on average per branch. Overall, 408 genes out of 1533 (26%) were detected as evolving under positive selection in at least one of the tested branches of the tree. This is substantially under the 390/520 (75%) of the genes that the reviewer claimed.

Along these lines, I want to applaud the authors efforts to assess these issues by conducting several posthoc analyses of their genomic data used in the molecular tests analyses. First, they do find evidence that missing data and GC3 content increase false positive rate in detecting positive selection (L831-833). Specifically, there is more missing data and higher GC3 levels in genes detected as being under positive selection, which is a hallmark of false positives. Great that they detected this, and they rightly point out that this could be a direct effect of lower quality data and GC biases. They then proceed to assess whether missing data and GC3 levels are significantly different between their compared groups, and find that they are not. This leads the authors to conclude that while the false positive rate is high, it is equally distributed among groups (e.g. host-shift vs. control), thus the differences between the two groups are meaningful – i.e. the higher number of genes under positive selection in the host shift group is biologically meaninful.

Thank you for recognizing our efforts to control our results by conducting several post-hoc analyses.

But, I have a different take away from these posthoc forensic analyses than the authors. In contrast, I conclude that the error rate is so high as to be problematic and to call into question the use of the low coverage dataset in general for this type of analysis.Going a bit deeper, when I look at your posthoc analyses of the codeML results, it looks like nearly your entire dataset has on average 10-15% missing data. I wonder what that means for your analyses. Doesn't it suggest that the genomic coverage is rather poor for many species? Gene regions should be the best assembled portion of the genome, thus this is high % of missing data is troubling. Moreover, what was the data quality in these regions that were used for analysis. Since they lack compete coverage, this suggests that the contigs themselves didn't have much coverage, and thus the assembly from which these exons were mined likely, on average, do not have had much sequence coverage and therefore have a high sequence error rate, as contig accuracy in genome assembly comes from high genomic coverage. Any such error rate inflation in these regions will be detected as positive selection given the increased likelihood of AA changes via such errors. Unless the authors can somehow improve their dataset to reduce these false positives, I find interpretation of their results very difficult at best. A suggestion would be if they were not just mining exons from draft genome assemblies, but also assessing the depth of sequencing coverage for each such exonic region, since if those regions have low coverage, they likely have high error and should not be used.

We appreciate the reviewer's concerns about the approach we have used, namely performing selection analysis based on low-coverage genomic data. To address the reviewer's concern, we measured the reads coverage for each gene and each species used in the study. The coverage was 26.7x on average for each species. The species with the lowest mean coverage is *Hypermnestra helios* with a coverage of 4.74x. We present a new Supplementary Table 2 to show the coverage per gene for each species, and the global average for the dataset.

Finally, the difference in missing data between branches with or without host-plant shifts is not significant (P = 0.83 / 1.00 for *Dataset 1* and *Dataset 2*, respectively, Mann-Whitney test; Supplementary Fig. 22). Additionally, there is no correlation between the number of genes under positive selection and the amount of missing data (P = 0.33 / 0.20 for the two datasets, respectively, Spearman's correlation test; Supplementary Fig. 23). So, once again, if missing data inflate the number of positive selections, it will do so in a similar fashion in both types of branches.

Also, the authors say a lot about their functional annotations, yet I find these have not be used to any significant extent in their analyses nor does this add much insight at all. Perhaps the authors could use their annotations in a more insightful way or push this more to the back of the MS.

The insight that emerges from our functional annotation analysis is that we don't know everything about non-model organism genes. This is the only thing that we discuss about these results and we believe it is still an important point. Finally, although we do not know the function of many of the genes, we show that many functional families are affected by the shifts. We think that this is sufficient to argue that the host-plant shifts are associated with changes in several functional families rather than only in detoxifying gene families or any other families. Even if that makes sense, the current literature, to the best of our knowledge, is really biased towards the study of detoxifying genes instead of looking for other genes that may be involved in insect-plant associations (e.g. Thompson 1988 - Evolution; Thompson et al. 1990 - Nature; de Medeiros & Farrell 2019 - bioRxiv). This is thus a general take-home message, which we hope will spur further research in this area.

Comparison of findings to previous work
The authors generated two time calibrated phylogenies, one for their butterflies and one for the major plant clade. How do their results compare to previous analyses, as this is never discussed by the authors, making it difficult to evaluate the accuracy and importance of this contribution.

We didn't think comparing phylogenies with previous works was relevant to the broad audience of *Nature Communications*. However, to account for the reviewer's remark we now provide a short comparison of our results with previous studies on both groups.

Also, and perhaps one of the most difficult issues the authors need to address are the findings of a recent study that documents the inherent danger in conducting the type of evolutionary analyses that are at the core of their paper (see Louca, S., and Pennell, M.W. (2020). Extant timetrees are consistent with a myriad of diversification histories. Nature 580, 502–505; and Pagel, M. (2020). Evolutionary trees can't reveal speciation and extinction rates. Nature 580, 461–462.). Specifically, estimating time dependent changes in speciation rate is potentially fraught with serious error and over interpretation. However, nowhere in the current MS do the authors show any circumspection regarding the potential errors in their analysis. I find that a troubling.

We are well aware of this issue, which dates back to long before Louca & Pennell (2020)'s study (e.g. Nee et al. 1994 - PTRSB; Harvey et al. 1994 - Evolution; Nee 2006 - Annu. Rev. Ecol. Evol. Syst.; Rabosky & Lovette 2008 - Evolution; Crisp & Cook 2009 - Evolution; Lambert & Stadler 2013 - Theor. Pop. Biol.). The problem raised by Louca & Pennell applies to LTT-based approaches but it remains unknown to which extent this issue is pervasive and extends to modern approaches based on branch lengths and tree topologies (e.g. Morlon et al. 2016 - Meth. Ecol. Evol.; Rabosky et al. 2013 - Nat. Commu.; Maliet et al. 2019 - Nat. Ecol. Evol.; Barido-Sottani et al. 2020 - Syst. Biol.). Importantly, the conclusions raised by Louca & Pennell have already been criticized (see Morlon et al. 2020 - bioRxiv). According to this latest study, Louca & Pennell neither undermine the hypothesis-driven model selection procedure widely used in macroevolution nor show that speciation and extinction dynamics cannot be investigated from extant timetrees using a data-driven procedure. Because we have used birth-death models in a very constrained set of hypotheses (i.e. change of diversification

associated with host-plant shifts), we think our conclusions are justified based on the inferences of the five different models. We have nonetheless added sentences in the main text to acknowledge possible issues with the estimation of speciation and extinction rates with phylogenetic trees.

L176-178: This is a strange argument. Given my understanding of the literature and plant insect interactions community, I don't think that anyone believes that single genes or a single gene family are enough to act as a key innovation. Rather, everyone believes that key innovations are complex phenotypes, ranging from hostplant preference, to detoxification, coupled with all the changes necessary for exploiting this resource due to the ecological dynamics involved. Researchers have focused upon specific genes cause that is all they could do to make the connection between genotype and phenotype, and no one to my knowledge is believing that these genes or gene familes was the whole story. Thus, this statement is really a false straw man that reflects misunderstanding upon the authors than the refences in question, or the community as a whole. I suggest rephrasing a bit to reflect the complexity of the issue. Certainly highlight your novel perspective and contribution here, but please don't use the existing straw man argument, as it doesn't reflect the literature in a way you would want to be remembered for.

We thank the reviewer for this comment, and have modified our manuscript accordingly. We agree that key innovations are probably complex phenotypes, ranging from host-plant choice, preference, larval performance, to detoxification, coupled with additional changes necessary for exploiting this resource. We also agree that this likely involves more than one gene or a gene family. Even May Berenbaum, who has been the pioneer on the role of CYP450, has argued that "*single genes, or even a single family of genes, almost certainly cannot be solely responsible for host shifts and subsequent specialization*" (Berenbaum et al. 1996 - Am. Nat.). Indeed it has been proposed that more genes are involved, even for Papilionidae, in which host finding and larval survival within a species, both necessary for a host shift, are genetically unlinked (Thompson 1988 - Evolution; Thompson et al. 1990 - Nature). Hence, we have rephrased the corresponding part to better reflect the literature consensus on the complexity of shifting to a new host plant.

Clarity of methods and datasets is lacking:
Beyond some of the issues I mentioned above, the reader needs to see summary statistics for dataset completeness for phylogenetic analysis of Papilio, as well as plant clades.
My preliminary look at their input datasets suggests that there is a large number of missing genetic content for many of their species. How does this affect their results? Usual analyses looks at these issues and quantify this effect.

The swallowtail species dataset alignment has 428 sequences with 5,873 sites including 3,878 distinct patterns, 2,595 parsimony-informative, 613 singleton sites, and 2,665 constant sites. In total, there is 32.2% of missing data. There have been many previous works that thoroughly analyzed and discussed the effect of missing data on the tree inferences (e.g. Wiens 1998, 2003 - Syst. Biol.; Agnarsson & May-Collado 2008 - Mol. Phylogenet. Evol.; Couvreur et al. 2010 - Mol. Biol. Evol.; Wiens & Morill 2011 - Syst. Biol.; Crête-Lafrenière et al. 2012 - PLoS One; Xi et al. 2016 - Mol. Biol. Evol. etc.). Most studies suggest that adding incomplete taxa may benefit phylogenetic accuracy under certain conditions (e.g. up to 70% of missing data).

Overstatement:

Language throughout the manuscript over interprets their findings. This occurs in several places. First, the authors argue that the butterfly and plant clades are "synchronous", which is difficult to assess and given their results do not appear to be synchronous to this reader. Rather they should be stating that they appear to have crown ages very close in time. Specifically, the peak of their posterior distributions are not identical (Fig1), nor should even such results be fully trusted as time calibrations are much like reading the entrails of chickens – so best to be circumspect here. What can be concluded is that temporal estimates for the two clades appear to be very close in evolutionary time.

At the macroevolutionary scale, we think that age estimates with such a similar median age and broad overlap of the 95% credibility interval is more than "close in time". In our results, we found that the origin of Papilionidae dates to 55.4 Ma (95% credibility intervals: 47.8-71.0 Ma) and the origin of *Aristolochia* dates to 55.5 Ma (95% credibility intervals: 39.2-72.8 Ma). As a comparison, in a strong insect-plant interaction system like in the case of the mutualistic interaction between figs and fig wasps, one would expect a synchronous origin or synchronous radiations between the two partners. Cruaud et al. (2012 - Syst. Biol.) estimated the figs originated 74.9 Ma (60.0 – 101.9 Ma) and the fig wasps originated 75.1 Ma (56.2 – 94.9 Ma). These age estimates and 95% credibility intervals are very comparable in terms of difference between the two clades. So we think that our results show an origin that is statistically indistinguishable from being synchronous between both groups and we think this is an important result that is important to state clearly.

Other example of overstatement are in regards to the phylogenetic tree, to the dates, to the reconstructed hostplants, historical ranges, to genes identified as being under positive selection – ALL of these are mere hypotheses. But, the authors use phrasing that states these findings as if they are somehow "the truth", e.g. L145 "showed more genome-wide molecular adaptations", L88 "results show that both Papilionidae and Aristolochia were ancestrally co-distributed", etc.

We have reworded the text to better represent inferential results. We are aware that our results are hypotheses, and we believe that readers will understand these results in the same light. We don't believe that "*showing more genome-wide molecular adaptations*" or "*showing that both Papilionidae and Aristolochia were ancestrally co-distributed*" means this is true. These sentences are plain statements about results that are, of course, estimates and consequently hypothetical. We have now paid additional attention to clarifying that our results are estimates and as such they are not the absolute truth.

L195: "our study reveals" is an overstatement that needs to be properly framed, along the lines of understanding that you have not shown anything, merely obtained sig Pvalues, which could be driven by many factors (e.g. model misspecification, sequence errors, all the issues that confound test of positive selection, etc). Without such perspective, your paper is grossly overselling its achievements.

We agree and we have changed "reveals" to "suggests" in this case. More generally, throughout the main text we have also replaced "reveal/reveals/revealed" with alternatives.

Diversification
I find it great that the authors used 5 different methods to get at their diversification dynamics. Wonderful. However, I can't find a proper discussion comparing these results and how you decided to report what you did. Specifically, the results from RPANDA and BAMM

for clade specific diversification rates through time appear to be quite different, with the BAMM results not agreeing with those from RPANDA. Rather than talking about this difference, the authors only use the RPANDA model results. I greatly appreciate the authors using both methods and reporting their findings, but this raises some complications with interpretation of the results. Also, it is not clear what results you are reporting in the main text compared to all the different analyses you ran.

Thank you for the positive comment on the diversification analyses. Given the difficulty in estimating rates of species diversification, it becomes crucial to use multiple methods to recover the rates, especially when we know the methodological concerns that pertain to birth-death models (e.g. Quental & Marshall 2010 - TREE; Rabosky & Goldberg 2015 - Syst. Biol.; O'Meara & Beaulieu 2016 - Am. J. Bot.; Moore et al. 2016 - PNAS; and Louca & Pennell 2020 - Nature, which is the latest of these criticisms). However, it is essential to recall that each method is designed to estimate speciation and extinction rates, sometimes with different data (e.g. tree alone, tree combined to trait data of extant species) and different mathematical foundations. As explained in the Methods section, each method differs at several points in the way speciation and extinction rates are estimated (Stadler 2013 - JEB; Morlon 2014 – Ecol. Lett.). For instance, trait-dependent birth-death models (diversitree) estimate constant speciation and extinction rates, while time-dependent birth–death models (BAMM and RPANDA) estimate the speciation and extinction rates and their variation through time. Therefore, we expect some differences between the values of the estimated diversification rates that are inherent to each approach. We used five methods to cross-test hypotheses and corroborate results. If there is a signal of host-plant on the diversification, we expect that several methods would recover the effect. Our diversification analyses should then be seen as complementary for the inferred diversification trend rather than corroborative on the values and magnitude of the speciation and extinction rates. In the revised version of the main text, we have made these differences clearer and we have included more text discussing the similarities and differences that are recovered in the estimates of diversification in light of our prior hypothesis.

In a similar vein, Fig. 3 does a good job of showing that the average rates per clade do not show a strong increase in diversification associated with hostplant shifts. These differences need to be tested statistically (L102-103) not just qualitatively reported, and it looks like most clades will not be different from the Aristolochiaceae-feeding swallowtails. But, your time dependent estimates of diversification show that these averages can be misleading (Fig3b). You should be discussing this difference between these two approaches and how the latter is more informative. Currently you present both as showing the same thing, which they do not. Further, why are the authors not showing their results for Fabaceae, Zygophyllaceae, Magnoliaceae? Is this because they have lower diversification rates after their shift, and therefore don't fit their expectations, or cause these clades are small? In sum, there appear to be as nearly as many clades increasing as decreasing in diversification post host shifts, if these are included ... Some explanation needs to be made as to why these clades are excluded from the main text and the general impression the authors are making in the main text, as what they are currently arguing doesn't appear supported by their own findings.

Figure 3 shows the results of two types of analyses: trait-dependent models with constant rates through time (panel a) and time-dependent models with rates varying through time and per clade (panel b). As discussed above, and now made clearer in the main text, the differences in rate estimates are expected. Trait-dependent models cannot show changes in

rates through time for a clade, so there is a smoothing rate for the whole under a constant model.

We did not show the results for Fabaceae, Zygophyllaceae, and Magnoliaceae because these host-plant shifts involved a tiny fraction (less than 1%) of the total swallowtail diversity. As explained in the *Methods* section, there is a single species (*Baronia brevicornis*) feeding on Fabaceae, two species of *Hypermnestra* feeding on Zygophyllaceae, and two species of *Teinopalpus* feeding on Magnoliaceae. So there are host-plant shifts that are unsuccessful from an evolutionary perspective, as their impact is negligible regarding the global evolutionary history of the family. We think that presenting these results will blur the main message; instead we present them as a Supplementary Figure 12. However, we have now expanded the main text to discuss the results of 'unsuccessful shifts', to be more comprehensive.

L105-107: "no observed classical slowdown of diversification". Give the authors own data, I disagree with this statement. Specifically, the results and the authors statements "A model with a slowdown of diversification through time explained the diversification of Annonaceae feeders, Lauraceae feeders, and Papaveraceae feeders (SF13). Clearly Lauracea and Papaveraceae, which are two with the largest increase, show a dramatic decrease in diversification rate through time. If you are looking at Papilio as a whole, perhaps this is an issue of temporal / species diversity scale.

This sentence was meant to report the *global* trend for Papilionidae, not for specific clades. As a whole, the Papilionidae do not show a slowdown of diversification. BAMM, RevBayes and RPANDA recovered this pattern (this is not possible to show this with the other models). However, when we take into account the possibility that rates may have been heterogeneous across the phylogeny, we do find that some clades' diversification has slowed down through time. We have rephrased to clearly say that there is no global slowdown, but that when taken individually, some clades show evidence of diversification slowdown after the host-plant shift.

Molecular tests of positive selection
CodeML specifications: I find your code a bit strange in a way that could inflate your estimates of omega. Why is your initial omega in your alternative analyses starting at 1.5, which would be strong positive selection as a prior? Won't this greatly bias your analyses? In the original control file, this is 0.4 and I've never seen this changed in an analysis. Why are you doing this? What happens if you use the default 0.4 for your alternative model?

We followed the recommendations of Zhang et al. 2005 (Mol. Biol. Evol.) and Ziheng Yang in his 2006 book on "*Computational Molecular Evolution*", specifically in Part III: Advanced Topics, and Chapter 8: Neutral and Adaptive Protein Evolution.

Here are the exact specifications to implement both the null and alternative hypotheses of neutral and adaptive protein evolution (see also Zhang et al. (2005; table 5) for the:

- Null hypothesis (branch site model A, with w2 = 1 fixed):
    model = 2, NSsites = 2, fix_omega = 1, omega = 1

- Alternative hypothesis (branch site model A, with w2 estimated):
    model = 2, NSsites = 2, fix_omega = 0, omega = 1.5 (or any value > 1).

Data availability:

This is insufficient details and datasets in the current MS, as well as upon the Figshare data repository, leaving the reader unable to assess the quality of the work performed.

Thus, this statement by the authors is incorrect at the time of my accessing the repository, "All data, including supermatrix datasets (for phylogenetic analyses), phylogenetic trees, host-plant preferences, species geographic distributions, gene alignments (for dN/dS analyses) and bioinformatic scripts, that are necessary for repeating the analyses described here have been made available through the Figshare digital data repository"

There are no gene alignments at this repository and many of the bioinformatic scripts are missing. For example, where are the R scripts used for analyses? (e.g. for the Lazer, MuSSE, RPANDAS, BAMM analyses?). Also, where is the table of codeML results per gene per site, lnL scores, etc? Further, while I am greatly excited by your use of genomic data from 45 species, the reader needs to know much more about the average statistics for these different genomes, in terms of coverage, assembly quality, gene coverage, etc., as you have gathered them all here for your paper, you should report upon their state in a unified fashion.

We have updated the Figshare link with a clearer structure. The gene alignments were actually available but perhaps not clearly visible in the former structure. In addition we have added the scripts for the analyses as well as the table for CODEML results per gene with the likelihood scores for both models and the likelihood ratio test.

Minor Comments
L41. citation for this?

We now cite two references for this well-established pattern: Stork (2018 - Annu. Rev. Entomol.) and Becerra (2015 - PNAS).

L49. evolutionary "linkages" .... This phrasing is unclear about the meaning, please restate.

This sentence has been rephrased.

L68. which family, plant or insect? More clarity please.

This has been corrected, and we meant plant family.

L77. "highly synchronous" is perhaps an overstatement, rather place these estimates into a more quantified statement for the reader to appreciate these findings.

We removed "highly". The age estimates are quantitative statements: "Divergence time estimates indicate synchronous radiation by Papilionidae (55.4 million years ago [Ma], 95% credibility intervals: 47.8-71.0 Ma) and Aristolochia (55.5 Ma, 95% credibility intervals: 39.2-72.8 Ma) since the early Eocene". As a comparison, in Cruaud et al. (2012 – Syst. Biol.) the mutualistic insect-plant interaction system between figs and fig wasps should also show synchronous radiations between the two partners. The authors estimated the figs originated 74.9 Ma (60.0 – 101.9 Ma) and the fig wasps originated 75.1 Ma (56.2 – 94.9 Ma). These age estimates and 95% credibility intervals are very comparable in terms of difference between the two clades.

L102. upshifts when talking in the same sentence as shifts in diversification .... That's a lot of shifts. Isn't "increase" good enough for one of these?

Following your recommendation, we corrected "upshifts" to "increases".

L146. not clear to me why there are two pvalues and why they are separated by a "/" symbol ... this is a bit confusing to me.

There are two *P* values because there are two genomic datasets (520 and 1,533 genes). We have clarified this aspect.

157-160. great that you checked these issues. You should also be including these aligned gene datasets for review as well as publication, as these need to be assessed.

The individual gene alignment of the genomic datasets were already included in the Figshare link, but we admit the folder was not easily identifiable. We have updated and expanded the Figshare folder with a better structure.

L165-166. you might want to restate this for clarity.

This sentence and the corresponding part have been rephrased.

Other issues that might have been covered above.

What were the exact commands used in iQtree?

The command line is the following:

```
./iqtree -s Papilionidae_408spp.phy -st DNA -spp Partitions_Papilionidae.txt -pre Papilionidae_408spp -m TESTNEW -allnni -nstop 500 -pers 0.2 -bb 2000 -nt AUTO
```

The details of the analyses have been added to the Methods section and in the Figshare repository along with the input files.

My Bayes run details. What where the minimum thresholds used for the ESS and PSRF values for each parameter?

We expect the ESS to be above or equal to 200 and PSRF to be the closest to 1.0. We have added these details in the Methods section.

What were the ESS minimum values from the BEAST temporal calibration analysis?

Similarly to MrBayes, the ESS should be greater or equal to 200 (threshold recommended by Drummond et al. 2006 - PLoS Biol.).

Where are the R scripts used for analyses? (e.g. for the Lazer, MuSSE, RPANDAS analyses?)

The scripts used for the diversification analyses have now been uploaded in the Figshare link.

BAMM analyses can be very sensitive to priors, great that you used 3 different CPP values. How did their convergence look, as I can't find any discussion of that.

This was indeed an issue with BAMM (Moore et al. 2016 - PNAS), but the developers have corrected this in the latest update of BAMM. We still have ensured that BAMM converged toward the same results regardless of the prior on the expected number of shifts. All the Bayesian analyses converged well (ESS > 200 for all three). They all show that there is no significant shift of diversification, although the posterior probabilities vary with the CPP. We have now made a table showing the results for the three analyses.

L169-171: you are highlighting the number of gene with unknown function by identifying total number with function. This makes for a strange sentence. Rather, say that 30% lacked annotation, ... But, I'm surprised that you had functional information for 70% of your genes. What thresholds on your annotations did you apply. For example, the authors for eggnog recommend using the cutoff of 200 on their scoring metric, rather then just using the annotations from all the genes that are returned. I imagine there should be some similar filter for PANZER. What this applied? Does it change these numbers?

We have rephrased the sentence accordingly. We found information for 70% of the genes thanks to the high-quality genome of *Papilio xuthus* (N50: 3.4Mb; see Lepbase database online). Finally, we wanted to attribute the genes to a GO family to test whether a go family was more often under positive selection than others. Again, for our study, this level of accuracy is highly sufficient to show that host-plant shifts affect a wide range of genes.

Also, when looking at your annotation table, even though you "annotation" from PANZER, there is no gene description. Is this cause there is only GO information? How general is that GO information, as I usually find it rather useless. Thus, please step back and have a second look at your annotations. I don't think they are as informative as you think. Also, do the gene annotations between PANZER and eggNOG agree? If you only looked at genes with descriptions, or informative GO assignemtn, how many genes actually have useful annotaitons. I'd be surprised if > 40% did ... thus I'm surprised you make a big deal of this, as I can't find anything to support these claims in your findings and you don't even use much of your annotation information at all in your results ...

We are not sure to know how to determine the difference between a useless or useful annotation. However, as specified in the previous reply above, our goal was to test whether a particular GO family played a more important role than other families. Hence all the GO families are useful for us in this analysis.

In your Table S2, you should also include your eggNOG score for annotation, just as you did the HMM e-value score for the PANZER annotation.

We have added the score, which is the mean seed eggNOG ortholog e-value, for each gene to the Supplementary Table 4.

Fig 4 is showing gene totals from which dataset?

Figure 4 formerly shows the number of genes for *Dataset 1* (520 genes), and we have now updated the figure with the percentage of genes for *Dataset 2* (1,533 genes). The results for Dataset 1 are available as Supplementary Figure 19. The results are very similar. We have now updated these figures to show the percentage of genes per branch type.

L180: "deeper adaptation" .... Can you please restate for greater clarity.

We have rephrased this part of the sentence.

L182-191. I suggest that here is a good place to say that your analyses have generated a list of novel candidates possibly involved in plant-insect interactions, which require functional validation, as what you have generated are a series of hypotheses.

This is a good point, and we have now incorporated this idea in the revised version of the manuscript.

L196. "more pervasive than previously recognized". I disagree with this statement. Rather, you study is the first to be able to look at this scale, and it simply is able to get at this question in a more comprehensive / global fashion. Everyone in the plant-insect community knows that there are certain to be many genes involved, as host plant shifts are complex.

We agree with this point, and we also recognize that it could be expected that many genes are involved during host-plant shifts. While May Berenbaum worked on CP450 during her career, she recognized that "*Single genes, or even a single family of genes, almost certainly cannot be solely responsible for host shifts and subsequent specialization*". We have modified the corresponding sentences to acknowledge that the plant-insect community expected this result given the complexity of host-plant shifts (e.g. Mitter et al. 1988 - Am. Nat.; Thompson 1988 - Evolution; Thompson et al. 1990 - Nature; Berenbaum et al. 1996 - Am. Nat.; Nallu et al. 2018 - Nat. Ecol. Evol.).

L198. "more adaptations than in just the gene families in detoxification pathway". This is falling into the same logical fallacy as earlier sentiments. Please revise. Again, suggest that you argue that your able to extend beyond the candidate gene/gene family perspective that others have previously used (cause that was all they had for study and focus – they didn't have the glorious dataset you have).

Similar to above replies, we have rephrased the corresponding sentence.

L200-202. Certainly, this is a true statement and I think the community expects this. You're the first to be able to gain such insights. Thus rather than saying you've discovered what is likely to be more general, I'd restructure your argument throughout the paper to be that this is expected, and here we provide evidence of it at the genomic scale.

We have modified the main text to convey the idea that there are expectations in the plant-insect community for more genes involved in host-plant shifts, but it was difficult to assess the extent of genome-wide adaptations across many genes before. Hence we have also deeply modified the conclusion section.

Figure 2. I suggest alternating the gray/clear color pattern for the epochs, such that your images of the hostplant and butterfly are on a white background rather than gray.

The gray/white color pattern follows official use of the geological time scale. Because the pictures of *Aristolochia* (host plant) and *Ornithoptera* (butterfly) are not the main take-home message of the figure, we would prefer to leave it as it is for clarity.

Fig. 3. I suggest having straight arrows or now arrows in panel B to show the shift, as this gives a strange impression of when the shift occurred. Horizontal lines would be better.

The host-plant shifts occur at the beginning of each diversification curve. Horizontal lines would not make the figure easier to read according to our modifications. We believe the figure is more accurate this way.

SM15. please include the species names on this tree so that the reader can explicitly see which branches on the tree are what in their analysis.

The figure has been updated to show species names.

L773. "for all consisting on all genera" is unclear, please restate this sentence for greater clarity.

This part of the sentence has been rephrased.

L771. a robust phylogenomic tree of Papilionidae. This is never demonstrated, but rather claimed. I find now tree for these 45 taxa that has bransh support, and even then, what is needed, given the dataset of > 6000 genes to build this, are reports of gene concordance factors (implemented easily in iQtree) to assess how well supported this tree actually is. Currently the reader is directed to SF18, but this lacks taxa labels and branch support information. Given the low gCF and sCF in the Allio et al. 2020 paper, I assume these are similar here, and thus, calling this phylogeny robust is inappropriate as there remains substantial potential discordance in the nodes.

The phylogenomic tree of Papilionidae is robust. We note that this tree has been reconstructed with 6,621 genes and with many different techniques taking into account a number of known biases for tree inferences, which has been explained in detail and published by Allio et al. (2020 - Syst. Biol.). The gCF and sCF are complementary to more classical gene-tree reconciliation approaches such as ASTRAL and SuperTriplets, which all allow pinpointing possible nodes where ancient ILS or hybridization occurred. Supermatrix and supertree approaches all yielded the same topology, and identify two nodes for which we suspect ILS or hybridization for *Lamproptera – Iphiclides* (ASTRAL node score: 44.2 / SuperTriplets node score: 45), and hybridization for *Cressida*, *Parides* and *Euryades* (ASTRAL node score: 63 / SuperTriplets node score: 64). These phylogenetic relationships do not impact the inferences made with dN/dS analyses or with birth-death models.

How did the BAMM model specifications of priors influence results? This was never properly discussed, as it usually has a very large effect.

According to our analyses testing the effect of priors on posteriors, there is no influence of the prior as we find the same results of global diversification dynamics. Also, it is important to remind that the BAMM developers have corrected the influence of "model specifications of priors" (see Rabosky et al. 2017 - Syst. Biol.).

What is the false discovery rate used for the dN/dS analysis of the codeML results?

This is the FDR proposed by Benjamini & Hotchberg (1995 - J. R. Stat. Soc. B). This has been included in the revised manuscript for clarity.

What value you were using for the pvalue for the per gene omega?

We used the *P*-value of the likelihood ratio test (between the two models) after FDR correction. We then considered that a gene was under positive selection for a given branch if the *P*-value was at the significance threshold of 0.05, as usually done.

Reviewer #3 (Remarks to the Author):

The authors present a highly interesting study on the drivers of diversification in the butterfly family Papilionidae. It is one of the first to link changes in host plant use and range shifts to new biomes to changes in genomic signatures of selection. The data used to investigate these connections is comprehensive and adequate for the questions asked. As I am not an expert in functional genomics, I will restrict my comments to the phylogenetics, timing of divergence and diversification analyses. These have been done rigourously, as one has come to expect from the Condamine lab. The authors use the latest methods, and justify their use of the currently contentious analyses of diversification dynamics based on molecular phylogenies. With the addition of the historical biogeography analysis (also well done), these analyses form the bases for what is the main news of the study, the genomic signatures of selection associated with changes in host plant use. I leave it to reviewers with functional genomic expertise to tell us whether those analyses are done according to standards and whether interpretations of results are correct, but the bases for those analyses are solid.

Signed,
Niklas Wahlberg

Thank you for the positive input, which is much appreciated. As you might have seen from the two other reviewers with more genomic expertise, there have been some concerns raised during their evaluation. We think we have successfully addressed all the points. Overall, these comments have improved the clarity and interpretations of the genome-based analyses. We hope this will also convince you that the study and conclusion are more robust.

In summary, we have taken into account all comments and corrections brought up by the referees, including thoroughly revising the manuscript, tables and figures, and also the supplementary information. All co-authors have contributed to reading and revising this manuscript. We hope you will find our revision appropriate and sufficient to justify its acceptance, and we are enthusiastically looking forward to receiving your response.

Yours sincerely,

Rémi Allio and Fabien Condamine (On behalf of all co-authors)

Reviewer #1 (Remarks to the Author):

The authors have sufficiently addressed my concerns and I feel the work is now ready for publication. I wish them luck!

Reviewer #2 (Remarks to the Author):

I have just finished my reading of the authors response to all the reviews, as well as reading through the revised manuscript.

well done authors.

I had a wide range of concerns, some more difficult than others, ranging from technical to ... philosophical. You responded to all of the in a very professional and insightful manner. The revision is much improved, in diverse ways. I greatly appreciate your shift in language, argumentation, and results analysis.

this will make an excellent contribution to the literature and I look forward to this getting some nice press.

well done indeed.

signed
christopher wheat

Reviewer #3 (Remarks to the Author):

I am happy with the revision, I have no further comments.
